# miR‐181a/b downregulation: a mutation‐independent therapeutic approach for inherited retinal diseases

**DOI:** 10.15252/emmm.202215941

**Published:** 2022-10-04

**Authors:** Sabrina Carrella, Martina Di Guida, Simona Brillante, Davide Piccolo, Ludovica Ciampi, Irene Guadagnino, Jorge Garcia Piqueras, Mariateresa Pizzo, Elena Marrocco, Marta Molinari, Georgios Petrogiannakis, Sara Barbato, Yulia Ezhova, Alberto Auricchio, Brunella Franco, Elvira De Leonibus, Enrico Maria Surace, Alessia Indrieri, Sandro Banfi

**Affiliations:** ^1^ Telethon Institute of Genetics and Medicine (TIGEM) Pozzuoli Italy; ^2^ Ecosustainable Marine Biotechnology Department Stazione Zoologica Anton Dohrn Naples Italy; ^3^ Medical Genetics, Department of Precision Medicine University of Campania “L. Vanvitelli” Naples Italy; ^4^ Molecular Life Science, Department of Science and Environmental, Biological and Farmaceutical Technologies University of Campania “Luigi Vanvitelli” Naples Italy; ^5^ Medical Genetics, Department of Advanced Biomedicine University of Naples “Federico II” Naples Italy; ^6^ Medical Genetics, Department of Translational Medical Science University of Naples “Federico II” Naples Italy; ^7^ Scuola Superiore Meridionale, School of Advanced Studies Naples Italy; ^8^ Institute of Biochemistry and Cellular Biology (IBBC) National Research Council (CNR) Monterotondo, Rome Italy; ^9^ Institute for Genetic and Biomedical Research (IRGB) National Research Council (CNR) Milan Italy

**Keywords:** inherited retinal diseases, miR‐181, mitochondria, photoreceptor, therapy, Genetics, Gene Therapy & Genetic Disease, Neuroscience, RNA Biology

## Abstract

Inherited retinal diseases (IRDs) are a group of diseases whose common landmark is progressive photoreceptor loss. The development of gene‐specific therapies for IRDs is hampered by their wide genetic heterogeneity. Mitochondrial dysfunction is proving to constitute one of the key pathogenic events in IRDs; hence, approaches that enhance mitochondrial activities have a promising therapeutic potential for these conditions. We previously reported that miR‐181a/b downregulation boosts mitochondrial turnover in models of primary retinal mitochondrial diseases. Here, we show that miR‐181a/b silencing has a beneficial effect also in IRDs. In particular, the injection in the subretinal space of an adeno‐associated viral vector (AAV) that harbors a miR‐181a/b inhibitor (sponge) sequence (AAV2/8‐GFP‐Sponge‐miR‐181a/b) improves retinal morphology and visual function both in models of autosomal dominant (*RHO‐P347S*) and of autosomal recessive (*rd10*) retinitis pigmentosa. Moreover, we demonstrate that miR‐181a/b downregulation modulates the level of the mitochondrial fission‐related protein Drp1 and rescues the mitochondrial fragmentation in *RHO‐P347S* photoreceptors. Overall, these data support the potential use of miR‐181a/b downregulation as an innovative mutation‐independent therapeutic strategy for IRDs, which can be effective both to delay disease progression and to aid gene‐specific therapeutic approaches.

## Graphical abstract

The application of gene‐specific approaches for the treatment of inherited retinal diseases (IRDs) is hampered by their broad genetic heterogeneity. Modulation of the expression of miR‐181a/b in the retina represents a promising gene‐independent therapeutic strategy for these conditions.

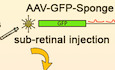

**The paper explained**

**Problem**

Inherited retinal dystrophies (IRDs) are a group of genetic disorders affecting the retina characterized by photoreceptor cell death, progressive loss of vision and blindness. Their high genetic heterogeneity, with over 250 different causative genes, represents an important limitation to the development of gene therapy approaches that can be applied to a significant number of patients. Therefore, the establishment of therapeutic approaches for IRDs independent on the genetic defect and aimed at slowing down disease progression represents a high priority.

**Results**

Here, we show that the inhibition in the retina of two small RNA molecules, namely microRNAs miR‐181a and miR‐181b (miR‐181a/b), exerts a beneficial effect *in vivo* in different models of IRDs, by delaying disease progression. We also observed a beneficial effect on mitochondria defects observed in these IRDs models.

**Impact**

Our findings represent a solid proof‐of‐principle of the usefulness of miR‐181a/b inhibition to counteract disease progression in IRDs and pave the way toward the development of an innovative gene‐independent therapeutic strategy for retinal diseases with mitochondrial involvement.

## Introduction

Inherited retinal diseases (IRDs) are among the most prevalent causes of vision loss/blindness of genetic origin in the working‐age population. They encompass a variety of different clinical subtypes such as retinitis pigmentosa (RP), Leber congenital amaurosis (LCA), and macular diseases. IRDs display extensive genetic heterogeneity with more than 250 causative genes identified thus far (https://sph.uth.edu/retnet/disease.htm). Gene‐replacement therapies are proving to be effective in the treatment of IRDs caused by loss‐of‐function mutations as exemplified by the recent approval of Voretigene Neparvovec for the treatment of conditions caused by mutations in the *RPE65* gene (Ledford, 2017; Apte, 2018). Unfortunately, gene replacement cannot be effectively applied to IRDs caused by gain‐of‐function mutations, in which the silencing of the mutated allele is primarily required. Moreover, the high genetic heterogeneity of IRDs significantly limit the set up of gene/mutation‐specific approaches that can be applied to a large fraction of patients (Carrella *et al*, 2020).

Gene/mutation‐independent therapeutic strategies that aim at decreasing and/or delaying cell death in the affected retina, independently of the primary genetic defect, constitute a valid and/or complementary alternative to gene‐based approaches (Carrella *et al*, 2020). Degeneration of photoreceptors (PRs) is the common landmark in IRDs even though a precise knowledge of the underlying molecular and cellular events is still far from reach. Among the most relevant pathways involved, mitochondrial dysfunction, neuroinflammation, and microglia activation are known to worsen PR death and accelerate disease progression (Ambati *et al*, 2013; Cuenca *et al*, 2014; Zhao *et al*, 2015; Lefevere *et al*, 2017; Mirra & Marfany, 2019; Carrella *et al*, 2021). Targeting key effectors that impact on common dysregulated pathways in neuronal damage during disease progression hold great promise for therapeutic purposes (Indrieri *et al*, 2019; Carrella *et al*, 2020). In the latter respect, microRNAs (miRNAs), given their ability to simultaneously modulate multiple molecular pathways commonly associated with disease pathogenesis and progression, constitute appealing targets toward the design of gene/mutation‐independent therapeutic strategies (Askou *et al*, 2018).

We recently identified miR‐181a and miR‐181b (miR‐181a/b) as possible therapeutic targets for mitochondrial‐related retinal diseases (Indrieri *et al*, 2019). MiR‐181a/b are highly expressed in the central nervous system (CNS) and in different retinal cell types (Busskamp *et al*, 2014; Indrieri *et al*, 2019). They are part of the miR‐181 miRNA family and are localized in two distinct genomic clusters in mammals (*miR‐181a/b‐1* and *miR‐181a/b‐2*). The mature forms of miR‐181a and miR‐181b produced by both clusters show identical sequences. Furthermore, the “seed” sequence, that is, the domain hypothesized to play the most relevant role in target recognition, is identical between miR‐181a and miR‐181b (Indrieri *et al*, 2020). Several mitochondrial‐related transcripts are direct targets of miR‐181a/b, for example, *Bcl2*, *Mcl1*, and *Park2* (Ouyang *et al*, 2012; Cheng *et al*, 2016). We recently demonstrated that miR‐181a/b control mitochondrial turnover and function *in vivo* by targeting genes involved in mitochondrial biogenesis, function, and clearance, as well as in reactive oxigen species (ROS) detoxification (i.e., *Tfam*, *Nrf1*, *Cox11*, *CoQ10B*, and *Prdx3*) (Indrieri *et al*, 2019; Barbato *et al*, 2021). Genetic inactivation of *miR‐181a/b‐1* leads to increased levels of mitochondrial biogenesis and mitophagy in the retina of mouse models of primary mitochondrial dysfunction leading to strong protection from neuronal cell death and to significant amelioration of the disease phenotype (Indrieri *et al*, 2019). In particular, we showed that genetic inactivation of *miR‐181a/b‐1* protects retinal ganglion cells (RGCs) and ameliorates visual function in different mouse models of Leber's hereditary optic neuropathy (LHON) strongly indicating that these miRNAs may represent effective therapeutic targets for this disease (Indrieri *et al*, 2019). Notably, we also demonstrated that *miR‐181a*/*b‐1* ablation did not cause *per se* neither abnormalities in retinal morphology nor alteration of retinal function, thus supporting the safety of miR‐181a/b downregulation in the retina (Indrieri *et al*, 2019).

Over the last decades, evidence linking mitochondrial dysfunction to IRDs is increasing (Carrella *et al*, 2021). The outer retina is known to have a high metabolic demand, which is associated with abundance of mitochondria in the retinal pigment epithelium (RPE) and PRs. The latter cells are under constant environmental challenges and are highly prone to oxidative stress, a major contributor to retinal degenerations and hence IRDs (Lefevere *et al*, 2017; Mirra & Marfany, 2019; Jiang *et al*, 2022). Importantly, since mitochondrial dysfunction seems to be an early event in IRDs, approaches targeting basic mitochondrial functions hold great therapeutic promise (Lefevere *et al*, 2017; Carrella *et al*, 2021).

Here, we tested the possible neuroprotective effect of miR‐181a/b downregulation in IRDs. We demonstrated that miR‐181a/b downregulation protects PRs from death and ameliorates their morphology, resulting in improvement of visual function in two different IRD models. We also observed amelioration of mitochondria morphology in PRs, along with a concomitant downregulation of the mitochondrial fission protein Drp1 mediated by the activation of the JAK2/STAT3 pathway. Our data unveil a novel molecular mechanism by which miR‐181a/b regulate mitochondrial morphology and function in diseased conditions and identified an AAV‐mediated sponge strategy to efficiently downregulate miR‐181a/b *in vivo*. We demonstrated that miR‐181a/b downregulation could be effective in ameliorating retinal function across different IRD models with a mutation/gene‐independent mechanism, thus providing a novel potential therapeutic strategy that deserves further development toward clinical application in patients.

## Results

### miR‐181a/b‐1 downregulation slows down retinal degeneration in P347S mice

**Figure 1:**
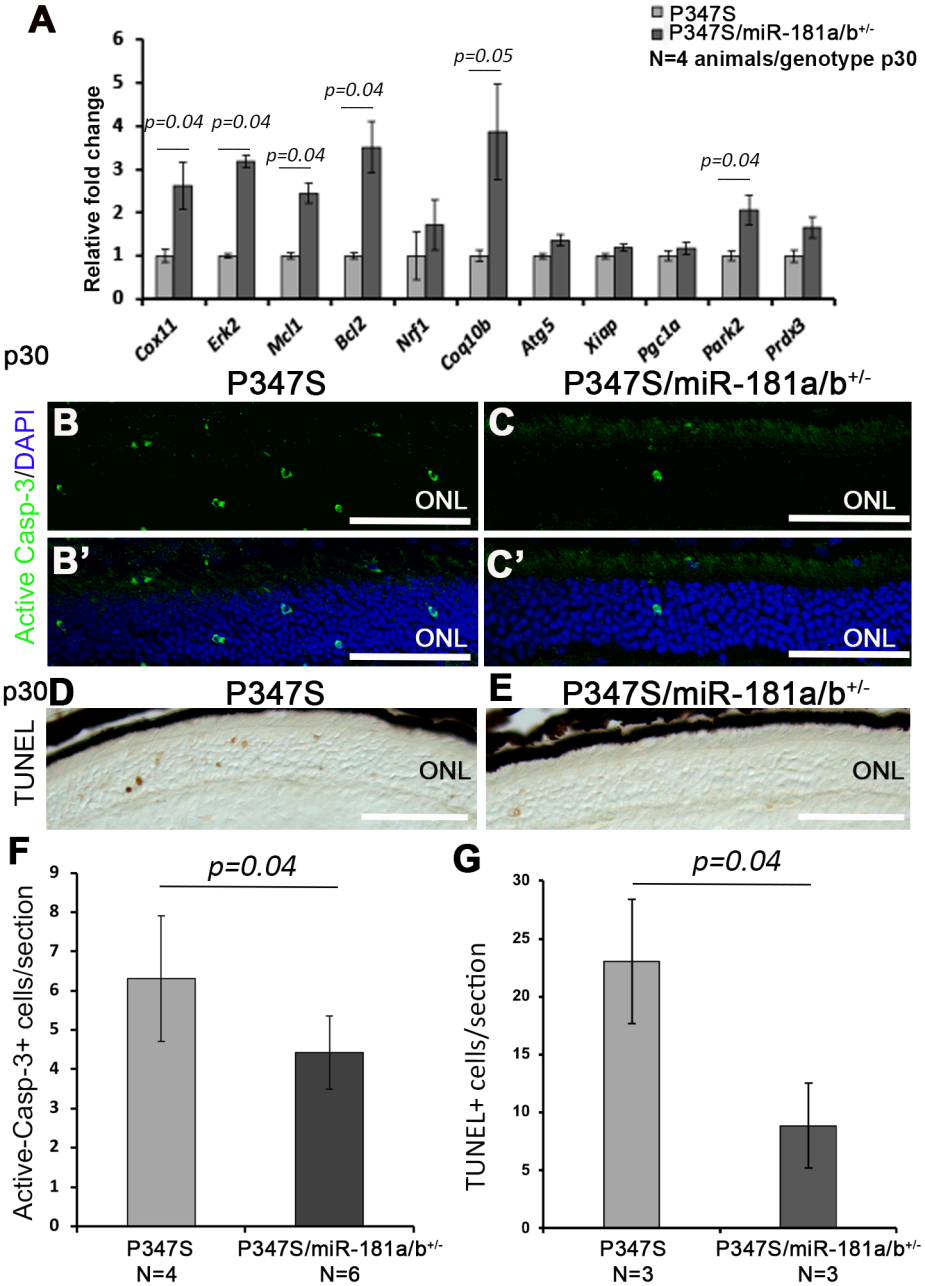
miR‐181a/b‐1 downregulation slows down retinal degeneration in P347S mice at p30 AqRT–PCR analysis reveals increased levels of most of the miR‐181a/b targets in the eyes of P347S/miR‐181a/b^+/−^ versus P347S animals. *N* = 4 animals/genotype.B–C′Immunofluorescence analysis of active‐caspase 3 in the ONL of P347S and P347S/miR‐181a/b^+/−^.D, ETUNEL analysis in the ONL of P347S and P347S/miR‐181a/b^+/−^.FQuantification of immunofluorescence analysis in (B–C′) (*N* = 4 eyes P347S and *N* = 6 eyes P347S/ miR‐181a/b^+/−^).GQuantification of TUNEL analysis in (D, E) (*N* = 3 eyes/genotype). Data information: Scale bars are 50 μm. Data are presented as mean ± SEM. Student's *t*‐test unpaired. [Colour figure can be viewed at wileyonlinelibrary.com]

The transgenic line carrying the proline‐347 to serine (P347S) mutation in the Rhodopsin (RHO) protein is a model for autosomal dominant (AD) RP. These animals show a reduction in electroretinogram (ERG) amplitudes that correlate with the extent of PR loss (Li *et al*, 1996). The P347S mutation leads to mis‐trafficking of the RHO protein with defective vectorial transport of post‐Golgi vesicles, which mostly fail in reaching the nascent disks of the PR outer segment (OS) (Li *et al*, 1996; Greenwald *et al*, 2013) and is therefore mis‐localized to the outer nuclear layer (ONL) in the transgenic mouse retina (Chadderton *et al*, 2009; Marrocco *et al*, 2021; Patrizi *et al*, 2021). A reduced ERG response in *RHO‐*P347S mice (from now onward termed P347S) can be observed at postnatal day (p) 30 and, albeit severely impaired, can be recorded up to p70–90. To study the effect of miR‐181a/b inactivation on RP progression, we crossed *miR‐181a*/*b‐1* ^+/−^ (Henao‐Mejia *et al*, 2013; Indrieri *et al*, 2019) with P347S^+/+^ mice to obtain, in the same litters, P347S^+/−^/miR‐181a/b‐1^+/+^ and P347S^+/−^/miR‐181a/b‐1^+/−^ animals, termed P347S and P347S/miR‐181a/b‐1^+/−^, respectively (see Materials and Methods). Most of the analyzed miR‐181a/b targets (i.e., *Cox11*, *Erk2*, *Mcl1*, *Bcl2*, *Nrf1*, *Coq10b*, *Atg5*, *Xiap*, *Pgc1a*, *Park2*, and *Prdx3*) displayed increased transcript levels in P347S/miR‐181a/b‐1^+/−^ versus P347S eyes, as measured by quantitative (q)RT–PCR (Fig 1A).

**Figure 2:**
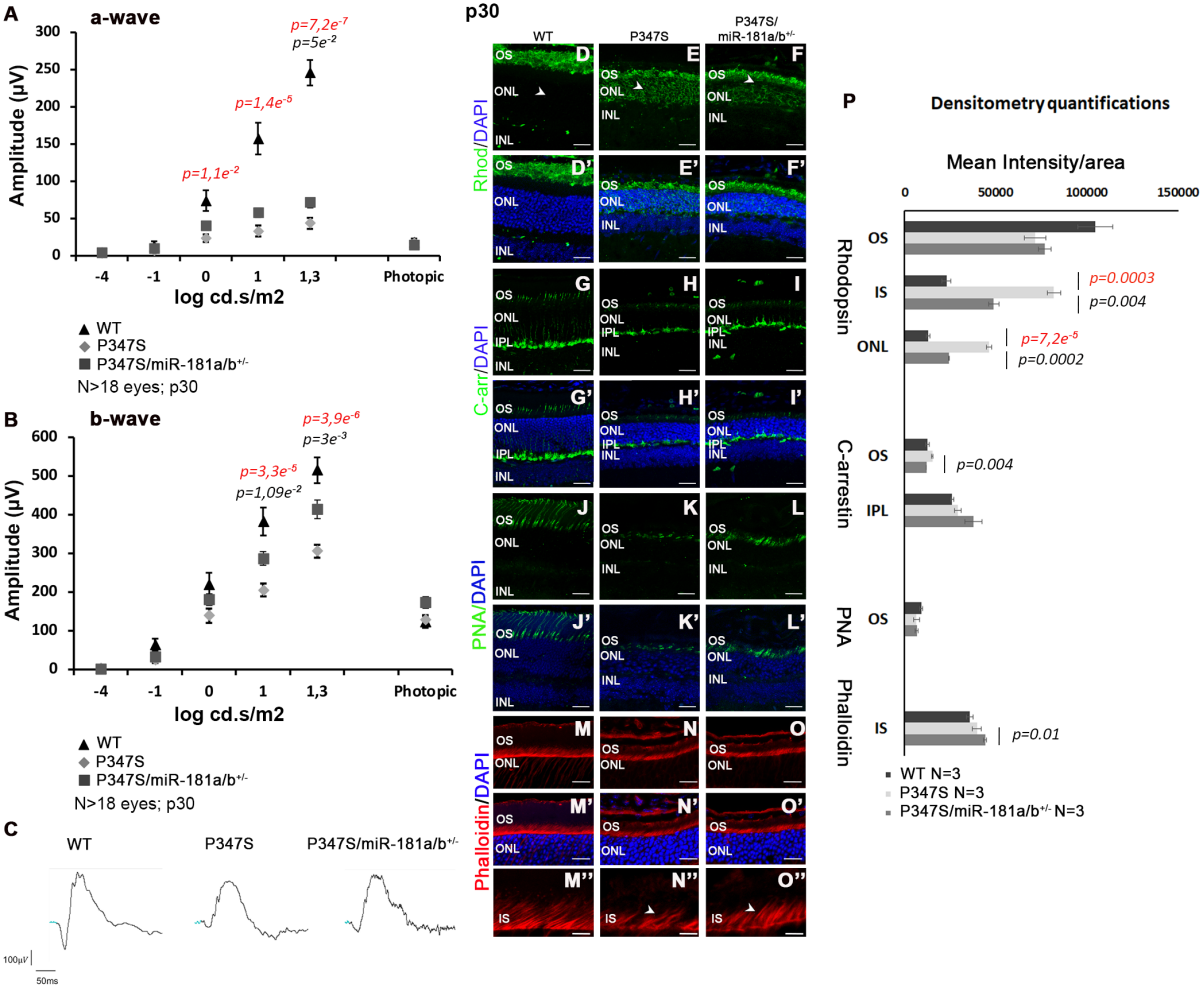
miR‐181a/b‐1 downregulation ameliorates the retinal phenotype in P347S mice at p30 A–CERG response [a‐wave in (A) and b‐wave in (B)], recorded in scotopic conditions, of WT, P347S and P347S/miR‐181a/b^+/−^ animals at p30; *N* ≥ 18 eyes. Data are presented as mean ± SD. Two‐way ANOVA test. Representative curves are reported in (C).D–PImmunofluorescence analysis showed amelioration of Rhodopsin localization (D–F′; white arrowheads); C‐arrestin expression (G–I′) and PR outer segment (OS) and inner segment (IS) structures, as determined, respectively, by PNA (J–L′); and Phalloidin staining (M–O″) in P347S/miR‐181a/b^+/−^ versus P347S eyes at p30. (M″–O″) show higher magnification of (M–O). Scale bars 25 μm in (D–O′); 5 μm in (M″–O″). Fluorescence densitometry quantification of each staining is reported in (P), *N* = 3 eye/genotype for each staining. WT versus P347S *P*‐values are reported in red, P347S versus P347S/miR‐181a/b^+/−^ *P*‐values are reported in black. Data are presented as mean ± SEM. Student's *t*‐test, unpaired. [Colour figure can be viewed at wileyonlinelibrary.com]

Cell death was analyzed in the retinas of P347S and P347S/miR‐181a/b‐1^+/−^ animals by active caspase‐3 staining and TUNEL assays at p30. A significant decrease of cell death in the ONL of P347S/miR‐181a/b‐1^+/−^ with respect to P347S was observed (Fig 1B–G). At the same time point, we also detected a significant improvement of ERG responses (both a‐waves and b‐waves) registered in scotopic conditions in P347S/miR‐181a/b‐1^+/−^ compared with P347S eyes (Fig 2A–C).

**Figure EV1:**
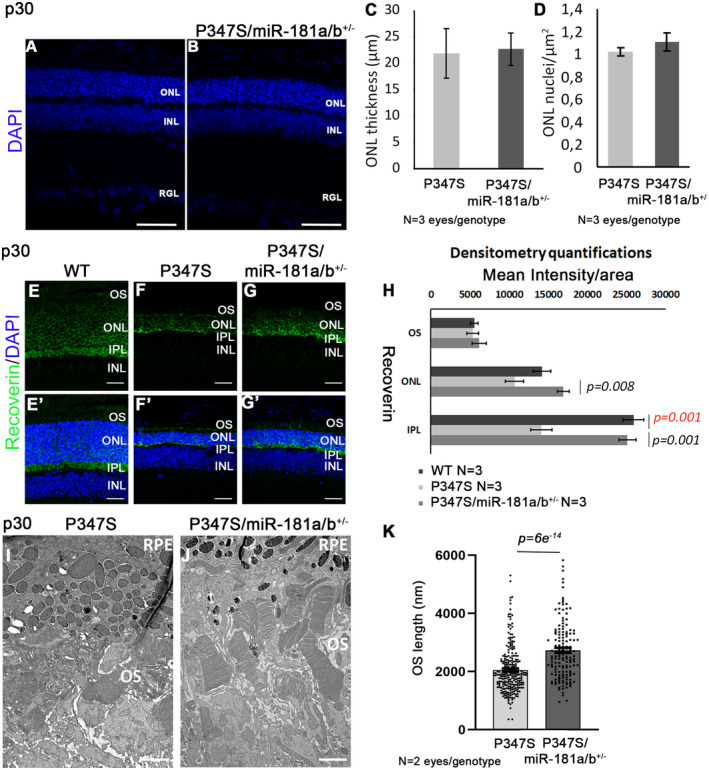
Effects of miR‐181a/b‐1 downregulation on PRs in P347S mice at p30 A–DDAPI staining of P347S (A) and P347S/miR‐181a/b^+/−^ (B) retinas highlighted no difference in ONL thickness and number of nuclei in the ONL, as quantified in (C) and (D). *N* = 3 eyes/genotype. Data are presented as mean ± SEM. Student's *t*‐test, unpaired. Scale bars are 50 μm in (A, B).E–HImmunofluorescence analysis showed amelioration of Recoverin staining in the ONL and the Inner Plexiform Layer (IPL) in P347S/miR‐181a/b^+/−^ versus P347S eyes at p30. Scale bars 25 μm. Fluorescence densitometry quantification of Recoverin staining is reported in (H), *N* = 3 eye/genotype. WT versus P347S *P*‐values are reported in red, P347S versus P347S/miR‐181a/b^+/−^ *P*‐values are reported in black. Data are presented as mean ± SEM. Student's *t*‐test.I–KElectron microscopy analysis shows increased length of PR OS in P347S/miR‐181a/b^+/−^ versus P347S, quantified in (K) (*N* = 2 animals/genotype); Data are presented as mean ± SEM. Student's *t*‐test, unpaired; Scale bars are 1 μm in (I, J).

Although we did not observe significant differences in ONL thickness at p30 (Fig EV1A–D), the expression of the PR markers Rhodopsin (Fig 2D–F′), C‐Arrestin (Fig 2G–I′), and Recoverin (Fig EV1E–H) was better preserved in P347S/miR‐181a/b‐1^+/−^ eyes as compared with P347S controls. In particular, we noticed amelioration of: (i) Rhodopsin distribution, which displayed a lower extent of retainment in the ONL (Fig 2D–F′ and P); (ii) structure of cone and rod OS, as revealed by PNA staining (Fig 2J–L′) and by measurement of OS length on electron microscopy (EM) images (Fig EV1I–K); and (iii) inner segment (IS) structures, as highlighted by Phalloidin staining (Fig 2M–P).

**Figure 3:**
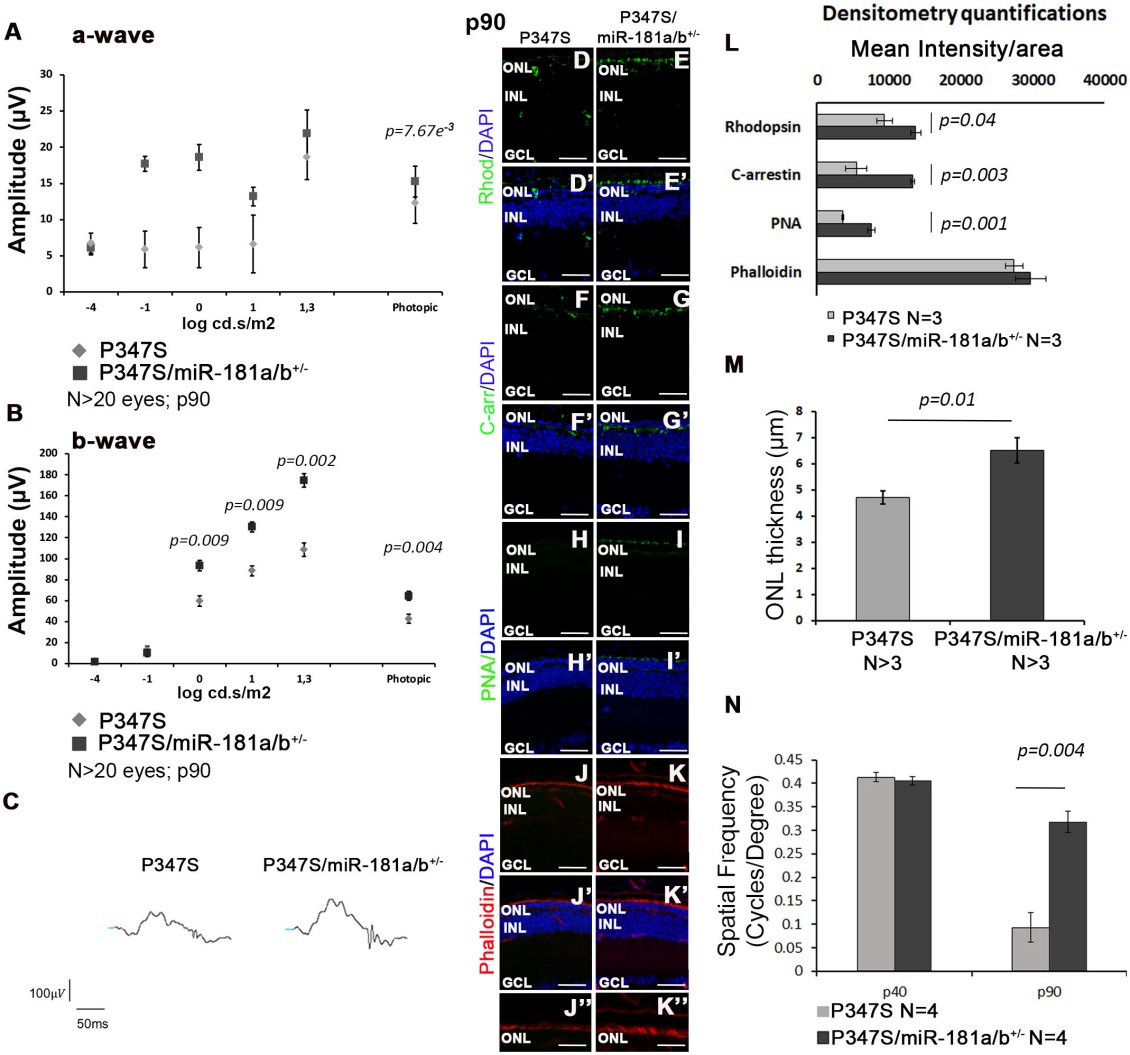
The effect of miR‐181a/b‐1 downregulation in P347S mice is still detectable at p90 A–CERG response [a‐wave in (A) and b‐wave in (B)], recorded in scotopic conditions, of P347S and P347S/miR‐181a/b^+/−^ animals at p90; *N* ≥ 20 eyes/genotype. Data are presented as mean ± SD. Two‐way ANOVA test. Representative curves are reported in (C).D–LImmunofluorescence analysis of Rhodopsin (D–E′), C‐arrestin (F–G′), PNA (H–I′); and Phalloidin staining (J–K″) in P347S and P347S/miR‐181a/b^+/−^ retinas at p90. (J″–K″) show higher magnification of (J–K). Scale bars 25 μm in (D–K′); 10 μm in (J″–K″). Fluorescence densitometry quantification of each staining is reported in (L), *N* = 3 eye/genotype/staining. Data are presented as mean ± SEM. Student's *t*‐test, unpaired.MMeasurement of P347S and P347S/miR‐181a/b^+/−^ ONL thickness (*N* ≥ 3 eyes/genotype; Data are presented as mean ± SEM. Student's *t*‐test, unpaired).NGraphical representation of the results of OKR analysis by the optokinetic tracking assays reported as cycles/degree. Visual acuity is preserved in P347S/miR‐181a/b^+/−^ animals with respect to P347S (*N* = 4 animals/genotype). Data are presented as mean ± SEM. Student's *t*‐test, unpaired. [Colour figure can be viewed at wileyonlinelibrary.com]

Notably, the beneficial effect of miR‐181a/b downregulation was still detectable at later time points (p90), when we observed statistically significant preservation of b‐wave ERG response (Fig 3A–C), with amelioration of PR markers expression (Fig 3D–L) and a slight increase in ONL thickness (Fig 3M). We observed a statistically significant increase in photopic a‐waves amplitudes suggesting that the PRs with preserved functionality are mostly cones at this late time point. We also decided to test the spatial frequency threshold of vision (i.e., visual acuity expressed in cycles/degree), determined by measuring the optokinetic response (OKR). P347S animals displayed a dramatic impairment of OKR response, in agreement with previous reports (Yu *et al*, 2017). Conversely, P347S/miR‐181a/b‐1^+/−^ animals showed a significant amelioration of OKR response at the same stage (Fig 3N).

Taken together, these results indicate that miR‐181a/b downregulation improves retinal function and slows down retina degeneration in P347S mice.

### miR‐181a/b‐1 downregulation ameliorates retinal mitochondrial defects in P347S mice

**Figure 4:**
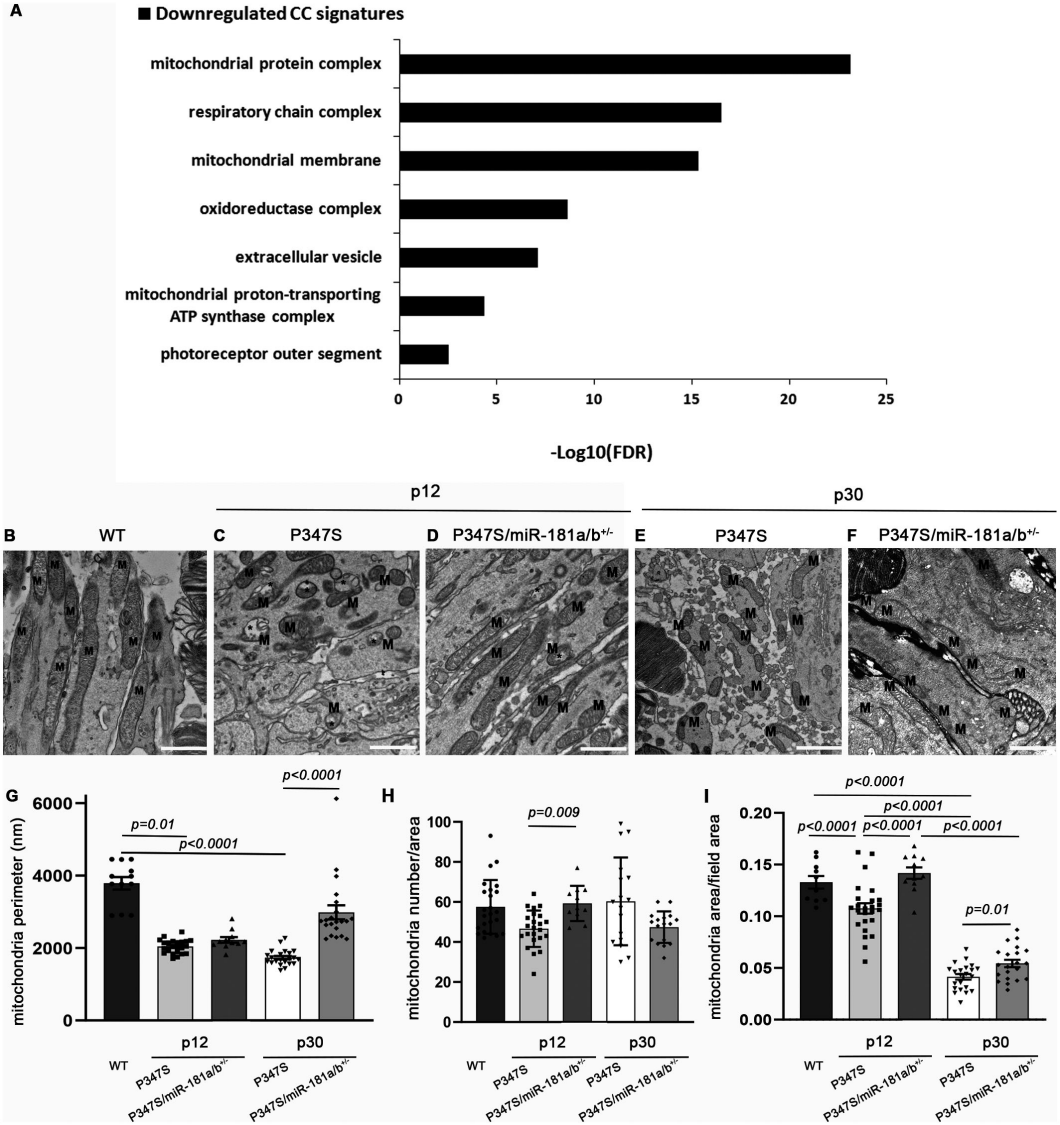
miR‐181a/b‐1 downregulation improves the mitochondrial phenotype in the retina of P347S mice AGene ontology analysis [Cellular component (CC) terms] shows a significant downregulation of mitochondria‐related gene pathways in P347S versus WT retinas at p12, as assessed by transcriptome analysis.B–FElectron microscopy analysis shows an increase of mitochondrial fragmentation in P347S PRs with respect to WT and amelioration of the mitochondrial phenotype in P347S/miR‐181a/b^+/−^ versus P347S PRs at p12 and p30 (*N* = 2 animals/genotype). M, mitochondria.G–IThe quantitative analysis of mitochondrial phenotype is expressed as measurement of the mitochondria perimeter (G), mitochondria number (H) and as the ratio of mitochondria area per the analyzed field area (I). *N* = 2 animals/genotype. Data information: Scale bars are 1 μm. Data are presented as mean ± SD. One‐way ANOVA test.

**Figure EV2:**
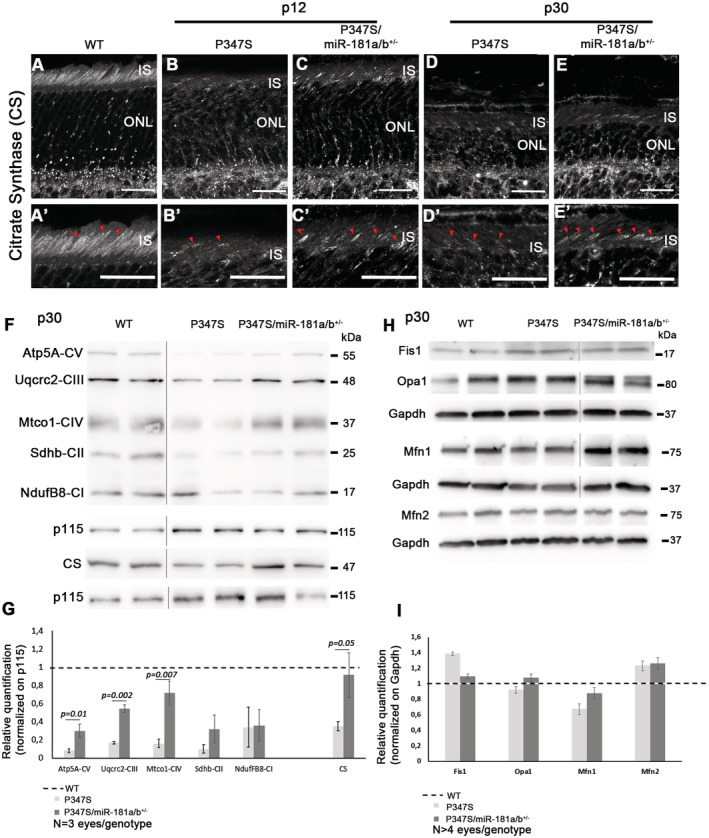
miR‐181a/b‐1 downregulation leads to an increase of mitochondrial proteins A–EImmunofluorescence analysis showed a decrease of Citrate Synthase (CS) staining, a mitochondrial marker, in the OS of P347S retinas versus WT (A) at p12 (B) and p30 (D). The analysis highlights an amelioration of CS staining in P347S/miR‐181a/b^+/−^ versus P347S eyes at both p12 (C) and p30 (E) (Red triangles). (A′–E′) show higher magnification of (A–E). Scale bars 25 μm.F, GWB analysis reveals decreased levels of OXPHOS, representative of Mitochondrial Respiratory Chain complexes, and CS proteins in the eyes of P347S with respect to WT, and partial rescue of these parameters in P347S/miR‐181a/b^+/−^ versus P347S PR at p30 [quantified in (G)]. Data are normalized to p115. *N* = 3 eyes/genotype.H, IWB analysis of key proteins involved in mitochondrial fission/fusion pathway (Fis1 Opa1 and Mfn1/2 proteins) in the eyes of WT, P347S and in P347S/miR‐181a/b^+/−^ at p30 [quantified in (I)]. Data are normalized to Gapdh. *N* ≥ 4 eye/genotype. Data information: Data are presented as mean of Fold Change ± SEM. Student's *t*‐test, unpaired. Please note that all compared bands from WT, P347S and P347S/miR‐181a/b^+/−^ samples are from the same blots, which were cropped and shown organized in the panel for the sake of data presentation clarity (see source data). Source data are available online for this figure.

Mitochondrial dysfunction has been shown to exacerbate PR death and disease progression in mendelian and multifactorial forms of outer retinal degeneration (Lefevere *et al*, 2017; Carrella *et al*, 2021). However, mitochondria defects have not been reported in the P347S model to date. Our previously generated RNA‐seq transcriptome data (Karali *et al*, 2020) revealed a significant downregulation of mitochondria‐related gene pathways at p12 in the P347S retina with respect to wild‐type (WT), indicating the presence of early mitochondrial dysfunction even before the appearance of PR degeneration (Fig 4A, Table EV1). We therefore investigated in further details the putative mitochondrial defects in the P347S retina. EM analysis highlighted a fragmented mitochondrial morphology at p12 and p30 in PRs (Fig 4B, C and E; see Materials and Methods section and Appendix Figs S1 and S2 for further details). The extent of fragmentation was quantified by measuring the mitochondrial perimeter, which is significantly decreased in P347S animals compared with WT mice at both time points (Fig 4G). Although we did not observe any significant variation in the total number of mitochondria (Fig 4H), we found a strong reduction in mitochondrial area in P347S versus WT PRs at p30, suggesting a decrease in mitochondrial mass during retinal degeneration progression (Fig 4I). This observation was further confirmed by the decrease in mitochondrial proteins in P347S retinas as assessed by immunofluorescence of citrate synthase (CS) at p12 and p30 (Fig EV2A–B′, D and D′, red triangles) and by Western blot (WB) for the mitochondrial proteins Atp5A, Uqcrc2, Mtco1, Sdhb, NdufB8, and CS (Fig EV2F and G).

**Figure 5:**
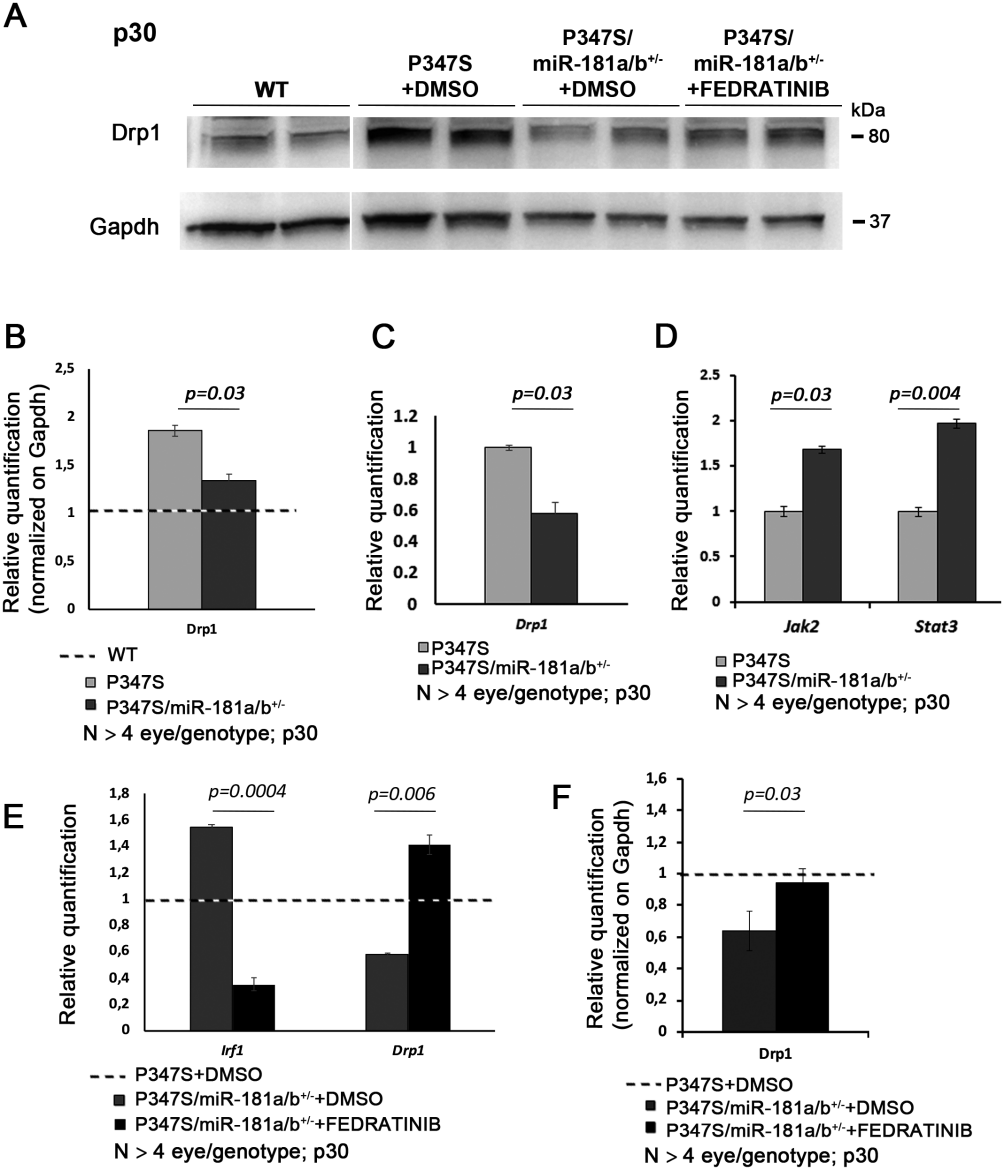
miR‐181a/b‐1 downregulation decreases Drp1 levels via regulation of the Jak2/Stat3 pathway AWB analysis of Drp1, one of the key proteins involved in the mitochondrial fission pathway, in the optic cup of WT, P347S DMSO‐treated, P347S/miR‐181a/b^+/−^ DMSO‐treated and of P347S/miR‐181a/b^+/−^ FEDRATINIB‐treated (an inhibitor of the JAK2/STAT3 pathway) mice at p30.BQuantification revealed that Drp1 protein levels are increased in P347S with respect to WT, and partially rescued in P347S/miR‐181a/b^+/−^. Data are normalized to Gapdh. *N* ≥ 4 eye/genotype. Please note that all compared bands from WT, P347S, P347S/miR‐181a/b^+/−^ and P347S/miR‐181a/b^+/−^ with FEDRATINIB samples are from the same blots, which were cropped and shown organized in the panel for the sake of data presentation clarity (see source data).C, DqRT–PCR analysis reveals decreased levels of the *Drp1* transcript and increased levels of *Stat3* and *Jak2* transcripts in the eyes of P347S/miR‐181a/b^+/−^ versus those of P347S animals. *N* ≥ 4 eyes/genotype.EqRT–PCR analysis on *ex vivo* retinas reveals that the treatment with FEDRATINIB counter‐rescued the upregulation of *Irf1* and the downregulation of *Drp1* transcript levels in P347S/miR‐181a/b^+/−^ animals. *N* ≥ 4 eyes/genotype.FQuantification of WB in A reveals decreased levels of Drp1 in P347S/miR‐181a/b^+/−^ DMSO‐treated versus P347S and increased levels in P347S/miR‐181a/b^+/−^ FEDRATINIB‐treated *ex‐vivo* retinas. *N* ≥ 4 eyes/genotype. Data information: Data are presented as mean of Fold Change ± SEM. Student's *t*‐test, unpaired. Source data are available online for this figure.

**Figure EV3:**
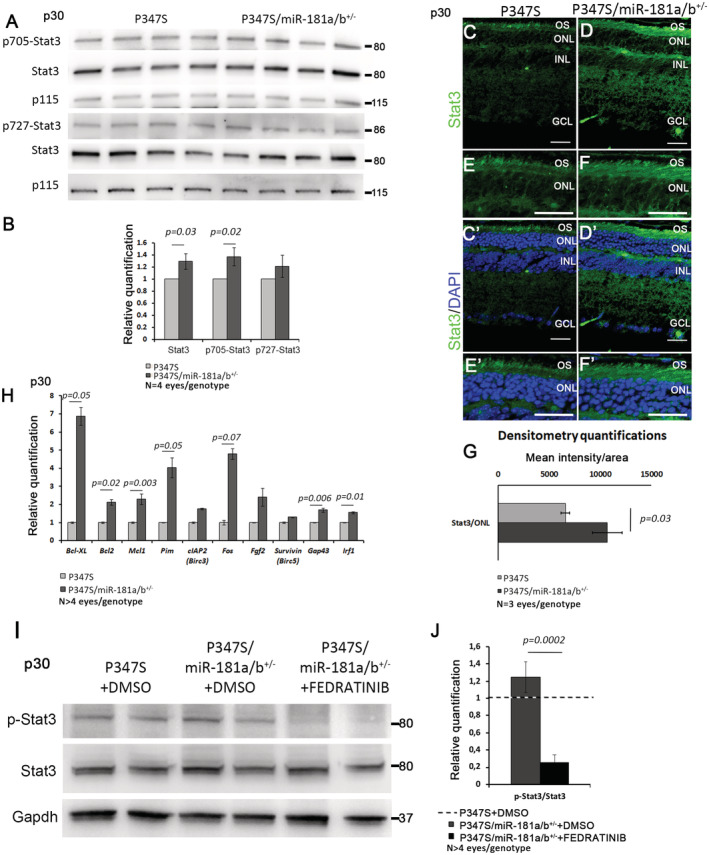
miR‐181a/b‐1 downregulation is associated with an upregulation of the JAK2/STAT3 pathway A, BWB analysis of P347S/miR‐181a/b^+/−^ versus P347S total eye protein extracts at p30 showed increased levels of total Stat3 and p705‐Stat3. No statistically significant variation was observed for p‐727‐Stat3. Data are normalized to p115. Relative protein quantifications are reported in (B). *N* = 4 eyes/genotype. Data are presented as mean of Fold Change ± SEM. Student's *t*‐test.C–GImmunofluorescence analysis of Stat3 in P347S and P347S/miR‐181a/b^+/−^ retinas at p30. (E, F) and (E′, F′) show higher magnification of (C, D) and (C′, D′), respectivey. Scale bars 25 μm. Fluorescence densitometry quantification of Stat3 in the ONL is reported in (G), *N* = 3 eye/genotype for each staining. Data are presented as mean ± SEM. Student's *t*‐test, unpaired.HqRT–PCR analysis reveals increased levels of p705‐Stat3 transcriptional target genes in the eyes of P347S/miR‐181a/b^+/−^ versus P347S animals. *N* ≥ 4 eyes/genotype. Data are presented as mean of Fold Change ± SEM. Student's *t*‐test, unpaired.I, JWB analysis of total Stat3 and p705‐Stat3 in the optic cup of P347S DMSO‐treated, P347S/miR‐181a/b^+/−^ DMSO‐treated and of P347S/miR‐181a/b^+/−^ FEDRATINIB‐treated (an inhibitor of the JAK2/STAT3 pathway) mice at p30 [quantified in (J)] revealed that FEDRATINIB is inhibiting JAK2 activity in the treated samples as showed by the reduction of p705‐Stat3 protein levels in P347S/miR‐181a/b^+/−^ FEDRATINIB‐treated. Data are normalized to Gapdh. *N* ≥ 4 eye/genotype. Data are presented as mean of Fold Change ± SEM. Student's *t*‐test, unpaired. Source data are available online for this figure.

Interestingly, the reduction of miR‐181a/b levels in P347S/miR‐181a/b‐1^+/−^ mice led to an amelioration of CS staining (Fig EV2C, C′, E and E′), an increase in mitochondrial proteins (Fig EV2F and G) and an amelioration of the mitochondrial fragmentation phenotype at p30 (Fig 4D, F and G). Accordingly, WB analysis also revealed significantly increased levels of Drp1 (Fig 5A and B) and a trend of increased levels of Fis1 (Fig EV2H and I), two key regulators of mitochondrial fission, in P347S retinas, while no significant variation was observed for Opa1 and Mfn1/2, which are key regulators of mitochondrial fusion (Fig EV2H and I). We therefore hypothesized that the increase of Drp1 and Fis1 could underlies the mitochondrial fragmentation in P347S PRs. Interestingly, in P347S/miR‐181a/b‐1^+/−^ retinas, we observed that the Drp1 protein levels were decreased with respect to the P347S retina (Fig 5A and B). Notably, downregulation of the Drp1 protein correlates with a decrease in *Drp1* transcript levels in the eyes of P347S/miR‐181a/b‐1^+/−^ with respect to those found in P347S animals (Fig 5C). These data suggest that the partial rescue of Drp1 protein amounts likely occurs at the transcript level. It was recently reported that *Drp1* transcription can be negatively modulated by IRF1 (Huang *et al*, 2020), a downstream effector of the JAK2/STAT3 pathway (Guschin *et al*, 1995; Kojima *et al*, 1996; Yamanaka *et al*, 1996; Sato *et al*, 1997; Garcia‐Diaz *et al*, 2017). We thus hypothesized that the decrease in *Drp1* transcript and protein levels could be mediated by miR‐181a/b targeting of the JAK2/STAT3 pathway. Signal transducers and activators of transcription (STATs) constitute a family of transcription factors that mediate a wide variety of biological functions in the nervous systems. STAT activation is mediated by JAK family members and is induced in response to multiple cytokines and growth factors that are released after injury in neuronal tissues. STAT family members can play different roles. In particular, STAT3 is emerging as a key effector of neuronal survival by inducing the expression of neuroprotective genes (Dziennis & Alkayed, 2008). STAT3 augmentation was previously shown to significantly enhance PR survival and improve retinal electrophysiology in P347S mice (Jiang *et al*, 2014). Both *Jak2* and *Stat3* have been reported to be direct targets of miR‐181a/b (Qu *et al*, 2017; Lai *et al*, 2020). We observed, by qRT–PCR, that *Jak2* and *Stat3* transcripts are upregulated in P347S/miR‐181a/b‐1^+/−^ with respect to P347S eyes (Fig 5D). This upregulation is accompanied by an increase in both STAT3 and of Phospho(p)‐705‐STAT3 protein levels (Fig EV3A and B). Immunofluorescence analysis of the STAT3 protein revealed that the increase in P347S/miR‐181a/b‐1^+/−^ occurs in all retinal layers, including the ONL (Fig EV3C–G). The phosphorylation at position 705 is mainly mediated by JAK2 and leads to the translocation of STAT3 to the nucleus, where it activates the transcription of several pro‐survival genes (Dziennis & Alkayed, 2008; Jiang *et al*, 2014). Consistently, we detected, by qRT–PCR, increased transcript levels of several p‐705‐STAT3 downstream targets, among which *Irf1* (Lee *et al*, 2006; Walch‐Rückheim *et al*, 2016) (Fig EV3H), while we did not observe any statistically significant variation of p‐727‐STAT3 (Fig EV3A and B), a modification that mediates STAT3 translocation to mitochondria (Wegrzyn *et al*, 2009; Zhang *et al*, 2013). To verify whether the upregulation of the JAK2/STAT3 pathway mediates, via *Irf1*, Drp1 downregulation in P347S/miR‐181a/b‐1^+/−^ eyes, we treated P347S/miR‐181a/b‐1^+/−^ eyes with a JAK2 inhibitor (FEDRATINIB) in *ex‐vivo* conditions. Eight hours of treatment led to a decrease of p‐705‐STAT3 as determined by WB (Fig EV3I and J). We observed that the FEDRATINIB treatment dramatically reduces *Irf1* transcript levels (Fig 5E) and increases *Drp1* transcript and Drp1protein levels (Fig 5A, E and F) in P347S/miR‐181a/b‐1^+/−^ as compared with DMSO only treated eyes. These data confirm that downregulation of miR‐181a/b rescues Drp1 levels by enhancing the JAK2/STAT3/IRF1 pathway.

### AAV2/8‐Sponge‐miR‐181a/b delivery downregulates miR‐181a/b activity and ameliorates the retinal phenotype of P347S mice

**Figure EV4:**
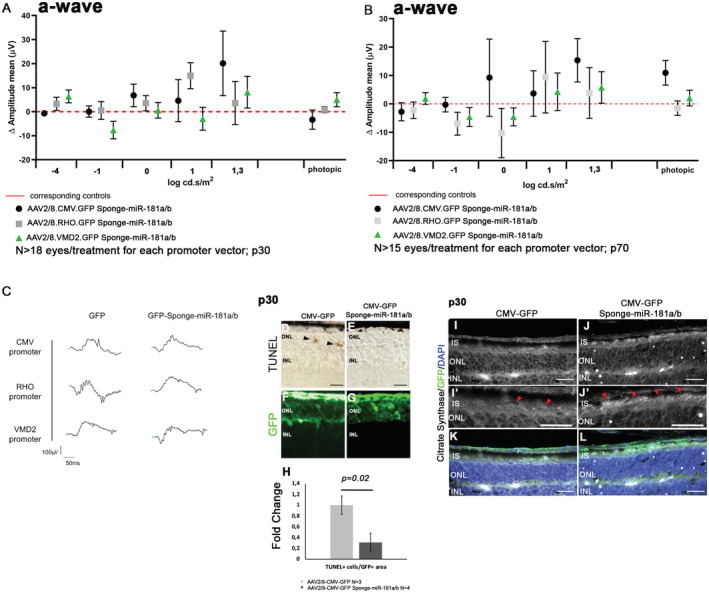
AAV2/8‐Sponge‐miR‐181a/b delivery improves retinal function and slows down degeneration in P347S mice A–CERG response (a‐wave) reported as Delta (Δ) amplitude in P347S animals injected at p4 with AAV2/8.CMV.GFP‐Sponge‐miR‐181a/b, AAV2/8.CMV.RHO‐Sponge‐miR‐181a/b, AAV2/8.VMD2.GFP‐Sponge‐miR‐181a/b vectors with respect to the corresponding controls (AAV2/8.CMV.GFP, AAV2/8.RHO.GFP and AAV2/8.VMD2.GFP; red dotted line) at p30 (A; *N* ≥ 18 eyes/treatment, for each promoter vector) and p70 (B; *N* ≥ 15 eyes/treatment, for each promoter vector). Data are presented as mean of Delta (Δ) amplitude ± SD. Two‐way ANOVA test. P70 representative curves at 20 candles are reported in (C).D–HTUNEL analysis in the ONL of AAV2/8.CMV.GFP (D, F; black triangles *N* = 3) and AAV2/8.CMV.GFP‐Sponge‐miR‐181a/b (E, G; *N* = 4) sub‐retinally‐injected P347S retinas at p30; quantification in (H). Data are presented as mean of Fold Change ± SEM. Student's *t*‐test.I–LImmunofluorescence analysis of Citrate Synthase staining (Red triangles) in AAV2/8.CMV.GFP‐Sponge‐miR‐181a/b (J, J′, L) versus AAV2/8.CMV.GFP (I, I′, K). (I′–J′) show higher magnification of (I–J). Scale bars are 25 μm.

Based on the evidence that the heterozygous genetic inactivation of the miR‐181a/b‐1 locus exerts a beneficial effect in the P347S model (Figs 1, 2, 3, EV1, 4), we decided to further explore the therapeutic potential of miR‐181a/b silencing by using an AAV‐mediated delivery to the retina of a miRNA sponge. MiRNA sponges are RNA molecules with tandemly repeated miRNA antisense sequences (miRNA binding sites, MBS) that can sequester miRNAs from their endogenous targets resulting in their long‐term silencing *in vivo* (Ebert & Sharp, 2010). We previously generated miR‐181a/b sponges and tested their efficacy *in vitro* (Barbato *et al*, 2021). To achieve long‐term loss‐of‐function of miR‐181a/b *in vivo*, we produced AAV2/8 vectors expressing the miR‐181a/b sponge construct (AAV2/8.CMV.GFP‐Sponge‐miR‐181a/b) and a control (AAV2/8.CMV.GFP). To demonstrate efficacy of the miR‐181a/b Sponge in the retina, we injected the AAV2/8.CMV.GFP‐Sponge‐miR‐181a/b vector in the subretinal space of WT mice at p20. As controls, the contralateral eyes were injected with the AAV2/8.CMV.GFP vector. The AAV2/8 serotype efficiently transduces RPE, PRs, and Müller glia cells upon subretinal administration in several species, including mice (Allocca *et al*, 2007). Accordingly, we observed a good transduction of RPE, PRs, and Müller glia cells 3 weeks after subretinal administration of both AAV2/8.CMV.GFP and AAV2/8.CMV.GFP‐Sponge‐miR‐181a/b (Appendix Fig S3A–F). Subretinal injection at p4 of AAV2/8.CMV.GFP‐Sponge‐miR‐181a/b in WT retina does not alter ERG responses at p30 with respect to AAV2/8.CMV.GFP‐injected retinas (Appendix Fig S3G and H). Moreover, we observed, by qRT–PCR, significantly increased levels of miR‐181a/b mRNA targets in AAV2/8.CMV.GFP‐Sponge‐miR‐181a/b versus AAV2/8.CMV.GFP‐injected retinas (Appendix Fig S3I).

**Figure 6:**
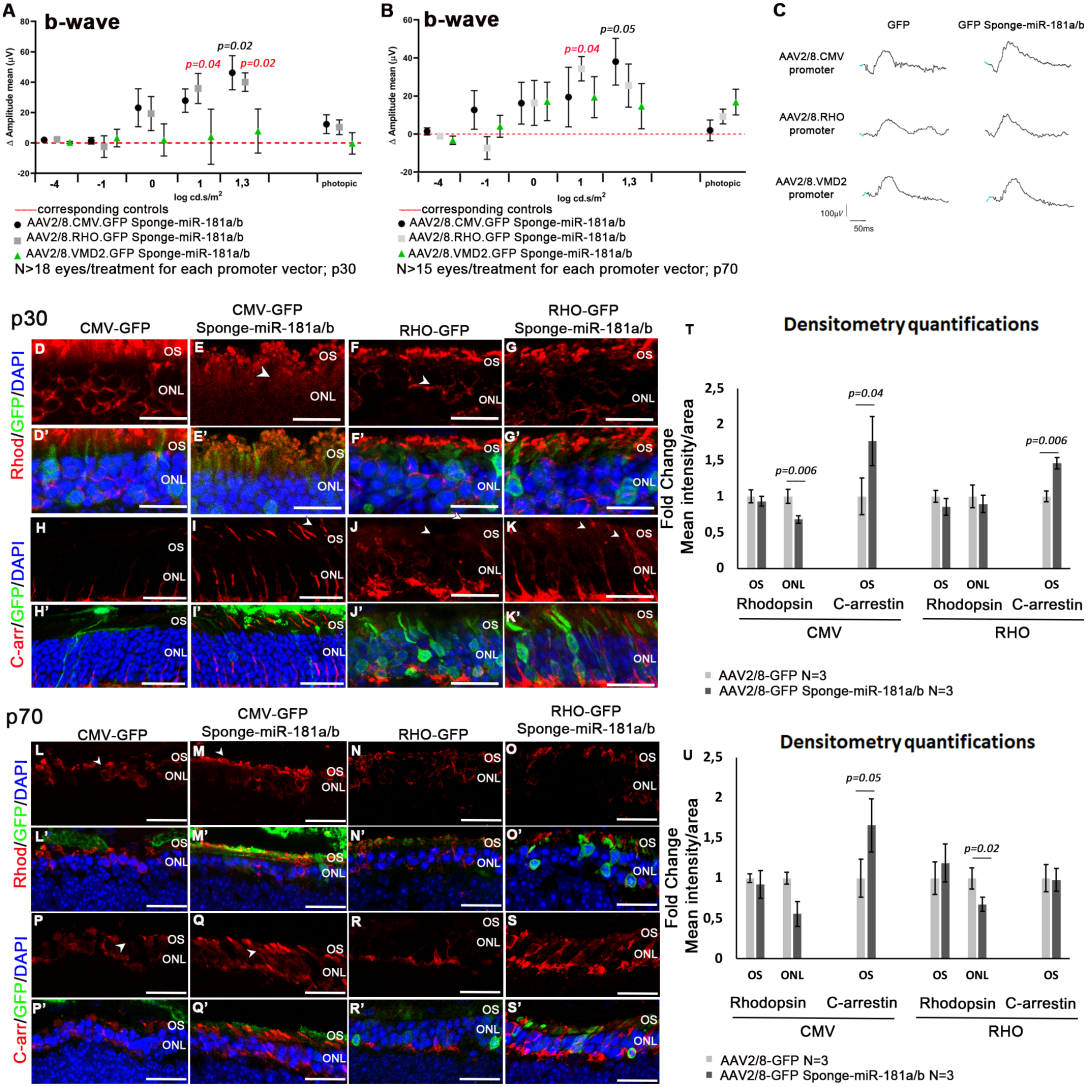
AAV2/8‐Sponge‐miR‐181a/b delivery ameliorates the retinal phenotype of P347S mice A–CERG response (b‐wave) reported as Delta (Δ) amplitude in P347S animals injected at p4 with AAV2/8.CMV.GFP‐Sponge‐miR‐181a/b, AAV2/8.CMV.RHO‐Sponge‐miR‐181a/b, AAV2/8.VMD2.GFP‐Sponge‐miR‐181a/b vectors with respect to the corresponding controls (AAV2/8.CMV.GFP, AAV2/8.RHO.GFP and AAV2/8.VMD2.GFP; red dotted line) at p30 (A; *N* ≥ 18 eyes/treatment, for each promoter vector) and p70 (B; *N* ≥ 15 eyes/treatment, for each promoter vector). *P*‐values of comparison between AAV2/8.CMV.RHO‐Sponge‐miR‐181a/b‐injected eyes and corresponding control (AAV2/8.RHO.GFP) are shown in red, whereas *P*‐values of comparison between AAV2/8.CMV.GFP‐Sponge‐miR‐181a/b‐injected eyes and corresponding controls (AAV2/8.CMV.GFP) are shown in black. Data are presented as mean of Delta (Δ) amplitude ± SD. Two‐way ANOVA test. P30 representative curves at 20 candles are reported in (C).D–UImmunofluorescence analysis showed amelioration of Rhodopsin localization (p30 D–G′, p70 L–O′; white arrowheads) and C‐arrestin (p30 H–K′, p70 P–S′; white arrowheads) expression in Sponge‐miR‐181a/b‐injected eyes versus the corresponding controls also highlighting improvement of the OS structure. At both time points the effect of amelioration was more significant and evident when the AAV2/8.CMV.GFP‐Sponge‐miR‐181a/b was used. Scale bars are 25 μm. Fluorescence densitometry quantification of each staining is reported in T for p30 and in (U) for p70, *N* = 3 eye/treatment/staining. Data are presented as mean of Fold Change ± SEM. Student's *t*‐test, paired. [Colour figure can be viewed at wileyonlinelibrary.com]

We then decided to assess whether the AAV2/8.CMV.GFP‐Sponge‐miR‐181a/b could exert a beneficial effect in IRD mouse models. Toward that goal, we also generated AAV constructs driving Sponge‐miR‐181a/b expression either in the RPE or in the rod PRs using cell type‐specific promoters. To specifically overexpress sponge‐miR‐181a/b in rod PRs, we prepared an expression cassette in which the GFP‐sponge‐miR‐181a/b is under the control of the human *RHO* promoter (Botta *et al*, 2016) (AAV2/8.RHO.GFP‐Sponge‐miR‐181a/b). For the RPE‐specific overexpression, we used the promoter of the human *VMD2* gene (Esumi *et al*, 2004) (AAV2/8.VMD2.GFP‐Sponge‐miR‐181a/b). Vectors driving the expression of GFP under the control of the same promoters (AAV2/8.RHO.GFP and AAV2/8.VMD2.GFP) were used as controls and injected in the contralateral eyes. We tested the above‐described constructs in P347S animals (Appendix Fig S3J–O). The animals were subretinally injected at p4, and their retinas were analyzed at p30 and p70. We observed a significant increase in b‐wave ERG responses, registered under scotopic conditions, at both time points in P347S eyes injected with AAV2/8.CMV.GFP‐Sponge‐miR‐181a/b and in eyes injected with AAV2/8.RHO.GFP‐Sponge‐miR‐181a/b, compared with the corresponding control‐injected eyes (Fig 6A–C). Conversely, we did not observe any significant effect when using the AAV2/8.VMD2.GFP‐Sponge‐miR‐181a/b (Fig 6A–C). These results suggest that miR‐181a/b silencing restricted only to the RPE is not sufficient to exert a protective effect in P347S mice. Although not significant, we observed a trend of amelioration, both at p30 and at p70, in the a‐waves registered in AAV2/8.CMV.GFP‐Sponge‐miR‐181a/b‐injected P347S eyes versus the corresponding controls (Fig EV4A–C). Cryosections of P347S AAV2/8.CMV.GFP‐Sponge‐miR‐181a/b‐ and AAV2/8.RHO.GFP‐Sponge‐miR‐181a/b‐injected retinas were analyzed at p30 and p70. We detected with both constructs, and at both time points, improved PR marker expression, observing greater effect when using the CMV promoter (Fig 6D–G′, L–O′, T and U; please compare panels D and E, white arrowheads). Moreover, we observed amelioration of C‐Arrestin staining in the OS (Fig 6H–K′, P–S′, T and U; white arrowheads) and of Recoverin staining at p70 (Appendix Fig S4A–I). We also evaluated the extent of photoreceptor cell death at p30, by TUNEL assays. We observed a decrease in TUNEL‐positive cells per injected area (GFP‐positive area) in the ONL of AAV2/8.CMV.GFP‐Sponge‐miR‐181a/b‐injected eyes versus those injected with AAV2/8.CMV.GFP (Fig EV4D–H, black triangles). In addition, immunofluorescence analysis showed an amelioration of CS staining in the AAV2/8.CMV.GFP‐Sponge‐miR‐181a/b‐injected IS with respect to the AAV2/8.CMV.GFP‐injected ones (Fig EV4I–L, red triangles), suggesting that Sponge‐miR‐181a/b‐treatment induces an amelioration of the mitochondrial phenotype similar to what we observed in the genetic miR‐181a/b downregulation model (Fig EV2D–E′). To assess whether miR‐181a/b downregulation exerts a beneficial effect also when delivered at later time points, in a fully differentiated retina, we injected P347S mice at p12/p14 using both AAV2/8.CMV.GFP‐Sponge‐miR‐181a/b and AAV2/8.RHO.GFP‐Sponge‐miR‐181a/b along with their corresponding control vectors. Such time points of intervention represent more disease‐relevant stages. Following subretinal injection at p12/p14, we recorded a trend of improved ERG responses (b‐waves) at p30 and p70 (Appendix Fig S5A–C and M–O), with a statistically significant effect at p30 (Appendix Fig S5B), and amelioration of PR markers expression in AAV2/8.CMV.GFP‐Sponge‐miR‐181a/b‐injected eyes versus the corresponding control eyes, more evident at p30 (Appendix Fig S5D–E′, H–I′ and L) than at p70 (Appendix Fig S5P–Q′, T–U′ and X; white arrowheads). A similar trend was also observed in AAV2/8.RHO.GFP‐Sponge‐miR‐181a/b animals subretinally injected at p12/p14 and analyzed at p30 and p70, albeit a statistical significance in ERG results was not obtained (Appendix Fig S5A–C and M–O), and with lower effects on PR marker expression at both p30 and p70 (Appendix Fig S5F–G′, J–L, R–S′ and V–X; white arrowheads).

The above data indicate that AAV2/8.CMV.GFP‐Sponge‐mediated miR‐181a/b inhibition ameliorates the P347S retinal phenotype also when administered after the onset of degeneration. The observation of a stronger effect obtained with AAV2/8.CMV.GFP‐Sponge‐miR‐181a/b versus AAV2/8.RHO.GFP‐Sponge‐miR‐181a/b injections, particularly following injections at later time points, indicates that although the effect of miR‐181a/b downregulation in the P347S model has a PR cell autonomous component, it may probably involve other retinal cell types.

### AAV2/8‐Sponge‐miR‐181a/b delivery ameliorates the retinal phenotype of *rd10* mice

**Figure 7:**
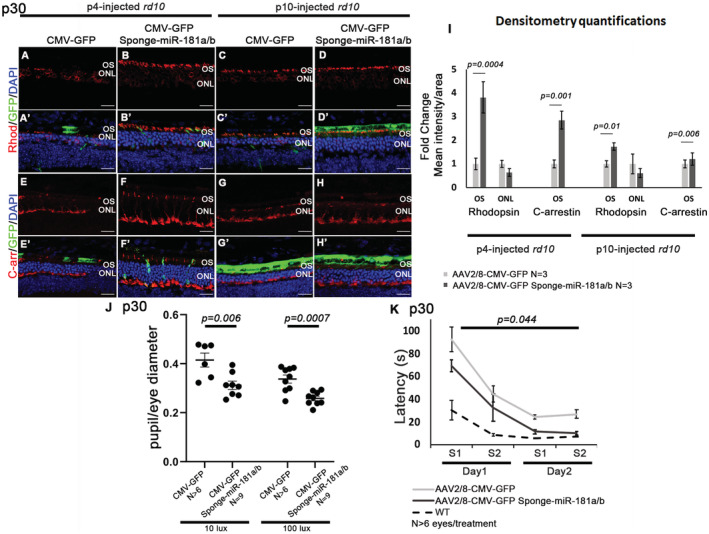
AAV2/8‐Sponge‐miR‐181a/b delivery ameliorates the retinal phenotype of *rd10* mice A–IImmunofluorescence analysis on central retinal sections at p30 showed ONL thickness increase, amelioration of Rhodopsin localization (A–D′) and C‐arrestin expression (E–H′) in AAV2/8.CMV.GFP‐Sponge‐miR‐181a/b‐injected versus AAV2/8.CMV.GFP‐injected *rd10* eyes. Scale bars are 25 μm. Fluorescence densitometry quantification of each staining is reported in (I), *N* = 3 eye/treatment/staining. Data are presented as mean of Fold Change ± SEM. Student's *t*‐test, paired.J, KAnalysis of retinal function based on pupillary light responses (PLR) Water Maze tests. (J) PLR showed a significantly higher pupil constriction in *rd10* mice injected with AAV2/8.CMV.GFP‐Sponge‐miR‐181a/b than in AAV2/8.CMV.GFP‐injected *rd10* eyes, at both 10 lux and 100 lux intensity stimuli (*N* ≥ 6 eyes/treatment and *N* = 9 eyes/treatment, respectively). Data are presented as mean ± SEM. Student's *t*‐test, unpaired. (K) Water maze test highlighted a shorter latency in reaching the platform in AAV2/8.CMV.GFP‐Sponge‐miR‐181a/b‐injected animals respect to control‐injected ones (*N* ≥ 6 eyes/treatment). Data are presented as mean ± SD. ANOVA test. S1 is for Session1 and S2 is for Session2. [Colour figure can be viewed at wileyonlinelibrary.com]

**Figure EV5:**
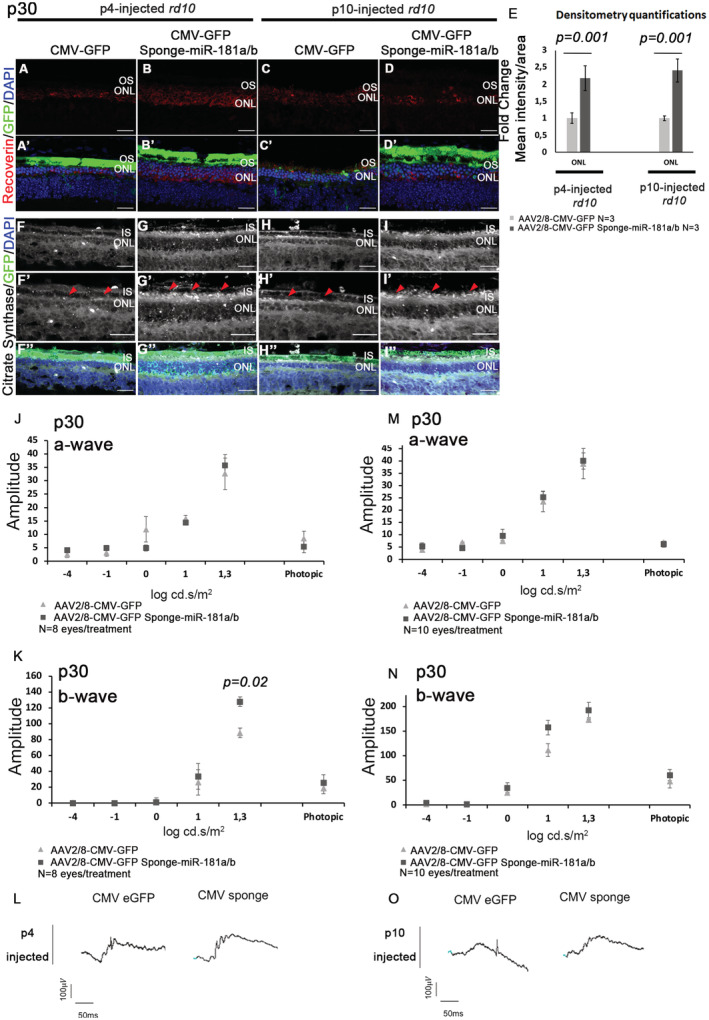
AAV2/8‐Sponge‐miR‐181a/b delivery ameliorates the retinal phenotype of *rd10* mice A–EImmunofluorescence analysis of Recoverin at p30 in p4‐injected‐ (A–B′) and in p10‐injected‐*rd10* (C–D′) with AAV2/8.CMV.GFP‐Sponge‐miR‐181a/b (B, B′ and D, D′) and the corresponding control (AAV2/8.CMV.GFP; A, A′ and C, C′). Scale bars are 25 μm. Fluorescence densitometry quantification of Recoverin staining is reported in (E), *N* = 3 eye/treatment for each staining. Data are presented as mean of Fold Change ± SEM. Student's *t*‐test.F–I″Immunofluorescence analysis of Citrate Synthase (Red triangles) at p30 in p4‐injected‐ (F–G″) and in p10‐injected‐*rd10* (H–I″) with AAV2/8.CMV.GFP‐Sponge‐miR‐181a/b (G–G″ and I–I″) the corresponding control (AAV2/8.CMV.GFP F–F″ and H–H″). (F′–H′) show higher magnification of (F–I). Scale bars are 25 μm.J–LERG response in *rd10* animals injected at p4 [a‐wave in (J) and b‐wave in (K)] with AAV2/8.CMV.GFP‐Sponge‐miR‐181a/b with respect to the corresponding control vectors (AAV2/8.CMV.GFP) at p30 (*N* = 8 eyes/treatment). Data are presented as mean ± SD. Two‐way ANOVA test. Representative curves at 20 candles are reported in (L).M–OERG response in *rd10* animals injected at p10 [a‐wave in (M) and b‐wave in (N)] with AAV2/8.CMV.GFP‐Sponge‐miR‐181a/b with respect to the corresponding control vectors (AAV2/8.CMV.GFP) at p30 (*N* = 10 eyes/treatment). Data are presented as mean ± SD. Two‐way ANOVA test. Representative curves at 20 candles are reported in (O).

Based on the evidence that AAV2/8‐Sponge‐miR‐181a/b delivery exerted a beneficial effect in the retina of P347S mice, we decided to investigate whether this strategy could be extended to other IRD models to evaluate the mutation(gene)‐independent value of this approach. To achieve this goal, we tested the AAV2/8.CMV.GFP‐Sponge‐miR‐181a/b, which yielded the most evident effect in P347S mice, in the *rd10* mouse model. The latter is a model for autosomal recessive (AR) RP and carries a spontaneous mutation in the *Pde6b* gene that recapitulates the human condition (Chang *et al*, 2002). *Pde6b* encodes the β subunit of the rod‐specific phosphodiesterase (βPDE), which, when absent, results in toxic levels of intracellular Ca^2+^ and PR cell death. We chose the *rd10* model because it was previously shown that oxidative stress plays a significant role in its retinal degeneration phenotype, as supported by studies showing that the use of anti‐oxidants is effective in slowing down retinal cell death (Komeima *et al*, 2007, 2008; Oveson *et al*, 2011; Piano *et al*, 2019). Moreover, *rd10* animals display, similar to P347S mice (Jiang *et al*, 2014), increased levels of the STAT3 protein in the degenerating retina (Ly *et al*, 2016; Roche *et al*, 2018). *Rd10* mice show a severely impaired ERG response and a dramatically reduced ONL thickness at p30 (Chang *et al*, 2002). The animals were subretinally injected with AAV2/8.CMV.GFP‐Sponge‐miR‐181a/b in one eye and the corresponding control vector (AAV2/8.CMV.GFP) in the contralateral eye, at p4 or p10. As expected, the ONL thickness of *rd10* AAV2/8.CMV.GFP‐injected retinas was significantly reduced at p30, because of progressive retinal degeneration. Conversely, the ONL thickness was higher at p30 in Sponge‐miR‐181a/b‐injected *rd10* retinas following both injection time points (Fig 7A–H′). The p4‐injected retinas also showed enhanced expression of Rhodopsin (Fig 7A–B′ and I), C‐Arrestin (Fig 7E–F′ and I), and Recoverin (Fig EV5A–E). Moreover, immunofluorescence analysis showed an amelioration of CS staining in AAV2/8.CMV.GFP‐Sponge‐miR‐181a/b‐injected eyes versus AAV2/8.CMV.GFP‐injected controls (Fig EV5F–I″, red triangles). The *rd10* mouse model shows a rapid and severe degeneration (Chang *et al*, 2002); therefore, ERG responses were severely impaired (Fig EV5J–O), as expected, and we could observe only a significant amelioration of b‐wave responses in AAV2/8.CMV.GFP‐Sponge‐miR‐181a/b p4‐injected retinas (Fig EV5K). We thus decided to carry out visual functional assays that allow to detect slight differences in retinal function, such as pupillary light responses (PLR) and water maze tests. Retinal function tests based on PLR showed a significantly higher pupil constriction in AAV2/8.CMV.GFP‐Sponge‐miR‐181a/b‐injected than in AAV2/8.CMV.GFP‐injected *rd10* eyes, at both 10 and 100 lux stimuli (about 23 and 25% more, respectively) (Fig 7J). In addition, the water maze test was used to analyze visual acuity as previously reported (Pang *et al*, 2008; Batabyal *et al*, 2021). This latter test revealed an improved visual function in AAV2/8.CMV.GFP‐Sponge‐miR‐181a/b‐injected animals that showed a significant reduction in the latency to reach the platform with respect to control‐injected ones (Fig 7K). These data indicate that miR‐181a/b downregulation ameliorates retinal morphology and function across different IRD models.

## Discussion

In this report, we show for the first time that miR‐181a/b silencing, obtained both by genetic approaches and by administration to the retina of a specific miR‐181 inhibitor (sponge), exerts a beneficial effect in two very different models of IRDs, namely an autosomal dominant model, characterized by a gain‐of‐function mechanism (P347S) and an autosomal recessive model, caused by a loss‐of‐function mutation (*rd10*). The AAV‐mediated subretinal administration of a specific miR‐181a/b silencing molecule provided us with the proof of the translational potential of miR‐181a/b downregulation as a mutation‐independent therapeutic strategy for IRDs. Beneficial effects were obtained by interventions at early and later time points, when fully differentiated P347S and *rd10* retinas already exhibit initial signs of degeneration. We observed amelioration of visual function and PR marker expression following injection at both time points, although injections at later time point yielded a milder improvement. Nevertheless, the functional recovery by injections carried out after disease onset is important, as it supports the clinical potential of the approach at stages of disease progression relevant for the amelioration of patient's quality of life. The used delivery route, vehicle (i.e., AAV vectors, serotype 2/8), and disease stage at intervention (i.e., onset of retinal degeneration) are compatible with the development of a clinical protocol for human translational purposes (e.g., subretinal delivery in patients) (Hudry & Vandenberghe, 2019). We previously demonstrated that the downregulation of miR‐181a/b, via genetic ablation of cluster‐1, does not impact on retina structure and function (Indrieri *et al*, 2019), and we now showed that the AAV subretinal delivery of the sponge, which results in effective decrease in miR‐181a/b activity, does not alter ERG response providing further support to the safety of this approach.

The data obtained with the use of the *RHO* and *VMD2* promoters demonstrate that the neuroprotective effect of miR‐181a/b downregulation in this IRD model is exerted, at least in part, through a PR‐autonomous mechanism. The AAV2/8.VMD2.GFP‐Sponge‐miR‐181a/b injections did not produce any significant improvement of ERG responses, indicating that the miR‐181a/b downregulation restricted to the RPE is not sufficient to warrant a beneficial effect in the retina of P347S animals. However, we cannot rule out the possibility that the *VMD2* promoter does not yield the same level of expression as the CMV protomer in the RPE, and further experiments are needed to discriminate between the two possibilities. AAV2/8.RHO.GFP‐Sponge‐miR‐181a/b injections determined an amelioration of the retinal phenotype, which, however, was less evident and significant compared with that obtained after AAV2/8.CMV.GFP‐Sponge‐miR‐181a/b injections. This indicates that the administration of the Sponge‐miR‐181a/b under a ubiquitous promoter had a stronger effect, presumably through the pleiotropic action of this miRNA on multiple cell targets that are transduced by the 2/8 AAV serotype (e.g., rod and cone photoreceptors, Müller glia, and microglia). Besides neurons, the retina also contains a glial component, represented by Müller cells, astrocytes, and the microglia, that orchestrate neuroinflammatory response, recovery from injury and progression of disease. At present, we do not have any evidence for a role of miR‐181a/b in microglia modulation, but further studies are needed to gain insight into this issue.

The hypothesis of a PR‐autonomous action of miR‐181a/b, although partial, is also corroborated by our EM data, which revealed an alteration of mitochondria morphology in P347S PRs. Although it was previously reported that the *RHO‐*P347S mutation causes mis‐trafficking of RHO proteins (Li *et al*, 1996; Greenwald *et al*, 2013), the pathogenic mechanisms underlying the retinal degeneration, as well as the final cell death mechanisms, are not fully elucidated in this model. Here, we describe, for the first time, a mitochondrial phenotype in P347S mice that can be detected as early as p12, thus supporting its early involvement in disease pathogenesis. This model displays (i) transcriptome changes pointing to mitochondrial dysfunction and (ii) mitochondrial fragmentation. The latter results correlate with an increase in Drp1 and Fis1 protein levels. These two proteins are key regulators of mitochondrial fission, and therefore, their upregulation may explain the mitochondrial fragmentation phenotype. MiR‐181a/b have already been demonstrated to regulate key genes involved in mitochondrial biogenesis and function (Indrieri *et al*, 2019; Barbato *et al*, 2021). Here, we show that their downregulation rescues the mitochondrial fragmentation phenotype of P347S PRs, with a concomitant amelioration of mitochondrial proteins and a decrease in Drp1 protein levels, mediated by miR‐181a/b regulation of the JAK2/STAT3 pathway. The activation of the latter pathway leads to the increase in several target genes, involved in pro‐survival function. Interestingly, it was previously demonstrated that endogenous Stat3 expression was significantly increased in mutant PRs from three unrelated IRD models [P347S, Prph2^rds/+^ (Jiang *et al*, 2014), and *rd10* mice (Ly *et al*, 2016; Roche *et al*, 2018)], and significantly improves mutant PR survival and retinal electrophysiology (Jiang *et al*, 2014). Although the mechanism underlying the protective effect of Stat3 in IRDs remains to be defined (Jiang *et al*, 2014), it is tempting to speculate that the p705‐Stat3 downstream genes, which are upregulated in our model after miR‐181a/b‐1 inactivation (Fig EV3H), may be responsible for the Stat3‐dependent effect on mutant PR survival and could have an impact on the decrease in cell death detected.

We report here that the activation of the JAK2/STAT3 pathway has an impact on the mitochondrial dynamics related protein Drp1, whose imbalance is often associated with neuronal cell death (Kim *et al*, 2015; Viringipurampeer *et al*, 2016; Oliver & Reddy, 2019). In cells, the equilibrium between small‐fragmented mitochondria and long interconnected mitochondrial networks is essential to ensure mitochondrial homeostasis and cell survival. This equilibrium is maintained by the proper balance between mitochondrial fusion and fission processes, which guarantee the ever‐changing dynamic state of mitochondria, where mitochondrial networks are constantly elongating and dividing (Twig & Shirihai, 2011; Calo *et al*, 2013; Balog *et al*, 2016; Anzell *et al*, 2018). Increased mitochondrial fission and reduced fusion are prominent features in aging and in many neurological diseases (Oliver & Reddy, 2019). The master mediator of fission is Drp1, whose role is fundamental in maintaining balance in mitochondrial dynamics, primarily as a mitochondrial fission factor, but when downregulated, indirectly promotes fusion. In this respect, we observed mitochondria elongation rescue and decreased level of Drp1 in the P347S/miR‐181a/b‐1^+/−^ retina, mediated by the increase in the JAK2/STAT3/IRF1 pathway, thus unveiling a novel mechanism of JAK2/STAT3 regulation of mitochondrial dynamics.

We previously demonstrated that miR‐181a/b control mitochondrial turnover by acting on mitochondrial biogenesis, mitophagy in the retina of mouse models of LHON (Indrieri *et al*, 2019). We now report that these miRNAs also act on mitochondrial fission/fusion balance as revealed by the analysis of an autosomal dominant mouse model of RP. Altogether these findings reveal that miR‐181a/b have a pervasive role in the modulation of mitochondrial activities. Moreover, our results highlight that miR‐181a/b downregulation/silencing has a protective effect in clinically and genetically different types of retinal neurodegenerative diseases, including a mitochondrial disease model (Indrieri *et al*, 2019), an autosomal dominant and an autosomal recessive IRD models. These data support the potential of miR‐181a/b modulation as a gene/mutation‐independent therapeutic approach to slow down the progression of a vast class of retinal diseases with either primary or secondary mitochondrial involvement, which affect both the outer and the inner retina. This strategy can represent a therapeutic agent that dampens disease‐amplifying processes, thus also supporting gene‐specific replacement approaches, and may also be relevant for nonmendelian, multifactorial forms of retinopathies associated with mitochondrial dysfunction, such as age‐related macular degeneration or diabetic retinopathy, although further research efforts are needed to address this latter perspective.

## Materials and Methods

### Animal studies

All studies on mice were conducted in accordance with the institutional guidelines for animal research (ARRIVE guidelines) and approved by the Italian Ministry of Health; Department of Public Health, Animal Health, Nutrition and Food Safety in accordance with the law on animal experimentation (article 7; D.L. 116/92; protocol number: 254/2018‐PR). All animal treatments were reviewed and approved in advance by the Institutional Ethics Committee at the Telethon Institute of Genetics and Medicine (Pozzuoli, Italy). Mice were maintained under specific pathogen‐free (SPF)‐like conditions at the TIGEM Animal Facility. Examinations are conducted periodically on the animals stored in the establishment to confirm they are not contaminated with pathogens of infectious diseases. Same sex litter mates were housed together in individually ventilated cages (IVCs) with maximum four mice per cage. All mice were maintained on a regular 12/12‐h light/dark cycle, temperature of 20–24°C, and humidity of 54–65%, with *ad libitum* access to food and water. Feeding control and sanitary control are carried out under the supervision of a committee that include veterinarians in the establishment. All materials, including IVCs, lids, feeders, bottles, bedding, and water, were autoclaved before use. Both male and female animals were used in all experiments. For subretinal injections in the *RHO‐*P347S background, pups were obtained by crossing the *RHO‐*P347S^+/+^ transgenic (A1 line) (Li *et al*, 1996) with C57BL/6J mice (JAX® Mice Strain, Charles Rivers Laboratories, Strain Code 632), to obtain the P347S^+/−^ mice (Mussolino *et al*, 2011; Botta *et al*, 2016, 2017; Karali *et al*, 2020). Please note that we excluded the presence of the *rd8* allele in the above mice. The *RHO‐*P347S^+/+^ (A1 line) is in the 129 S2/SvHsd (Harlan, Huntington, UK) background and was obtained from the laboratory of Dr. Jane G. Farrar (Loscher *et al*, 2007; Chadderton *et al*, 2009; Palfi *et al*, 2016), and was maintained by crossing A1 P347S^+/+^ with A1 P347S^+/+^. For the analysis of miR‐181a/b genetic decrease in *RHO‐*P347S background, we crossed RHO‐P347S^+/+^ transgenic (Li *et al*, 1996) with miR‐181a/b‐1^+/−^ (Henao‐Mejia *et al*, 2013; Indrieri *et al*, 2019), obtaining in the same litters P347S^+/−^/miR‐181a/b‐1^+/+^, simply termed P347S, and P347S^+/−^/miR‐181a/b‐1^+/−^ animals, termed P347S/miR‐181a/b‐1^+/−^. Please note that all the analyzed P347S and P347S/miR‐181a/b‐1^+/−^ animals were obtained from the same litters and sacrificed at the same time. Pde6b^rd10^ (*rd10*) mice were kindly provided by Dr. Mathias Seeliger (Division of Ocular Neurodegeneration, Centre for Ophthalmology, Institute for Ophthalmic Research).

### RNA extractions

Optic cup samples were processed in QIAzol Lysis Reagent (QIAGEN). Total RNA from optic cup samples was extracted using the RNeasy extraction kit (QIAGEN), according to the manufacturer's instructions.

### Quantitative real‐time PCR

For quantitative real‐time reverse transcriptase PCR (qRT–PCR) experiments, cDNAs were generated using QuantiTect Reverse Transcription Kit (QIAGEN), according to the manufacturer's instructions.

Primers for qRT–PCR reactions were designed to span two different exons to avoid genomic DNA amplification using *in silico* tools (www.basic.northwestern.edu/biotools/oligocalc.html) to predict their melting temperature (*T*_m_) and to avoid the possibility of self‐annealing or primer dimerization. The specificity of the designed primers was tested *in silico* using the BLAT or BLAST tool in Genome Browser (https://genome.ucsc.edu/) or Ensembl (http://www.ensembl.org/index.html). Primers were tested as described (Bustin *et al*, 2009). Primer sequences are reported in Appendix Table S1. Quantification data, obtained in qRT–PCR reactions on cDNAs obtained from different treatments, are expressed in terms of cycle thresholds (Ct). The *HPRT* and *GAPDH* genes were used as endogenous reference controls for experiments. The Ct values were averaged for each in‐plate technical triplicate. The averaged Ct was normalized as difference in Ct values (ΔCt) between the analyzed mRNAs and each reference gene in each sample analyzed. The ΔCt values of each sample were then normalized with respect to the ΔCt values of the control (ΔΔCt). The variation was reported as fold change (2^−ΔΔCt^). Each plate was performed in duplicate, and all the results are shown as means ± SEM of at least three independent biological assays.

### Protein isolation and Western blotting (WB)

Optic cup samples for total protein extraction were homogenized in RIPA buffer with protease inhibitor cocktail 100× (Sigma) and phosSTOP‐Easypack phosphatase inhibitor cocktail tablet (Roche). Protein extract concentrations were determined using the Bio‐Rad protein assay (Bio‐Rad). A total of 30 μg protein from each sample was loaded in SDS–polyacrylamide gels. For WB, gels were electroblotted onto PVDF filters (Millipore) and sequentially immunostained with the following primary antibodies: anti‐Atp5A, anti‐Uqcrc2, anti‐Mtco1, anti‐Sdhb, anti‐NdufB8 (Abcam, Total OXPHOS Rodent WB Antibody Cocktail ab110413, 1:250); anti‐Drp1 (Abcam, ab184247; 1:1,000); anti‐TTC11/Fis1 (Abcam, ab229969; 1:1,000); anti‐Opa1 (BD Biosciences, 612607; 1:1,000); anti‐Mfn1 (Abcam, ab57602; 1:200); anti‐Mfn2 (Abcam, ab56889, 1:1,000); anti‐Citrate Synthase (Abcam, ab9660, 1:1,000); anti‐Stat3 (Cell Signaling, ab30835, 1:1,000); anti‐p507‐Stat3 (Cell signaling, ab4113, 1:1,000); anti‐p727‐Stat3 (Cell Signaling, ab9134, 1:1,000); anti‐p115 (Santa Cruz, sc‐48363, 1:3,000); anti‐Gapdh (Santa Cruz sc‐32233, 1:3,000). Proteins of interest were detected with horseradish peroxidase‐conjugated goat anti‐mouse or anti‐rabbit IgG antibody (1:3,000, GE Healthcare) visualized with the Luminata Crescendo substrate (Millipore) or the Super Signal West Femto substrate (Thermo Scientific), according to the manufacturer's protocol. WB images were acquired using the Chemidoc‐lt imaging system (UVP), and band intensity was calculated using the ImageJ software. The signals for each protein staining were quantified and then normalized for Gapdh or p115 in the same sample (internal normalization). These normalized values were then compared with the values in the control sample. Only bands on the same blot were compared. The average of the normalized values from three different biological replicates is reported as the relative fold change.

### TUNEL assay

For TUNEL assay, mouse eyes were fixed in 4% PFA, cryoprotected with 30% sucrose, embedded in OCT, and cryosectioned. TUNEL assay was performed using *In Situ* Cell Death Detection Kit (Roche) as described (Indrieri *et al*, 2013). Only sections in which the optic nerve was contained were analyzed with a Leica DM‐5500 microscope, and TUNEL‐positive cells were manually counted and normalized per section. The TUNEL count was performed on three eyes/genotype on at least six sections/eye that contain the optic nerve. For the analysis of photoreceptor cell death in AAV2/8.CMV.GFP‐Sponge‐miR‐181a/b‐injected eyes (*N* = 4; four sections/eye that contain the optic nerve) and in AAV2/8.CMV.GFP‐injected ones (*N* = 3; four sections/eye that contain the optic nerve), only GFP‐positive areas were taken into account for the count of TUNEL‐positive cells, and the data were normalized per area.

### Immunofluorescence and immunohistochemistry analysis

For immunofluorescence analysis, mouse eyes were fixed in 4% PFA, cryoprotected with 30% sucrose, embedded in OCT, and cryosectioned. Fourteen‐micrometer cryosections was collected on slides (Superfrost Plus; Fisher Scientific, Pittsburgh, PA). Sections were permeabilized by incubation with 1% NP40 for 15 min for Rhodopsin and C‐Arrestin immunostaining, or by boiling in sodium citrate buffer (0.1 M citric acid, 0.1 M sodium citrate in water) for Caspase 3 active staining or in citrate buffer (0.01 M citric acid in water) for citrate synthase and Stat3 staining. After 1‐min boiling, the slides were cooled down at room temperature. The following primary antibodies were incubated overnight: anti‐Rhodopsin (Abcam, ab3267, 1:5,000); anti‐C‐Arrestin (Millipore, 1:1,000); anti‐Recoverin (Sigma‐Aldrich, AB5585, 1:500); anti‐Citrate Synthase (Abcam, ab9660, 1:500); anti‐Stat3 (Cell Signaling, ab30835, 1:100); anti‐Caspase 3 active (R&D System, AF385, 1:1,000). Sections were then incubated with the Alexa Fluor secondary antibodies (1:1,000; Invitrogen). Phalloidin staining was performed washing cryosections three times in PTW1×, blocking for 1 h in PTW1× + 10% FBS and then sections were incubated for 20 min with Phalloidin (Invitrogen Alexa Fluor 568). For PNA staining, cryosections were washed three times with PBS1×. The slides were incubated over night with (FITC)‐conjugated peanut agglutinin (PNA) (L7381, Sigma‐Aldrich). Sections were counterstained with DAPI (Vector Laboratories). All the immunofluorescence stainings were acquired using a Zeiss LSM700 Confocal, with the exception of the Citrate Synthase staining that was acquired through a Zeiss LSM800 Confocal. Only sections in which the optic nerve was contained were used and two images from each section were photographed in comparable regions equidistant from the optic nerve head. The fluorescent images were converted to grayscale and normalized to background staining, using ImageJ. Quantification of each staining reactivity was measured as mean values to define fluorescence signal intensity (IntDen/Area) and as the area occupied by fluorescent‐labeling in each region of interest.

### Active‐caspase 3‐positive cells count

Immunostained sections for Active‐casp3, in which the optic nerve was contained, were analyzed with a Leica DM‐5500 microscope and positive cells were manually counted and normalized per section. The analysis was performed on 4–6 eyes/genotype on at least eight sections/eye that contain the optic nerve.

### ONL thickness and count of nuclei in the ONL

Mouse eyes were fixed in 4% PFA, cryoprotected with 30% sucrose, embedded in OCT and cryosectioned. Fourteen‐micrometer cryosections was collected on slides (Superfrost Plus; Fisher Scientific, Pittsburgh, PA), and washed three times with PTW1×. The sections were then incubated with DAPI in phosphate‐buffered saline (PBS)1× for 10 min. Count of nuclei in ONL was performed on three eyes/genotype (at least two sections per eye) at p30. Only sections in which the optic nerve was contained were used, and two images from each section were photographed under a Leica DM‐5500 microscope, with the objective Leica ∞/0.17/D, HCX PL FLUOTAR, 40×/0.75 that acquires an area of 0.31 mm^2^, in comparable regions equidistant from the optic nerve head (Barbato *et al*, 2017; Karali *et al*, 2020; Ciampi *et al*, 2022; Intartaglia *et al*, 2022). The acquired images were used for nuclei counts. DAPI‐positive nuclei in ONL were count manually using ImageJ program and normalized per area. On the same images, the ONL thickness was manually measured using ImageJ program, by measuring the distance between the Henle's fiber layer (HFL) and the outer limiting membrane (OLM). The same analysis was performed at p90 on *N* ≥ 3 eyes/genotype (at least two sections per eye).

### Electron microscopy, outer segment (OS) length analysis and mitochondrial phenotype analysis

Mice, at p12 and p30, were deeply anesthetized and perfused with 1% glutaraldehyde and 2% PFA in 200 mM Hepes Buffer, pH 7.3 through the heart. Eyes were removed, and under a dissection microscope, the eyeball was cut along the line between the anterior and posterior chambers of the eye using Spring scissors (Fine Science Tools). Lens were removed and the retina and RPE was left for 1 h in the described fixative solution. Specimens of retina+RPE were postfixed in 1% osmium tetroxide, dehydrated, and embedded in epoxy resin. Retina samples were cut on ultramicrotome LEICA EM UC7 and collected on the single slot oval grids and analyzed with FEI electron microscope. Only tissue sections in which the sagittal orientation of the connecting cilium (cc) was visible were chosen, as shown in Appendix Fig S1. We analyzed two eyes/genotype on at least 10 images per eye, for a total number of OS measured of around 200 OS for the P347S and 150 OS for the P347S/miR‐181a/b‐1^+/−^. These data are represented as mean ± SEM (Standard Error of the Mean). The researchers in charge of producing the TEM images were blind to the genotypes of the animals.

The mitochondrial characterization was performed for all mitochondria located within the entire inner segment (IS), from the outer limiting membrane to the outer segment (OS) with nascent disks (see Appendix Fig S2), observing that the changes in shape and size that we report are independent from localization within the IS. We analyzed mitochondrial number, perimeter, and area on several TEM images derived from two eyes/genotypes each time point, for a total number of images analyzed of 22/genotype for each time point of analysis. Mitochondrial number was determined using FEI software. Mitochondrial surface area and perimeter were analyzed on the same images using the iTEM software, and these data are represented normalized per field, as previously reported (Costa *et al*, 2010; Naon *et al*, 2016; Quintana‐Cabrera *et al*, 2018; Indrieri *et al*, 2019; Tsakiri *et al*, 2019; Xia *et al*, 2019; Ozaki *et al*, 2020; Anastasia *et al*, 2021; Mirra *et al*, 2021). The researchers in charge of producing the TEM images were blind to the genotypes of the animals.

### Electroretinogram

Mice were dark reared for 3 h and anesthetized. Flash ERGs were evoked by 10‐ms light flashes generated through a Ganzfeld stimulator (CSO, Costruzione Strumenti Oftalmici, Florence, Italy) and registered as previously described (Botta *et al*, 2016; Indrieri *et al*, 2019) ERGs and b‐wave thresholds were assessed using the following protocol. Eyes were stimulated with light flashes increasing from −5.2 to +1.3 log cd*s/m^2^ (which correspond to 1 × 10^−5.2^ to 20.0 cd*s/m^2^) in scotopic conditions. For ERG analysis in scotopic conditions, the responses evoked by 11 stimuli (from −5.2 to +1.3 log cd*s/m^2^) with an interval of 0.6 log unit were considered. To minimize the noise, three ERG responses were averaged at each 0.6 log unit stimulus from −5.2 to 0.0 log cd*s/m^2^ while one ERG response was considered for higher stimuli (from 0.0 to +1.3 log cd*s/m^2^). a‐ and b‐waves amplitudes recorded in scotopic conditions were plotted as a function of increasing light intensity (from −4 to +1.3 log cd*s/m^2^). The photopic ERG was recorded after the scotopic session by stimulating the eye with ten 10 ms flashes of 20.0 cd*s/m^2^ over a constant background illumination of 50 cd/m^2^.

### Optokinetic tracking

Visual acuity of mice was assessed by using the Optomotor system (OptoMotry; Cerebral Mechanics) as previously described (Indrieri *et al*, 2019). Briefly, the OptoMotry machine (CerebralMechanics Inc.) is constituted by a plexiglass box [39 × 39 × 32.5 cm (L × W × H)] with rectangular openings [33.5 × 26.5 cm (W × H)] on each wall painted flat white on the inside (screens). Inside the box, a platform is positioned 13 cm above the floor by securing a white Plexiglas disk (diameter, 5.3 cm). Above and below the screens, there are two mirrors and a camera at the top that allows one to follow the behavior of the animal. The computer program (OptoMotry; CerebralMechanics, Lethbride, Alberta, Canada) was used to control the rotation speed and the geometry of the stimuli as well as the spatial frequency and contrast of the stimuli. Analyses have been conducted twice, at two different time points (p40 and p90), by increasing the spatial frequency for each direction of rotation (right/left), after an initial trial of 10 min. The maximum spatial frequency that the animal is able to perceive is recorded as a threshold of visual acuity and reported in the graph as cycles/degree on the *y*‐axis.

### Pupillary light response

Pupillary light responses from *rd10* mice were recorded in dark condition using the TRC‐50IX retinal camera connected to a charge‐coupled device NikonD1H digital camera (Topcon Biomedical Systems), as previously described (Tornabene *et al*, 2019). Briefly, mice were exposed to 10 lux light‐stimuli for approximately 10 s and one picture per eye was acquired using the IMAGEnet software (Topcon Biomedical Systems). For each eye, the pupil diameter was normalized to the eye diameter (from temporal to nasal side).

### Water maze test

We used a modified version of the visual water maze task (Marrocco *et al*, 2020) consisting of a circular tank of 1.4‐m diameter gray metal, high 40 cm. A visible black platform was 13 cm high, protruding 1 cm above the water (21 ± 2°C) that was made white with natural color and was positioned in a well‐lit room. The north site the pool contained a divisor panel. Across trials, the platform was randomly located on the right and left side equidistant from the divisor. The animal was always released from the south quadrant, and the latency to reach the platform was recorded by using the video tracking AnyMaze (Stoelting, USA). Black curtains were positioned around the pool to limit distal cues; the access to distal cues was prevented by the wall of the pool. To acclimatize the mice to the platform, the training started with the animal positioned on the platform for 60 s, before the first training session was administered. Training consisted of two sessions per day of six trials for two 2 consecutive days. Every two consecutive trials animals rested for 10 min within sessions. The position of the platform is randomly displaced every two trials. Intersession interval was about. Subjects were given up to 120 s to escape during each trial; if they did not escape within the allotted time, they were gently guided to the platform and their escape time was recorded as this maximum. All mice remained on the platform between trials, then after each block they were towel‐dried, and transferred to their home cages under warm air. Behavioral data were acquired with a latency to reach the platform during the training trials. All measurements were monitored by a video camera.

### FEDRATINIB *ex vivo* retina treatment

P347S and P347S/miR‐181a/b‐1^+/−^ animals were sacrificed by cervical dislocation, following the institution's guidelines, at p30. Eyes were removed quickly using Dumont's forceps #5 and put in CO2‐independent medium—Gibco (18045‐054). Under a dissection microscope, the eyeball was opened and cut along the line between the anterior and posterior chambers of the eye using Spring scissors (Fine Science Tools). Lens were removed and the retina and RPE, attached in the inferior part of the eyeball, was dissected out using #5 forceps and flat‐mounted retina/RPE was placed in a new dish containing Dulbecco's modified eagle medium supplemented with 10% FBS (Euroclone) and 1% penicillin/streptomycin. The *ex vivo* retinas were treated with the JAK2 inhibitor FEDRATINIB (SAR302503, TG101348; Selleckchem) 100 μM for 8 h. Control retinas were treated with DMSO.

### Sponge design, construct, and AAV preparation

We previously generated miR‐181a/b sponge construct (Barbato *et al*, 2021). The sequence is covered by the patent application “miR‐181 inhibitors and uses thereof” (WO2019202162A1). MiR‐181a/b sponge was cloned in a vector, containing an expression cassette, into the 3′ UTR of a green fluorescent protein (GFP) reporter gene, under the control of the cytomegalovirus (CMV) constitutive promoter. To specifically overexpress sponge‐miR‐181a/b in rod PRs, we prepared an expression cassette in which the GFP‐sponge‐miR‐181a/b is under the control of the human *RHO* promoter (Botta *et al*, 2016), and for the RPE‐specific overexpression, we used the promoter of the human *VMD2* gene (Esumi *et al*, 2004). We generated AAV2/8 vectors expressing “sponge” constructs to achieve long‐term loss‐of‐function of miR‐181a/b *in vivo* (AAV2/8.CMV.GFP‐Sponge‐miR‐181a/b; AAV2/8.RHO.GFP‐ Sponge‐miR‐181a/b; AAV2/8.VMD2.GFP‐Sponge‐miR‐181a/b). Vectors, driving the expression of GFP under the control of the same promoters (AAV2/8.CMV.GFP; AAV2/8.RHO.GFP and AAV2/8.VMD2.GFP), were used as controls. Recombinant AAV2/8 viruses were produced by the TIGEM Vector Core as reported (Hildinger *et al*, 2001; Doria *et al*, 2013).

Plasmids used for AAV vector production are as follows: the pAd helper plasmid that contains the adenovirus E2A, E4, and VA RNA helper genes (Zhang *et al*, 2000); the pAAV2/8 (Gao *et al*, 2002) packaging plasmid with AAV rep2 and cap8 genes; the pAAV2.1 plasmid that contains the expression cassette flanked by two identical inverted terminal repeats (ITRs), the ampicillin resistance and the bovine growth hormone polyadenylation signal (bGH) (Auricchio *et al*, 2001).

Adeno‐associated viral vectors were produced by triple transfection of HEK293 cells and were purified by two rounds of CsCl2 gradients as already described (Doria *et al*, 2013). Physical titers [genome copies per milliliter (GC/ml)] of each viral preparation were determined by averaging the titer achieved by dot‐blot analysis and by PCR quantification using TaqMan (Applied Biosystems, Carlsbad, CA) (Doria *et al*, 2013).

### Subretinal injections

Surgical procedures were performed under anesthesia, and all efforts were made to minimize suffering. Viral vectors were delivered subretinally in the dorsal retinal areas via a trans‐scleral transchoroidal approach (Liang *et al*, 2000). Eyes were injected with 1 μl of AAV vector (specified in each figure) containing a total of 1 × 10^13^ viral GCs.

We excluded from our analysis samples that showed sign of injection‐related damages, or eyes that turned out to be not properly injected as assessed from GFP expression analysis. The fluorescence of eye fundi were acquired using a TRC‐50IX retinal camera connected to a charge‐coupled device NikonD1H digital camera (Topcon Biomedical Systems). Retinal sections were also analyzed to determine the GFP expression and images acquired using the TILE‐SCAN function at the Leica DM‐5500 microscope.

### Statistical analysis

Animals were allocated to experimental and control groups randomly based on the appropriate genotype/conditions/treatments. In general, for each experiment, we used ≥ 3 animals per genotype, in order to obtain statistically suitable values. EM analysis, behavioral, and visual tests were carried out blinded. The number of experimental replicates is indicated in each figure legend. The normality assumption was verified using Shapiro–Wilk test. In all experiments, significance of differences between groups was evaluated by one‐tailed or two‐tailed Student's *t*‐test when the comparison was between two experimental groups, one‐way or two‐way ANOVA with *post hoc* Tukey analysis and/or Analysis of Deviance for Generalized Linear Model or Analysis of Deviance for Negative Binomial Generalized Linear Model when the comparison was between more than two experimental groups. *P* ≤ 0.05 was considered significant. Quantitative data are presented as the mean ± SEM (Standard Error of the Mean) of at least three independent experiments, or as the mean ± SD (Standard Deviation) when ANOVA test was applied, of at least three independent experiments. ANOVA test was used to analyze the visual functional data, for example, ERG responses and water maze test, and EM data analysis.

## Author contributions

**Alberto Auricchio:** Resources; supervision; writing – review and editing. **Sandro Banfi:** Conceptualization; resources; supervision; funding acquisition; writing – original draft; project administration; writing – review and editing. **Davide Piccolo:** Investigation. **Elvira De Leonibus:** Data curation; formal analysis; supervision; writing – review and editing. **Enrico Maria Surace:** Resources; supervision; writing – review and editing. **Brunella Franco:** Resources; supervision; writing – review and editing. **Georgios Petrogiannakis:** Investigation. **Alessia Indrieri:** Conceptualization; supervision; writing – review and editing. **Irene Guadagnino:** Formal analysis; investigation. **Jorge Garcia Piqueras:** Investigation. **Ludovica Ciampi:** Investigation. **Martina Di Guida:** Data curation; formal analysis; investigation. **Marta Molinari:** Investigation; methodology. **Elena Marrocco:** Data curation; formal analysis; investigation; methodology. **Mariateresa Pizzo:** Validation; investigation. **Simona Brillante:** Data curation; formal analysis; investigation. **Sabrina Carrella:** Conceptualization; data curation; formal analysis; supervision; investigation; visualization; writing – original draft; project administration; writing – review and editing. **Sara Barbato:** Investigation. **Yulia Ezhova:** Investigation.

## Disclosure and competing interests statement

SC, BF, AI, and SB are coinventors on the patent application number WO2019202162A1. The other authors declare no conflict of interest.

## Supporting information




Appendix

Expanded View Figures PDF

Table EV1

Source Data for Figure 5

Source Data for Expanded View

PDF+

## Data Availability

This study includes no data deposited in external repositories.
