# Supplementary material for: miR‐181a/b downregulation: a mutation‐independent therapeutic approach for inherited retinal diseases
Source: EMBO Mol Med. 2022 Oct 4;14(11):e15941. doi: 10.15252/emmm.202215941 (PMC9641422; doi:10.15252/emmm.202215941)
Supplement: Supplementary file 1 — Appendix [file EMMM-14-e15941-s006.pdf]

Appendix

miR-181a/b downregulation: a mutation-independent therapeutic approach for Inherited Retinal Diseases

Sabrina Carrella, Martina Di Guida, Simona Brillante, Davide Piccolo, Ludovica Ciampi, Irene Guadagnino, Jorge Garcia Piqueras, Mariateresa Pizzo, Elena Marrocco, Marta Molinari, Georgios Petrogiannakis, Sara Barbato, Yulia Ezhova, Alberto Auricchio, Brunella Franco, Elvira De Leonibus, Enrico Maria Surace, Alessia Indrieri, Sandro Banfi

Table of contents

Figure Appendix S1
Figure Appendix S2
Figure Appendix S3
Figure Appendix S4
Figure Appendix S5

Appendix Table S1

Figure Appendix S1

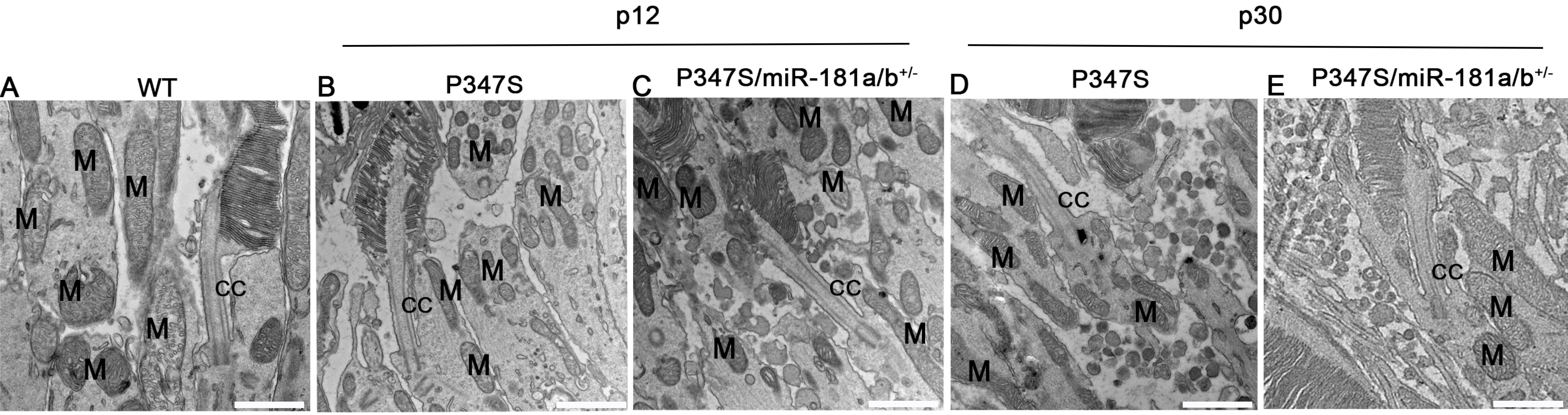

The Electron Microscopy analysis of WT, P347S and P347S/miR-181a/b$^{+/-}$ retinas at p12 and p30 was carried out on images where the sagittal orientation of the connecting cilium (cc) was visible (N≥2 animals/genotype/time point).
M: mitochondria; CC: Connecting Cilium

Figure Appendix S2

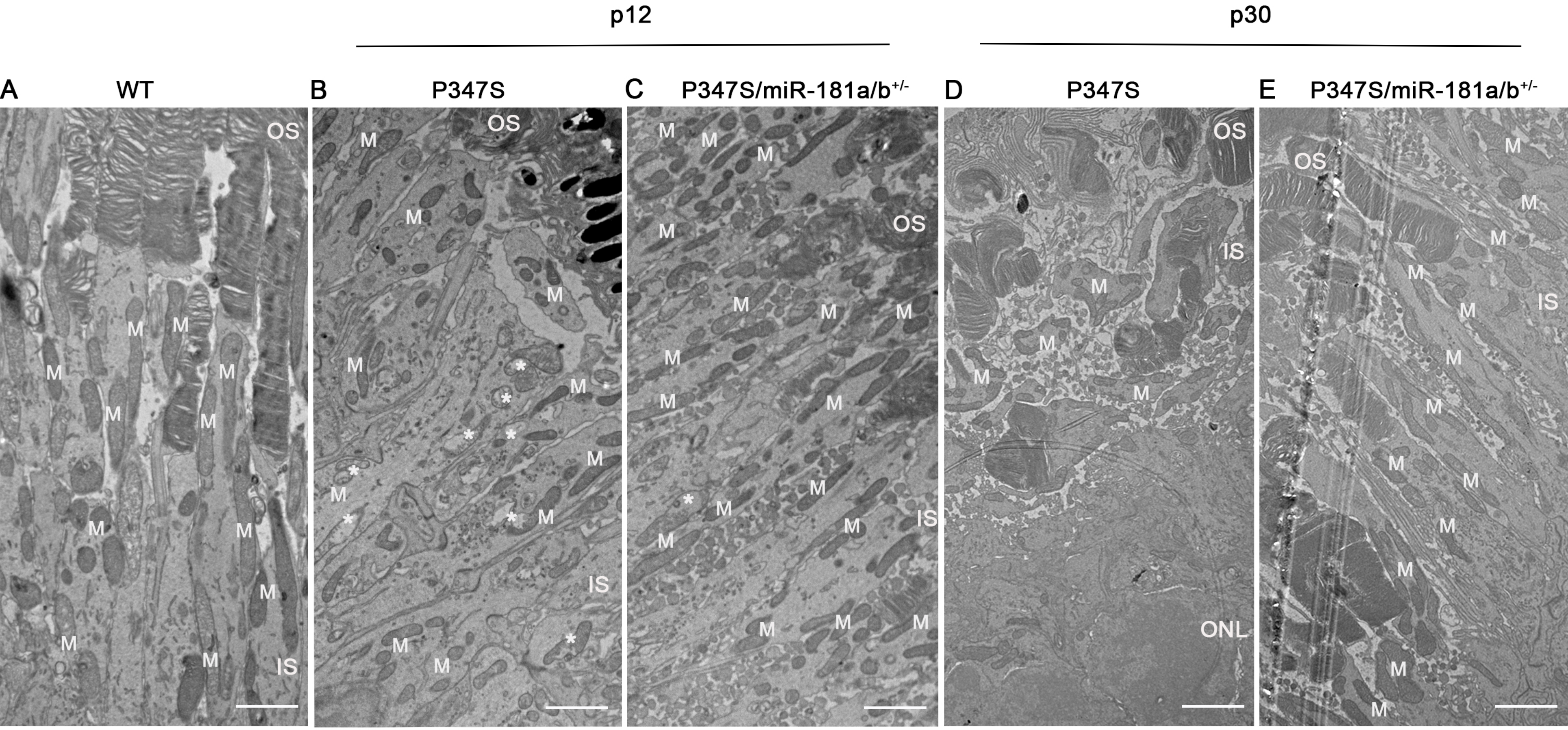

The mitochondrial characterization was performed on Electron Microscopy images of WT, P347S and P347S/miR-181a/b$^{+/-}$ retinas at p12 and p30 for all mitochondria located within the entire inner segment (IS), from the outer limiting membrane to the outer segment (OS) nascent disks.

M: mitochondria; OS: Outer segment; IS: Inner Segment; *: swollen mitochondria

(scale bars are 2 µm)

Figure Appendix S3

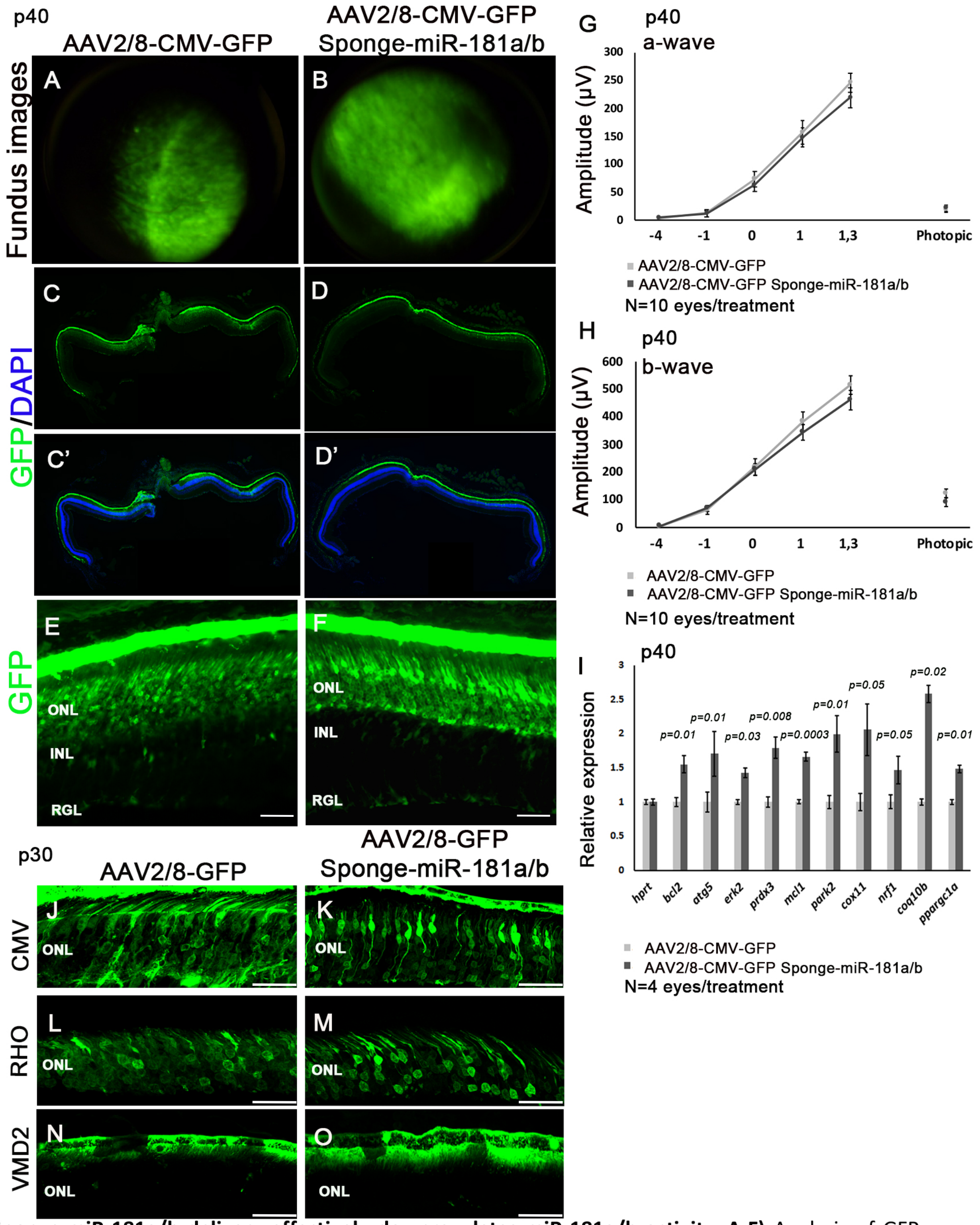

AAV2/8-Sponge-miR-181a/b delivery effectively downregulates miR-181a/b activity. A-F) Analysis of GFP expression in sub-retinally injected eyes. **A, B)** Analysis of eye fundi in AAV2/8.CMV.GFP (A) and AAV2/8.CMV.GFP-Sponge-miR-181a/b (B) sub-retinally-injected eyes. **C-D')** WT retinal sections of AAV2/8.CMV.GFP (C, C') and AAV2/8.CMV.GFP-Sponge-miR-181a/b (D,D') sub-retinally-injected eyes acquired using the TILE-SCAN tool with a Leica DM-6000 microscope. **E, F)** WT retinal sections of AAV2/8.CMV.GFP (E) and AAV2/8.CMV.GFP-Sponge-miR-181a/b (F) sub-retinally-injected eyes acquired using a Zeiss LSM700 confocal microscope. **G, H)** a-wave (G) and b-wave (H) ERG responses at p30 of AAV2/8.CMV.GFP and AAV2/8.CMV.GFP-Sponge-miR-181a/b of WT eyes sub-retinally-injected at p4. N=10 eyes/treatment. Data are presented as mean ± SD. Two-way ANOVA test. **I)** qRT-PCR analysis of miR-181a/b target transcripts in AAV.GFP-Sponge-miR-181a/b- vs AAV2/8.GFP-injected WT eyes. N=4 eyes/treatment. Data are presented as mean of Fold Change ± SEM. Student's t-test, paired. **J-O)** P347S retinal section of AAV2/8.GFP-Sponge-miR-181a/b- vs AAV2/8.GFP-injected eyes under the control of CMV promoter (J, K), RHO promoter (L, M) and VMD2 promoter (N, O). Scale bars are 50 µm

Figure Appendix S4

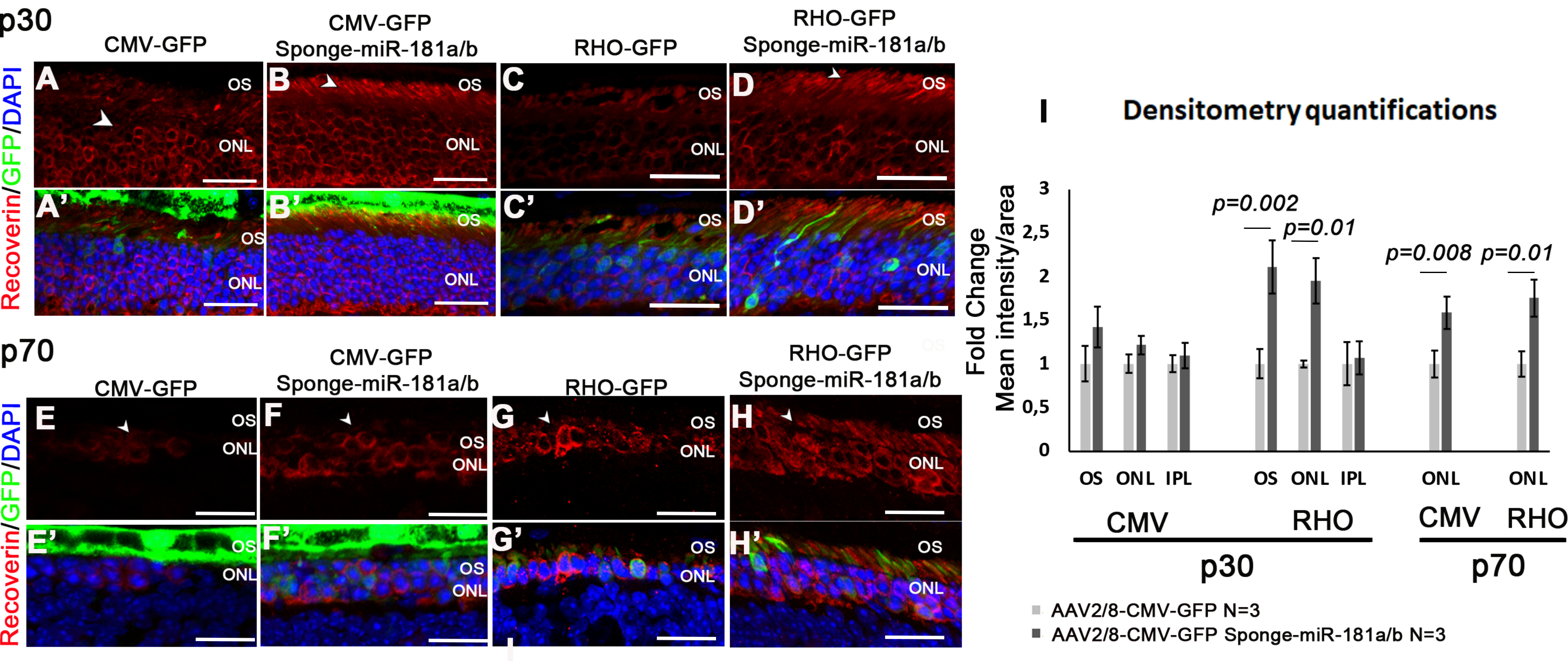

Immunofluorescence analysis of Recoverin staining (p30 A-D', p70 E-H'; white arrowheads) in Sponge-miR-181a/b-injected eyes vs the corresponding controls. Scale bars are 25 μm. Fluorescence densitometry quantification of Recoverin staining is reported in I, N=3 eye/treatment for each staining. Data are presented as mean of Fold Change ± SEM. Student's t-test, paired.

Figure Appendix S5

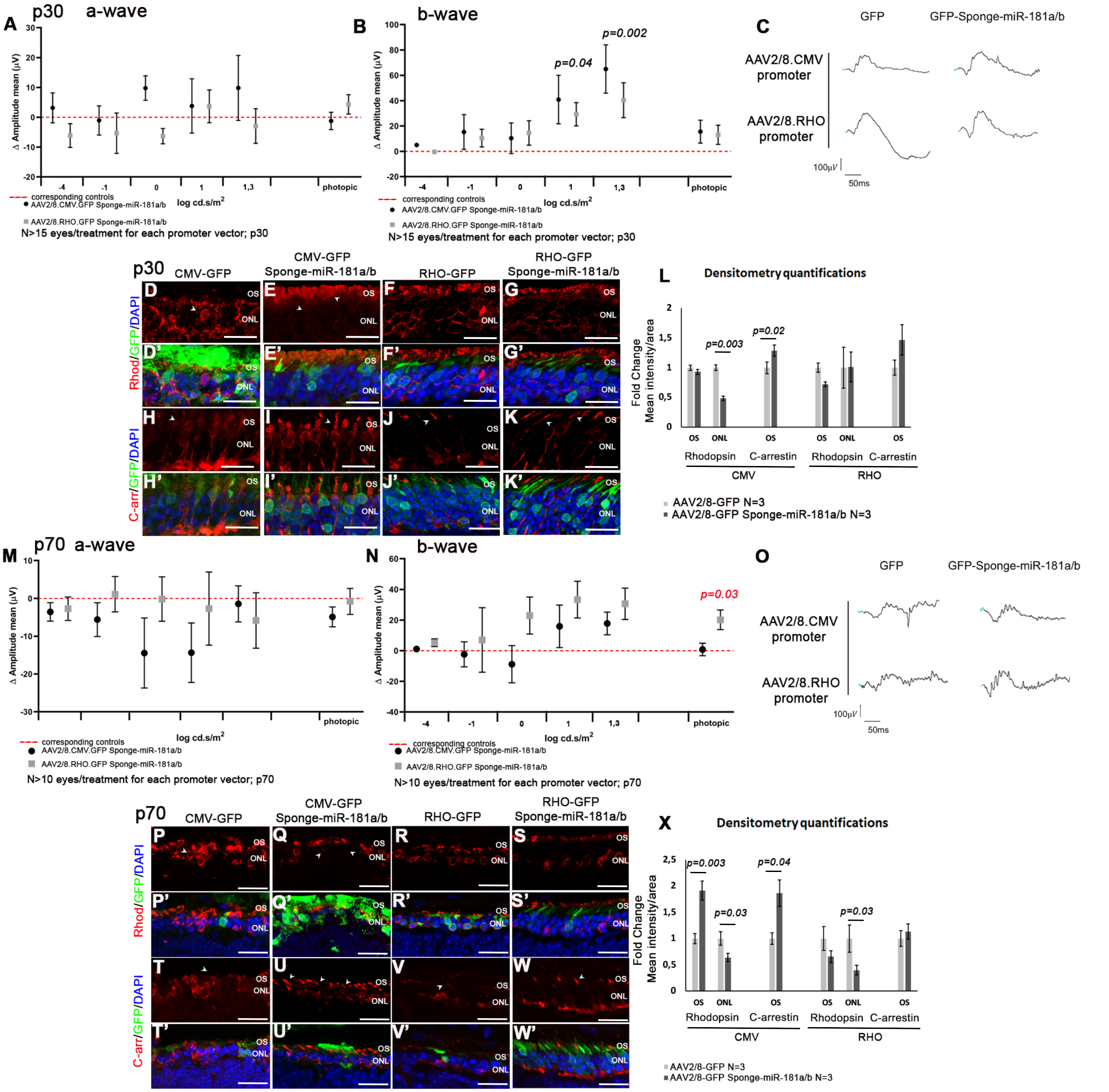

AAV2/8-Sponge-miR-181a/b delivery at p12/14 ameliorates the retinal phenotype of P347S mice. A-C) ERG response (a-wave in A and b-wave in B), reported as Delta (Δ) amplitude in P347S animals injected at p12/14 with AAV2/8.CMV.GFP-Sponge-miR-181a/b and AAV2/8.CMV.RHO-Sponge-miR-181a/b with respect to the corresponding control vectors (AAV2/8.CMV.GFP and AAV2/8.RHO.GFP; red dotted line)) at p30 (N≥15 eyes/treatment, for each promoter vector). P30 representative curves at 20 candles are reported in **(C)**. **D-L)** Immunofluorescence analysis showed amelioration of Rhodopsin localization (p30 D-G') and C-arrestin expression with amelioration of the OS structure (p30 H-K', white arrowheads) in Sponge-miR-181a/b-injected eyes. Scale bars are 25 μm. Fluorescence densitometry quantification of each staining at p30 is reported in L, N=3 eye/treatment for each staining. **M-O)** ERG response (a-wave in M and b-wave in N), reported as Delta (Δ) amplitude in P347S animals injected at p12/14 with AAV2/8.CMV.GFP-Sponge-miR-181a/b and AAV2/8.CMV.RHO-Sponge-miR-181a/b with respect to the corresponding control vectors (AAV2/8.CMV.GFP and AAV2/8.RHO.GFP; red dotted line) at p70 (N≥10 eyes/treatment, for each promoter vector). P70 representative curves at 20 candles are reported in **(O)**. **P-X)** Immunofluorescence analysis showed amelioration of Rhodopsin localization (p70 P-S') and C-arrestin expression with amelioration of the OS structure (p70 T-W', white arrowheads) in Sponge-miR-181a/b-injected eyes. Scale bars are 25 μm. Fluorescence densitometry quantification of each staining at p70 is reported in X, N=3 eye/treatment for each staining. AAV2/8.CMV.RHO-Sponge-miR-181a/b vs corresponding control (AAV2/8.RHO.GFP) p values are reported in red, AAV2/8.CMV.GFP-Sponge-miR-181a/b vs corresponding control (AAV2/8.CMV.GFP) p values are reported in black. Data are presented as mean of Delta (Δ) amplitude ± SD in A, B and M, N. Two-way ANOVA test. Data are presented as mean of Fold Change ± SEM in L and X. Student's t-test, paired.

Appendix Table S1: Primer sequences for qRT-PCR analysis.

Transcript	Forward	Reverse
mmu-*Hprt*	AGCTTGCTGGTGAAAAGGAC	GTCAAGGGCATATCCACAAC
mmu-*Gapdh*	GGTGCTGAGTATGTCGTGGA	CTAAGCAGTTGGTGGTGCAG
mmu-*Bcl2*	ACAACATCGCCCTGTGGATG	GTTTGTCGACCTCACTTGTG
mmu-*Mcl1*	GCTTCATCGAACCATTAGCAG	CCAGCAGCACATTTCTGATG
mmu-*Xiap*	GGTCCTGATTGCAGATCTTG	GTCAACTGCTTCTGCACAC
mmu-*Atg5*	GCCTATATGTACTGCTTCATC	CAACGTCAAATAGCTGACTC
mmu-*Erk2*	CACCAACCTCTCGTACATCG	GTGCCCGGATGATGTCATTG
mmu-*Park2*	CCATCAAGAAGACCACCAAG	CAAGTGACATCTCTCTCTAC
mmu-*Nrf1*	CTTACTGGAGTCCAAGATGC	GGAGCCAACAGAATCCTTTC
mmu-*Cox11*	GAATCCTACTGACAAACCAG	GAGGTCGACATTCACCATTC
mmu-*Coq10*	GATGATCATGGCAGCTCGGA	CTCGCACAGATCTCTTTAGG
mmu-*Prdx3*	GGAGTATTTCTGCCTCAACAG	CTCTCCATTGACAACAGCAG
mmu-*Ppargc1a*	GGAATGCACCGTAAAATCTGC	TTCTCAAGAGCAGCGAAAGC
mmu-*Jak2*	CTACCAGATGGAAACTGTGC	GCCTCTGTAATGTTGGTGAG
mmu-*Stat3*	CAACCAGTCTGTGACCAGAC	GCCAGCTCTTCATCAGTCAG
mmu-*Drp1*	CCCGCATCCATGAGATCAAG	CGTCACTGCTGCAAATACAG
mmu-*Irf1*	CTTCGTCGAGGTAGGACGTG	CTTTGCTGCAGGAGCGATTC
mmu-*BclXl*	CACCTATCTGAATGACCACC	CCACACCAGCCACAGTCATG
mmu-*Pim*	CATGGTCTGCGGAGATATTC	CCGGATTTCTTCAAAGGAGG
mmu-*Birc3(cIAP)*	GCTGAGGTGCTGGGAATCTG	CATCTTCTGGGGAGTCTGAC
mmu-*Birc5(Survivin)*	GAACGAGCCTGATTTGGCCC	CTTGACAGTGAGGAAGGCGC
mmu-*Fos*	GAGAAACGGAGAATCCGAAG	GCAATCTCAGTCTGCAACGC
mmu-*Fgf2*	CTATCAAGGGAGTGTGTGCC	CTGCCCAGTTCGTTTCAGTG
mmu-*Gap43*	GGAGCCTAAACAAGCCGATG	CTGTCGGGCACTTTCCTTAG
