# Supplementary material for: miR‐181a/b downregulation: a mutation‐independent therapeutic approach for inherited retinal diseases
Source: EMBO Mol Med. 2022 Oct 4;14(11):e15941. doi: 10.15252/emmm.202215941 (PMC9641422; doi:10.15252/emmm.202215941)
Supplement: Supplementary file 2 — Expanded View Figures PDF [file EMMM-14-e15941-s002.pdf]

Expanded View Figures

Figure EV1. Effects of miR-181a/b-1 downregulation on PRs in P347S mice at p30.

A–D DAPI staining of P347S (A) and P347S/miR-181a/b$^{+/-}$ (B) retinas highlighted no difference in ONL thickness and number of nuclei in the ONL, as quantified in (C) and (D). N = 3 eyes/genotype. Data are presented as mean ± SEM. Student's t-test, unpaired. Scale bars are 50 μm in (A, B).

E–H Immunofluorescence analysis showed amelioration of Recoverin staining in the ONL and the Inner Plexiform Layer (IPL) in P347S/miR-181a/b$^{+/-}$ versus P347S eyes at p30. Scale bars 25 μm. Fluorescence densitometry quantification of Recoverin staining is reported in (H), N = 3 eye/genotype. WT versus P347S P-values are reported in red, P347S versus P347S/miR-181a/b$^{+/-}$ P-values are reported in black. Data are presented as mean ± SEM. Student's t-test.

I–K Electron microscopy analysis shows increased length of PR OS in P347S/miR-181a/b$^{+/-}$ versus P347S, quantified in (K) (N = 2 animals/genotype); Data are presented as mean ± SEM. Student's t-test, unpaired; Scale bars are 1 μm in (I, J).

© 2022 The Authors

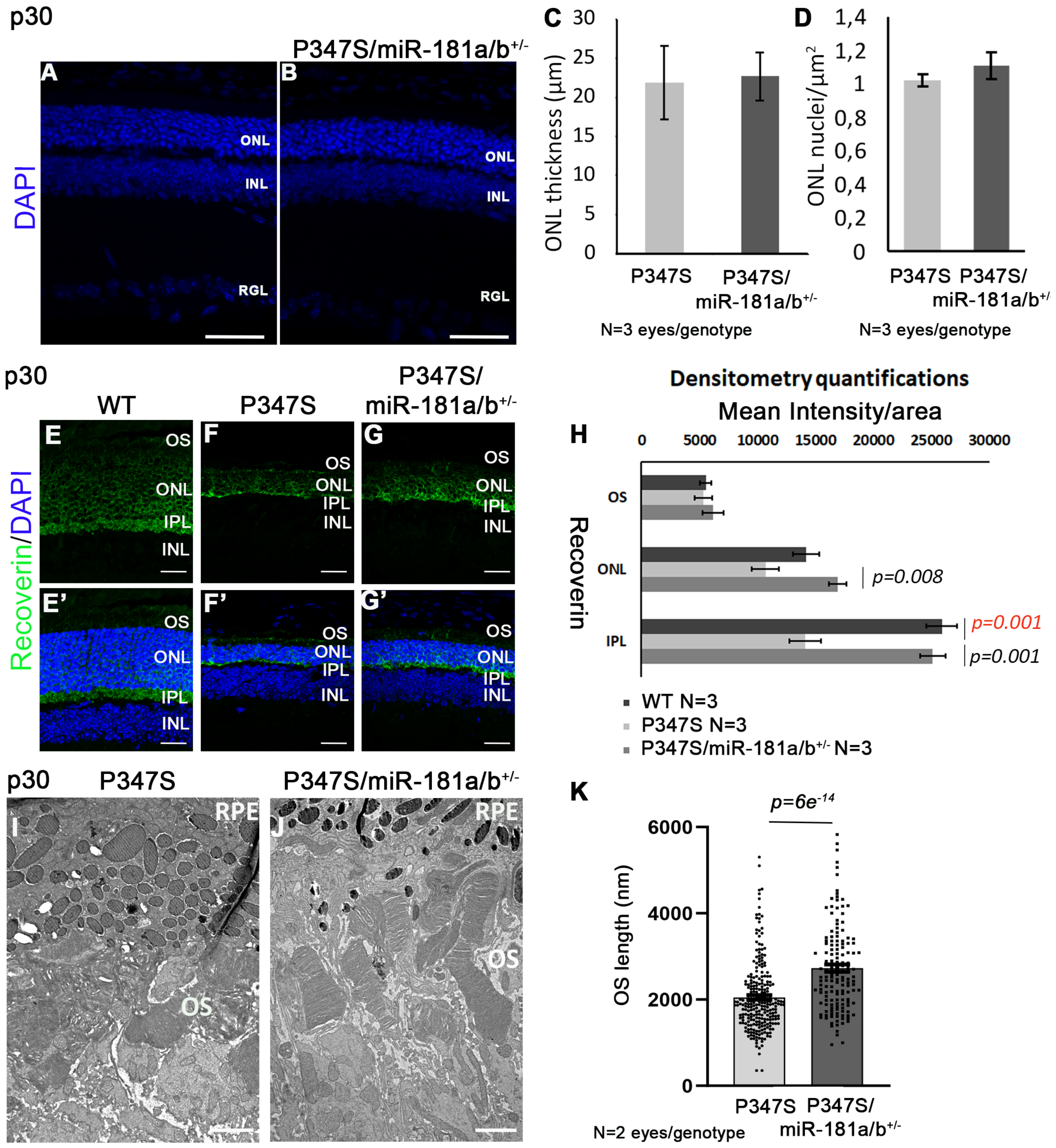

p30
P347S/miR-181a/b+/-
A
B
DAPI
ONL
INL
RGL
C
ONL thickness (µm)
P347S
P347S/ miR-181a/b+/-
N=3 eyes/genotype
D
ONL nuclei/µm2
P347S
P347S/ miR-181a/b+/
N=3 eyes/genotype
p30
WT
P347S
P347S/ miR-181a/b+/-
E F G
E' F' G'
Recoverin/DAPI
OS
ONL
IPL
INL
Densitometry quantifications
Mean Intensity/area
H
Recoverin
p=0.008
p=0.001
p=0.001
WT N=3
P347S N=3
P347S/miR-181a/b+/- N=3
p30
P347S
P347S/miR-181a/b+/-
I
J
RPE
OS
K
p=6e-14
OS length (nm)
P347S
P347S/ miR-181a/b+/-
N=2 eyes/genotype

Figure EV1.

Figure EV2. miR-181a/b-1 downregulation leads to an increase of mitochondrial proteins.

A–E Immunofluorescence analysis showed a decrease of Citrate Synthase (CS) staining, a mitochondrial marker, in the OS of P347S retinas versus WT (A) at p12 (B) and p30 (D). The analysis highlights an amelioration of CS staining in P347S/miR-181a/b$^{+/-}$ versus P347S eyes at both p12 (C) and p30 (E) (Red triangles). (A′–E′) show higher magnification of (A–E). Scale bars 25 μm.

F, G WB analysis reveals decreased levels of OXPHOS, representative of Mitochondrial Respiratory Chain complexes, and CS proteins in the eyes of P347S with respect to WT, and partial rescue of these parameters in P347S/miR-181a/b$^{+/-}$ versus P347S PR at p30 [quantified in (G)]. Data are normalized to p115. N = 3 eyes/genotype.

H, I WB analysis of key proteins involved in mitochondrial fission/fusion pathway (Fis1 Opa1 and Mfn1/2 proteins) in the eyes of WT, P347S and in P347S/miR-181a/b$^{+/-}$ at p30 [quantified in (I)]. Data are normalized to Gapdh. $N \geq 4$ eye/genotype.

Data information: Data are presented as mean of Fold Change ± SEM. Student's *t*-test, unpaired. Please note that all compared bands from WT, P347S and P347S/miR-181a/b$^{+/-}$ samples are from the same blots, which were cropped and shown organized in the panel for the sake of data presentation clarity (see source data).
Source data are available online for this figure.

 © 2022 The Authors

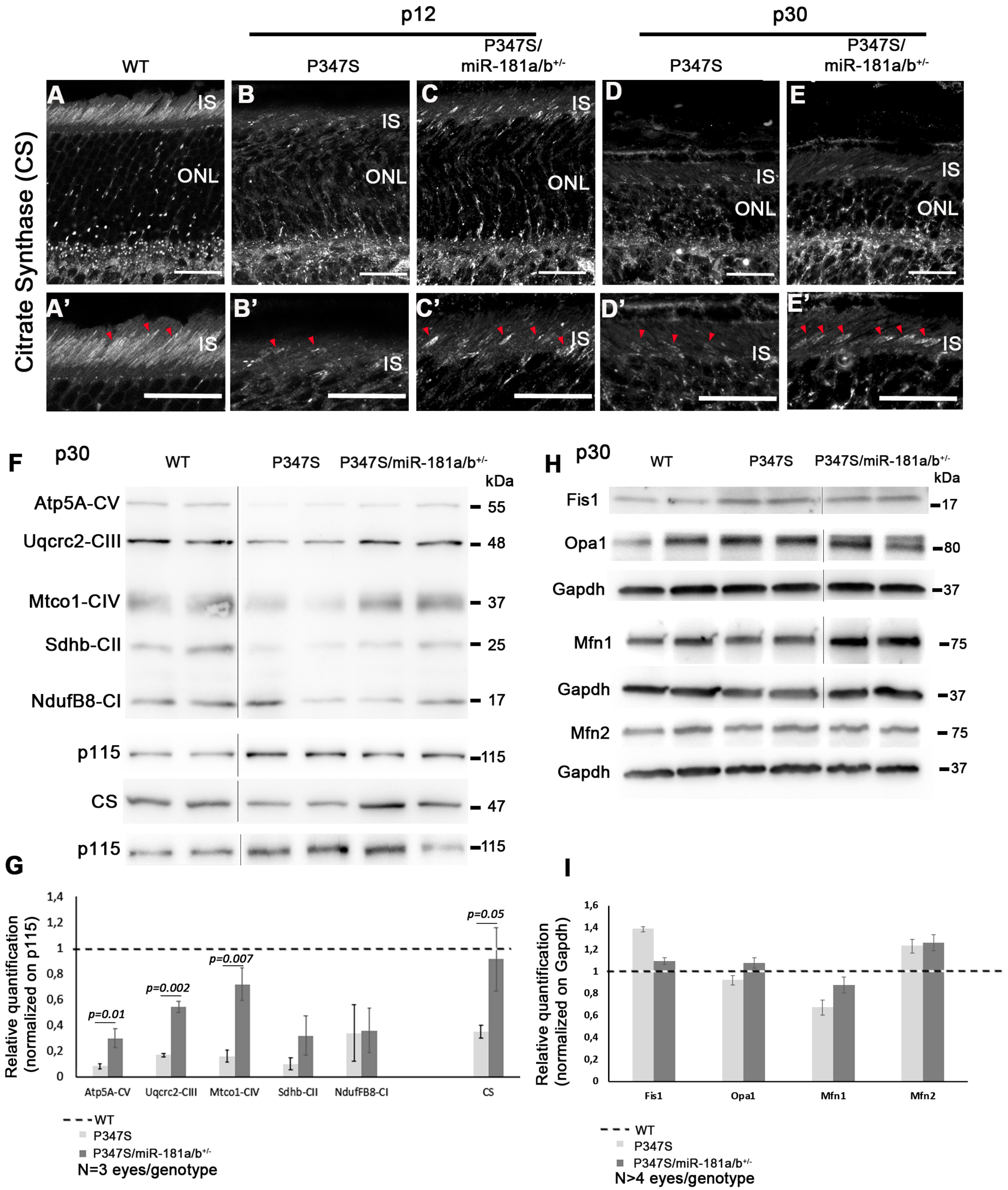
p12
p30
WT
P347S
P347S/ miR-181a/b+/-
Citrate Synthase (CS)
IS
ONL
F p30
Atp5A-CV
Uqcrc2-CIII
Mtco1-CIV
Sdhb-CII
NdufB8-CI
p115
CS
kDa
H p30
Fis1
Opa1
Gapdh
Mfn1
Mfn2
G
Relative quantification (normalized on p115)
p=0.01
p=0.002
p=0.007
p=0.05
Atp5A-CV Uqcrc2-CIII Mtco1-CIV Sdhb-CII NdufFB8-CI CS
WT
P347S
P347S/miR-181a/b+/-
N=3 eyes/genotype
I
Relative quantification (normalized on Gapdh)
Fis1 Opa1 Mfn1 Mfn2
N>4 eyes/genotype

Figure EV2.

Figure EV3. miR-181a/b-1 downregulation is associated with an upregulation of the JAK2/STAT3 pathway.

A, B WB analysis of P347S/miR-181a/b$^{+/-}$ versus P347S total eye protein extracts at p30 showed increased levels of total Stat3 and p705-Stat3. No statistically significant variation was observed for p-727-Stat3. Data are normalized to p115. Relative protein quantifications are reported in (B). $N = 4$ eyes/genotype. Data are presented as mean of Fold Change ± SEM. Student's *t*-test.

C–G Immunofluorescence analysis of Stat3 in P347S and P347S/miR-181a/b$^{+/-}$ retinas at p30. (E, F) and (E′, F′) show higher magnification of (C, D) and (C′, D′), respectivey. Scale bars 25 μm. Fluorescence densitometry quantification of Stat3 in the ONL is reported in (G), $N = 3$ eye/genotype for each staining. Data are presented as mean ± SEM. Student's *t*-test, unpaired.

H qRT–PCR analysis reveals increased levels of p705-Stat3 transcriptional target genes in the eyes of P347S/miR-181a/b$^{+/-}$ versus P347S animals. $N \geq 4$ eyes/genotype. Data are presented as mean of Fold Change ± SEM. Student's *t*-test, unpaired.

I, J WB analysis of total Stat3 and p705-Stat3 in the optic cup of P347S DMSO-treated, P347S/miR-181a/b$^{+/-}$ DMSO-treated and of P347S/miR-181a/b$^{+/-}$ FEDRATINIB-treated (an inhibitor of the JAK2/STAT3 pathway) mice at p30 [quantified in (J)] revealed that FEDRATINIB is inhibiting JAK2 activity in the treated samples as showed by the reduction of p705-Stat3 protein levels in P347S/miR-181a/b$^{+/-}$ FEDRATINIB-treated. Data are normalized to Gapdh. $N \geq 4$ eye/genotype. Data are presented as mean of Fold Change ± SEM. Student's *t*-test, unpaired.

Source data are available online for this figure.

© 2022 The Authors

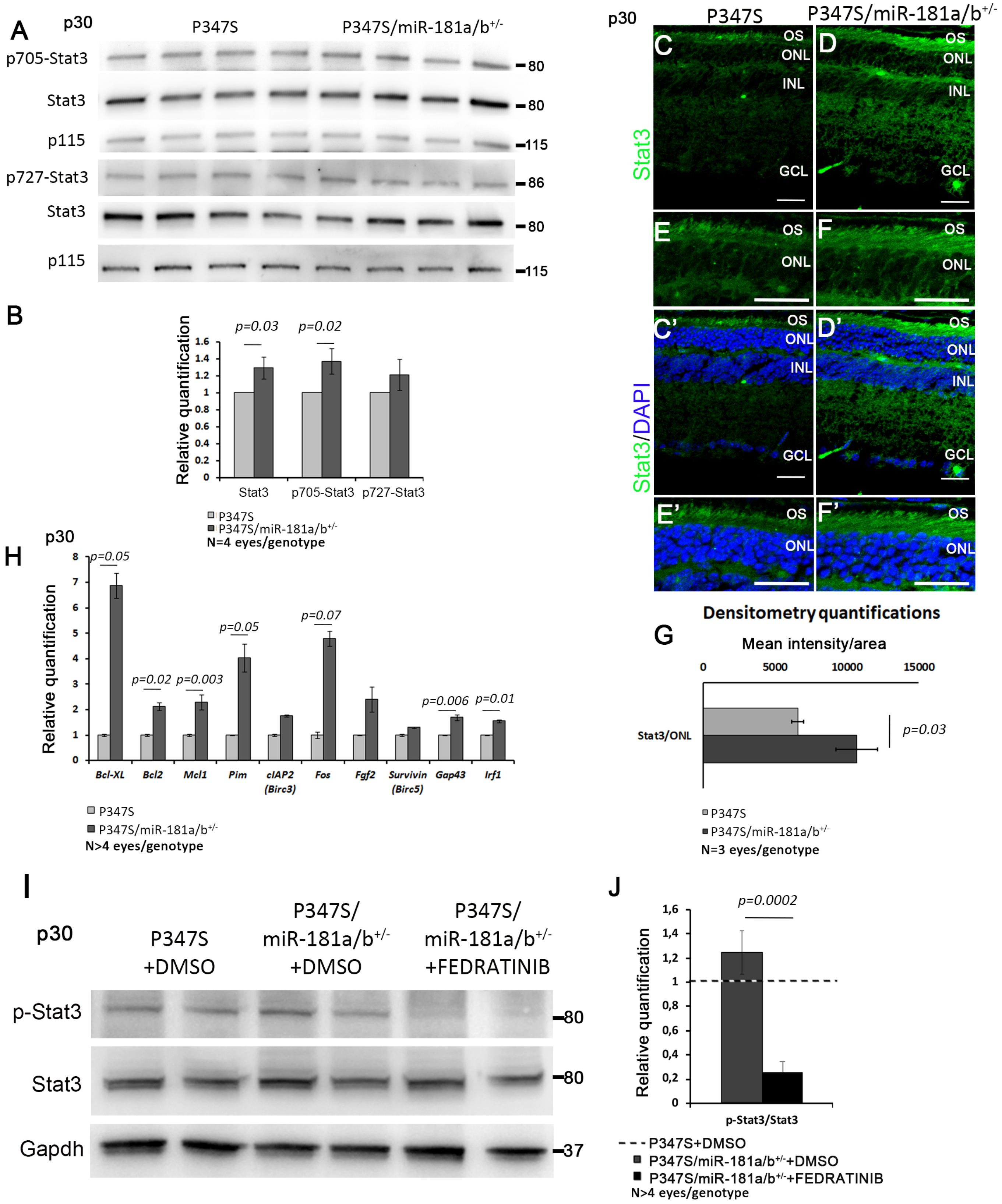

A p30
P347S
P347S/miR-181a/b+/-
p705-Stat3
Stat3
p115
p727-Stat3
Stat3
p115
80
80
115
86
80
115
B
Relative quantification
p=0.03
p=0.02
Stat3
p705-Stat3
p727-Stat3
P347S
P347S/miR-181a/b+/-
N=4 eyes/genotype
p30
P347S
P347S/miR-181a/b+/-
C
D
E
F
C'
D'
E'
F'
OS
ONL
INL
GCL
Stat3
Stat3/DAPI
H
p30
Relative quantification
p=0.05
p=0.02
p=0.003
p=0.05
p=0.07
p=0.006
p=0.01
Bcl-XL
Bcl2
Mcl1
Pim
cIAP2 (Birc3)
Fos
Fgf2
Survivin (Birc5)
Gap43
Irf1
P347S
P347S/miR-181a/b+/-
N>4 eyes/genotype
G
Densitometry quantifications
Mean intensity/area
Stat3/ONL
p=0.03
P347S
P347S/miR-181a/b+/-
N=3 eyes/genotype
I
p30
P347S +DMSO
P347S/ miR-181a/b+/- +DMSO
P347S/ miR-181a/b+/- +FEDRATINIB
p-Stat3
Stat3
Gapdh
80
80
37
J
Relative quantification
p=0.0002
p-Stat3/Stat3
P347S+DMSO
P347S/miR-181a/b+/-+DMSO
P347S/miR-181a/b+/-+FEDRATINIB
N>4 eyes/genotype

Figure EV3.

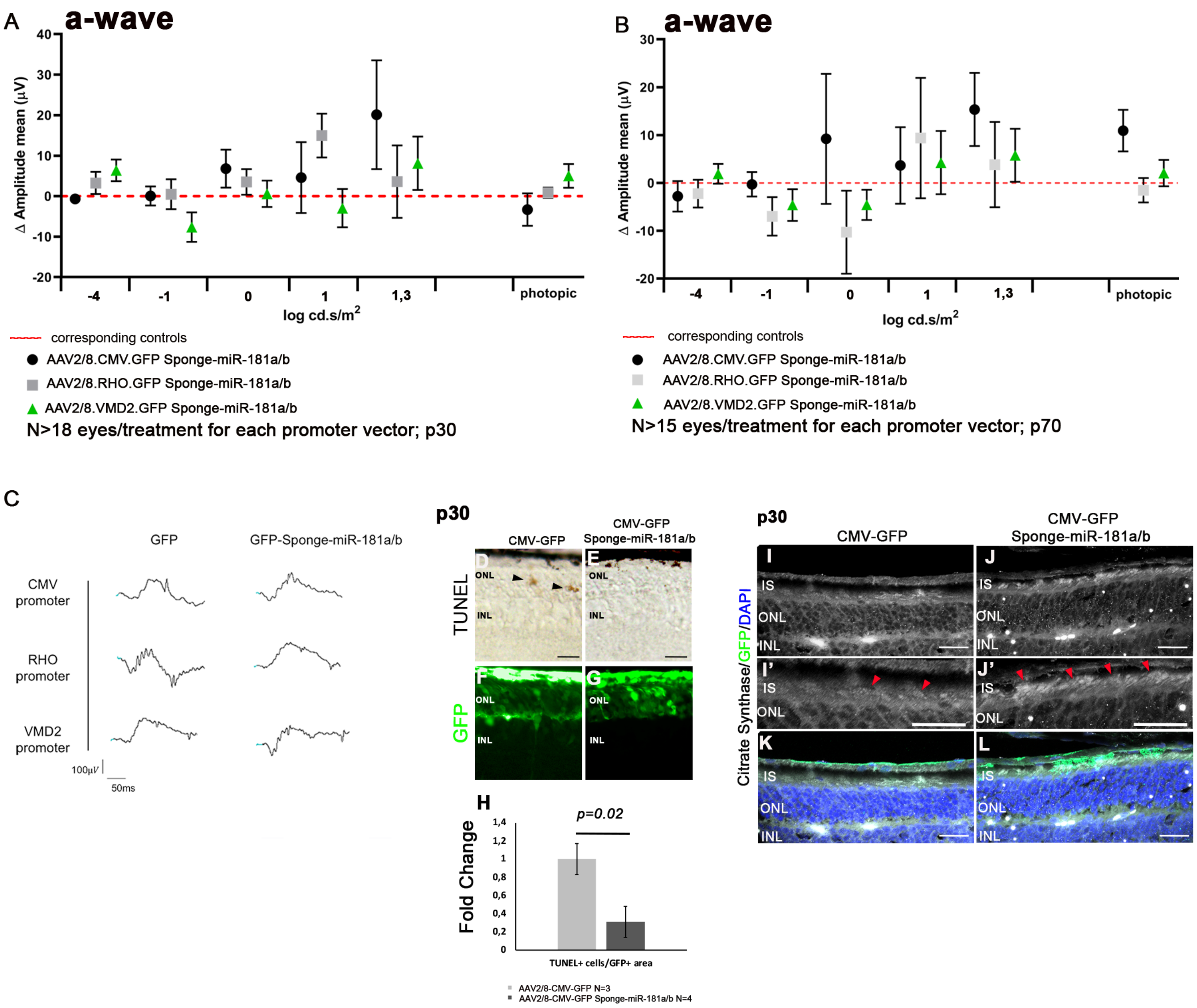

Figure EV4. AAV2/8-Sponge-miR-181a/b delivery improves retinal function and slows down degeneration in P347S mice.

A–C ERG response (a-wave) reported as Delta (Δ) amplitude in P347S animals injected at p4 with AAV2/8.CMV.GFP-Sponge-miR-181a/b, AAV2/8.CMV.RHO-Sponge-miR-181a/b, AAV2/8.VMD2.GFP-Sponge-miR-181a/b vectors with respect to the corresponding controls (AAV2/8.CMV.GFP, AAV2/8.RHO.GFP and AAV2/8.VMD2.GFP; red dotted line) at p30 (A; $N \geq 18$ eyes/treatment, for each promoter vector) and p70 (B; $N \geq 15$ eyes/treatment, for each promoter vector). Data are presented as mean of Delta (Δ) amplitude ± SD. Two-way ANOVA test. P70 representative curves at 20 candles are reported in (C).

D–H TUNEL analysis in the ONL of AAV2/8.CMV.GFP (D, F; black triangles $N = 3$) and AAV2/8.CMV.GFP-Sponge-miR-181a/b (E, G; $N = 4$) sub-retinally-injected P347S retinas at p30; quantification in (H). Data are presented as mean of Fold Change ± SEM. Student's *t*-test.

I–L Immunofluorescence analysis of Citrate Synthase staining (Red triangles) in AAV2/8.CMV.GFP-Sponge-miR-181a/b (J, J′, L) versus AAV2/8.CMV.GFP (I, I′, K). (I′–J′) show higher magnification of (I–J). Scale bars are 25 μm.

 © 2022 The Authors

Figure EV5. AAV2/8-Sponge-miR-181a/b delivery ameliorates the retinal phenotype of *rd10* mice.

A–E Immunofluorescence analysis of Recoverin at p30 in p4-injected- (A–B′) and in p10-injected-*rd10* (C–D′) with AAV2/8.CMV.GFP-Sponge-miR-181a/b (B, B′ and D, D′) and the corresponding control (AAV2/8.CMV.GFP; A, A′ and C, C′). Scale bars are 25 μm. Fluorescence densitometry quantification of Recoverin staining is reported in (E), N = 3 eye/treatment for each staining. Data are presented as mean of Fold Change ± SEM. Student's t-test.

F–I″ Immunofluorescence analysis of Citrate Synthase (Red triangles) at p30 in p4-injected- (F–G″) and in p10-injected-*rd10* (H–I″) with AAV2/8.CMV.GFP-Sponge-miR-181a/b (G–G″ and I–I″) the corresponding control (AAV2/8.CMV.GFP F–F″ and H–H″). (F′–H′) show higher magnification of (F–I). Scale bars are 25 μm.

J–L ERG response in *rd10* animals injected at p4 [a-wave in (J) and b-wave in (K)] with AAV2/8.CMV.GFP-Sponge-miR-181a/b with respect to the corresponding control vectors (AAV2/8.CMV.GFP) at p30 (N = 8 eyes/treatment). Data are presented as mean ± SD. Two-way ANOVA test. Representative curves at 20 candles are reported in (L).

M–O ERG response in *rd10* animals injected at p10 [a-wave in (M) and b-wave in (N)] with AAV2/8.CMV.GFP-Sponge-miR-181a/b with respect to the corresponding control vectors (AAV2/8.CMV.GFP) at p30 (N = 10 eyes/treatment). Data are presented as mean ± SD. Two-way ANOVA test. Representative curves at 20 candles are reported in (O).

© 2022 The Authors

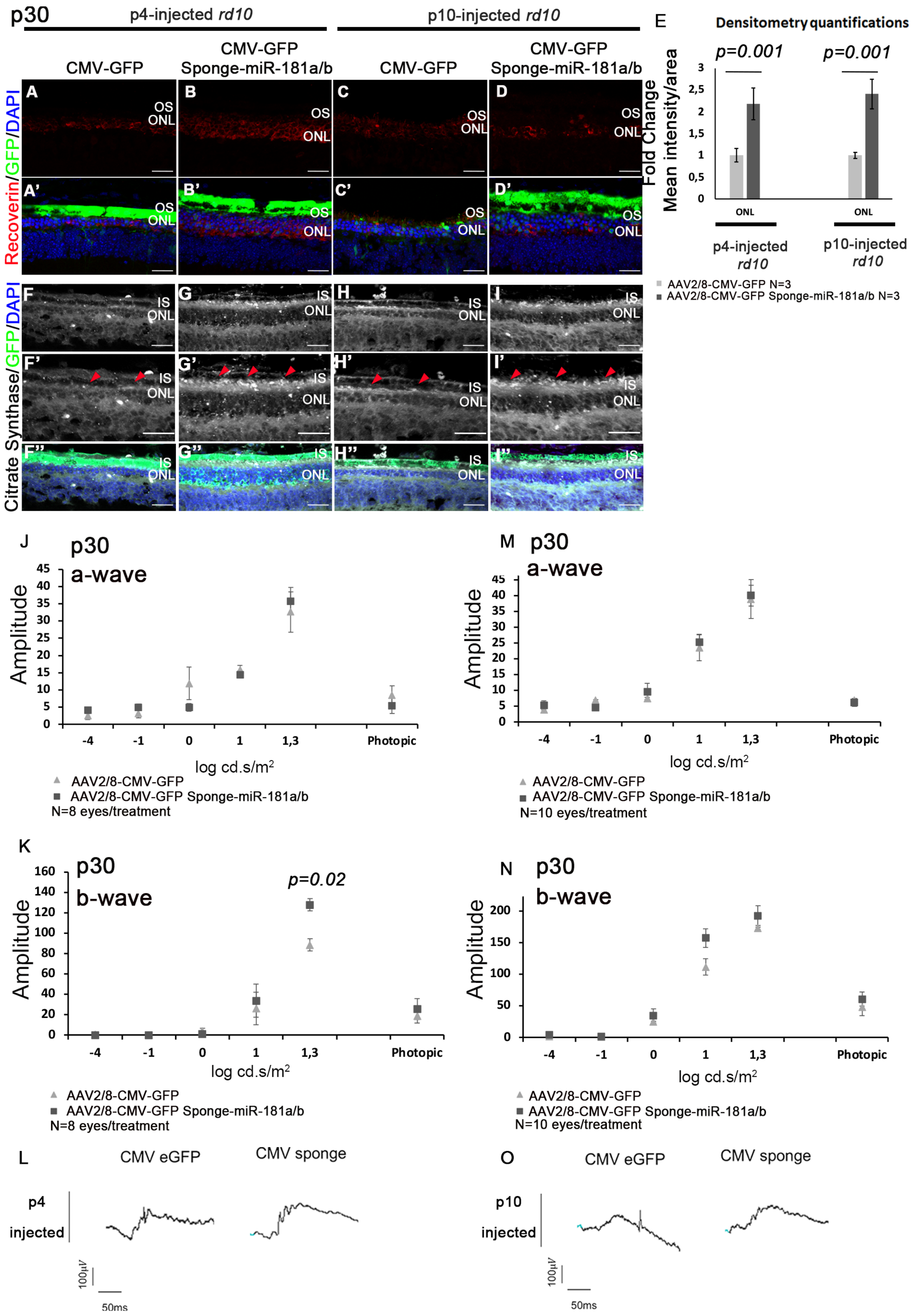

Figure EV5.

© 2022 The Authors
