# Supplementary figures and images for: miR‐181a/b downregulation: a mutation‐independent therapeutic approach for inherited retinal diseases
Source: EMBO Mol Med. 2022 Oct 4;14(11):e15941. doi: 10.15252/emmm.202215941 (PMC9641422; doi:10.15252/emmm.202215941)

Source Data Figure 5A

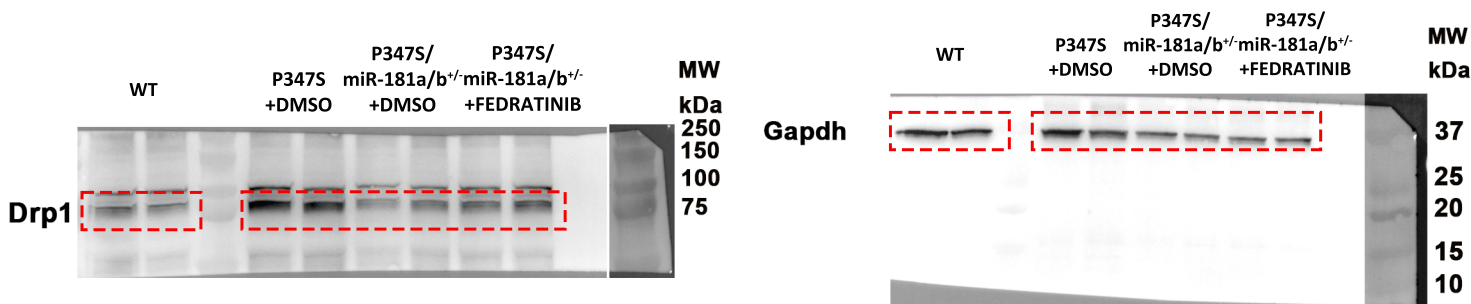

Supplement: Supplementary file 4 — Source Data for Figure 5 [file EMMM-14-e15941-s003.pdf]

Source Data Figure EV3A

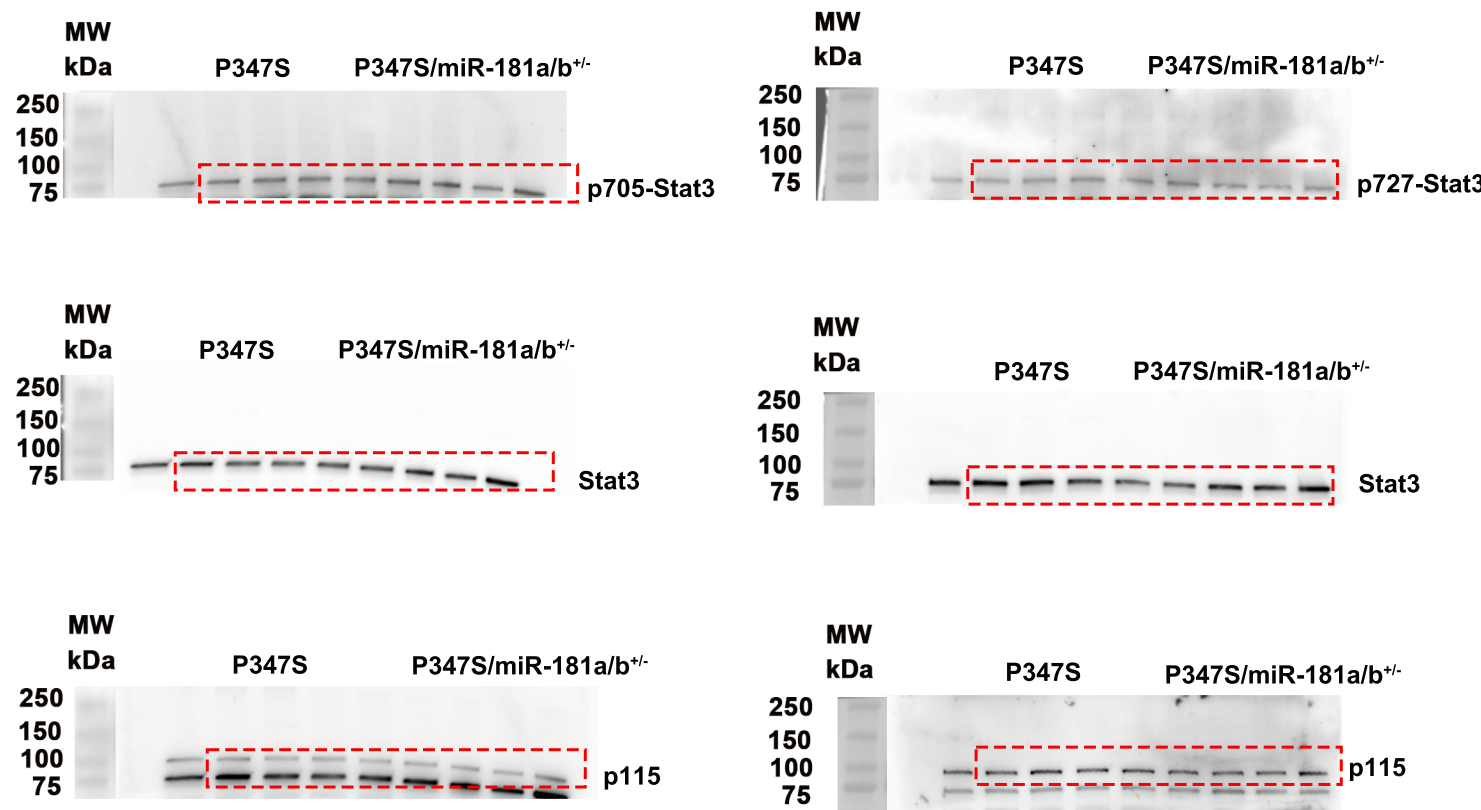

Source Data Figure EV3I

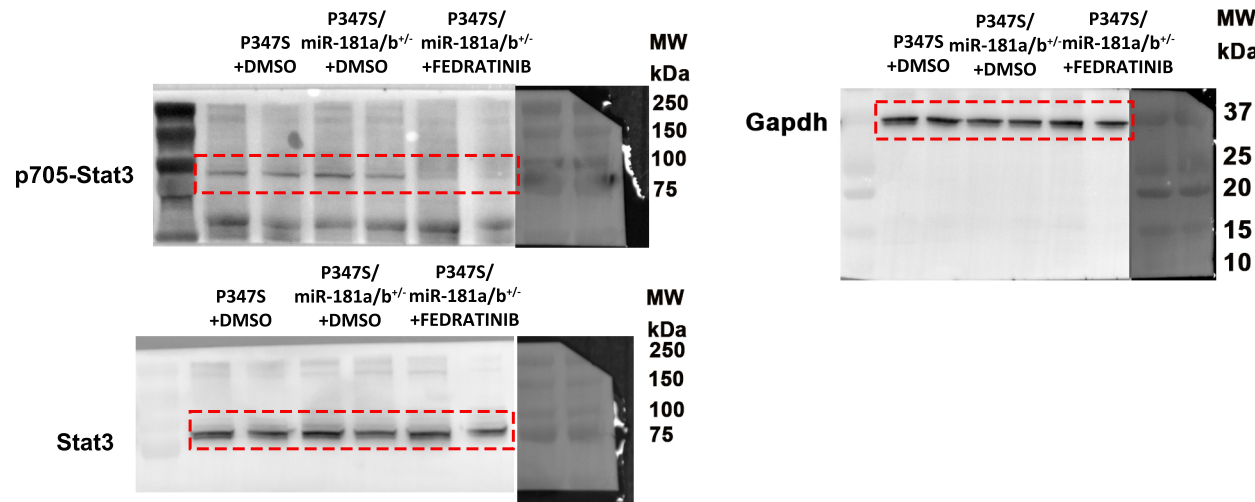

Supplement: Supplementary file 5 — Source Data for Expanded View [file EMMM-14-e15941-s005.zip › EMM-2022-15941_Source data fig EV3.pdf]

Source Data Figure EV2F

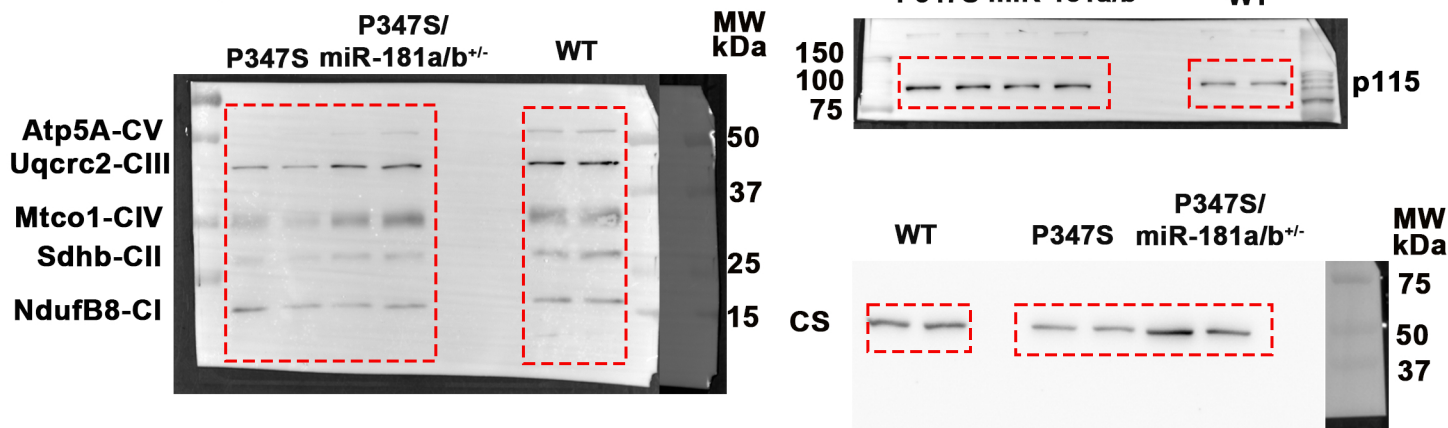

Source Data Figure EV2H

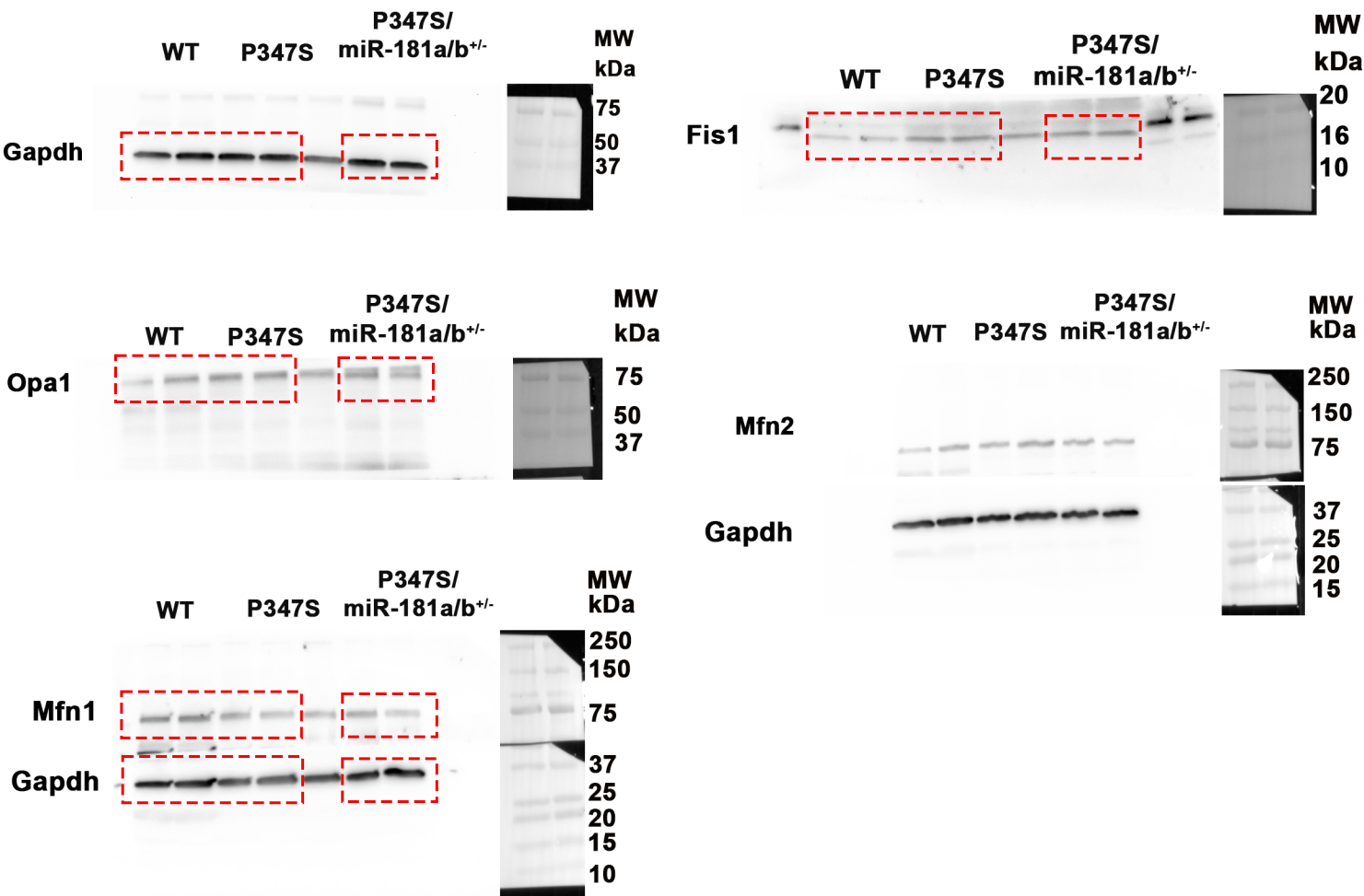

Supplement: Supplementary file 5 — Source Data for Expanded View [file EMMM-14-e15941-s005.zip › EMM-2022-15941_Source data fig EV2.pdf]
