# Supplementary material for: miR‐181a/b downregulation: a mutation‐independent therapeutic approach for inherited retinal diseases
Source: EMBO Mol Med. 2022 Oct 4;14(11):e15941. doi: 10.15252/emmm.202215941 (PMC9641422; doi:10.15252/emmm.202215941)
Supplement: Supplementary file 7 [file EMMM-14-e15941-s004.pdf]

EMBO Molecular Medicine

miR-181a/b downregulation:a mutation-independent therapeutic approach for Inherited Retinal Diseases

Sabrina Carrella, Martina Di Guida, Simona Brillante, Davide Piccolo, Ludovica Ciampi, Irene Guadagnino, Jorge Garcia Piqueras, Mariateresa Pizzo, Elena Marrocco, Marta Molinari, Georgios Petrogiannakis, Sara Barbato, Yulia Ezhova, Alberto Auricchio, Brunella Franco, Elvira De Leonibus, Enrico Maria Surace, Alessia Indrieri, and Sandro Banfi
DOI: 10.15252/emmm.202215941

Corresponding authors: Sabrina Carrella (carrella@tigem.it) , Sandro Banfi (banfi@tigem.it)

Review Timeline:

Submission Date:	23rd Feb 22
Editorial Decision:	14th Mar 22
Appeal Received:	23rd Mar 22
Editorial Decision:	25th Mar 22
Revision Received:	29th Jul 22
Editorial Decision:	8th Sep 22
Revision Received:	15th Sep 22
Accepted:	19th Sep 22

Editor: Lise Roth

Transaction Report:

(Note: With the exception of the correction of typographical or spelling errors that could be a source of ambiguity, letters and reports are not edited. Depending on transfer agreements, referee reports obtained elsewhere may or may not be included in this compilation. Referee reports are anonymous unless the Referee chooses to sign their reports.)

14th Mar 2022

Decision on your manuscript EMM-2022-15941

Dear Dr. Carrella,

Thank you for the submission of your manuscript to EMBO Molecular Medicine. We have now received feedback from the three referees who agreed to evaluate your work.
As you will see from the reports below, while referee #2 supports publication of the work (pending revisions), referee #1 in particular, but also the other referees, raise major concerns on your study. Given the nature of these concerns and considering that at EMBO Press we encourage one round of revisions only in a reasonable time frame, I am afraid I see little choice but to return the manuscript to you at this point with the decision that we cannot offer to publish it.

I am very sorry to disappoint you in this occasion, and hope that the referees' comments are helpful in your continued work in this area.

With kind regards,

Lise Roth

Lise Roth
Editor
EMBO Molecular Medicine

***** Reviewer's comments *****

Referee #1 (Comments on Novelty/Model System for Author):

(1) Mouse strains
1.1. The authors did not disclose the source and the specific line of P347S transgenic mice used in this paper. The original P347S transgenic mouse paper (PMID: 8943080) describes two lines, which exhibited distinct degenerate rates. Which specific line was used in this paper?

1.2. Importantly, P347S mice were on the background C57BL/6 (Charles River) (Line 387), and miR-181 deficient mice (on the background of C57BL/6 (National Cancer Institute). However, except for C57BL/6J (from Jackson Lab), C57BL/6 mice from almost all other vendors tested (including Charles River) are contaminated with the rd8 (retinal degeneration 8) allele (PMID: 22447858). The littermates with or without rd8 could have degenerated at a different rate. The authors should reveal the rd8 genotyping results to rule out the differential phenotypes that are contributed by the presence of the rd8 allele.

Referee #1 (Remarks for Author):

Previously, these authors suggested the inactivation of miR-181a/b can ameliorate the disease phenotype in Leber's hereditary optic neuropathy mouse models. In the current communication, this author sought to test the hypothesis that miR-181a/b deficiency can protect photoreceptors in retinal degeneration mouse models. It remains challenging to treat inherited retinal degeneration (IRD) diseases due to a wide range of genetic mutations. Both the transgenic and AAV injection approaches were undertaken. P347S rhodopsin mutant mouse line was tested by both methods. Rd10 mice were tested by the latter method. Several "endpoints" were used singly or together, including immunostaining photoreceptor proteins and dark ERG b-wave. Additional corrective evidence linking the compromised mitochondria functional pathway in the disease and rescue was provided. These results prompted the authors to conclude that miR-181a/b deficiency can provide a beneficial effect in treating multiple types of retinal degeneration diseases. As detailed below, the current presentation lacks rigor in the background, experimental designs, data quality, and data interpretation. Despite the importance of the subject investigated, several technical concerns fall short in concluding the miR-181a/b deficiency has a cell-protective role of photoreceptor cells in retinal degeneration mouse models.

Major concerns.

(1) Mouse strains
1.1. The authors did not disclose the source and the specific line of P347S transgenic mice used in this paper. The original P347S transgenic mouse paper (PMID: 8943080) describes two lines, which exhibited distinct degenerate rates. Which specific line was used in this paper?

1.2. Importantly, P347S mice were on the background C57BL/6 (Charles River) (Line 387), and miR-181 deficient mice (on the background of C57BL/6 (National Cancer Institute). However, except for C57BL/6J (from Jackson Lab), C57BL/6 mice from almost all other vendors tested (including Charles River) are contaminated with the rd8 (retinal degeneration 8) allele (PMID: 22447858). The littermates with or without rd8 could have degenerated at a different rate. The authors should reveal the rd8 genotyping results to rule out the differential phenotypes that are contributed by the presence of the rd8 allele.

(2) Endpoints.
2.1. The original paper of P347S mice emphasized the rhodopsin immunostaining was indistinguishable from the WT controls (Figs. 2 and 3 in PMID: 8943080). So it is puzzling why the authors show the rhodopsin mislocalization in the P347S mice and used it as an endpoint (Fig. 1I-Jm Fig. 2A-D, Fig. 5)?
2.2. The other readouts used, such as the thickness of the photoreceptor nuclear layer (ONL), the length of the inner segment (by F-actin and the lengths of the outer segment (by TEM) highly subject to the sectioning planes and the survey areas. Both of this and the single data point cannot convincingly demonstrate the restoration of the photoreceptor structures.
2.3. The mitochondrial populations in photoreceptors are highly heterogeneous, their shape and sizes are dependent on the distance to the outer segment, the time of the harvest, etc. Thus, it is not reliable to depict the size based on the single TEM images, as used by this communication without rigorous experimental description (Fig. 3B-F). Recent investigators have resorted to 3D scanning EM instead due to the problems (e.g., PMID: 34552195; PMID: 33144507).
The Scale Bars of all figures, including EM, are missing.
2.4. The time point for the mitochondria characterization was when the photoreceptors already look dead (Fig. 3C). Why not be assayed at PN12 to match the transcriptome analysis and to directly address the mitochondria atrophy is an "early" event, i.e., a cause vs. consequence of the cell death.
2.5. Scotopic ERG a-wave represents photoreceptor functions. Several P347S mouse lines still have detectable a-wave (PMID: 8943080). It is curious why the a-wave results were not shown, but only the b-wave data were shown. Since b-wave represents the inner retinal neuron function, the positive results cannot directly conclude the improvement of photoreceptor function. Please show the Scotopic ERG a-wave (taken at different light intensity illumination) of both mutant and WT control mice. The latter control will help to register the extent of the functional restoration relative to the WT mice.
2.6. The authors showed the PNA staining is better preserved in the double transgenic mice (Fig. 1). Since PNA was typically used to highlight the cone outer segments, does the miR-181a/b deficiency improve the cone photopic ERG (a and b waves)?
2.7. To claim for the cell-protective role of miR181a/b, one might have to show the slow-down of the photoreceptor cell death or prevent cell death for early intervention. These are not the case in the current presentation. Fig. 2E shows the ONL thickness was increased from 5 to 6 uM. The minor, albeit "statistically significant", the increase was unlikely to be physiologically relevant, considering the WT ONL is up to 50-um thick. The ONL thickness is region-dependent (~50um for central, and ~25um for periphery). Unfortunately, the regional variation was not considered in any of the ONL thickness quantification (Fig. 2E, Fig. EV 1C). Hence, calling miR-181a/b deficiency has a protective role (Lines 253, 262, 772, ...etc.) is an overstatement.
2.8. Although the cell-type specificity was well demonstrated for the AAV studies, only a small region was shown. Please show the low-magnification view of the entire retina globe for the overall infection rate. Were the eyes with injection-related trauma been excluded for consideration? This information is useful for the consideration of the ERG results, which should be carried out using the criteria as described above.

2.9. The authors used either "eyes" or "tissues" in describing the procedures for RNA isolation (qPCR, transcriptome) and protein isolation (Lines 396, 399, 417, 790). What tissues? Were the whole eyes (containing many cell types including the lens, cornea, etc.) were used? The changes in the expression of the miR181a/b targets or mitochondria functions of the mixed cell population do not necessarily suggest that these genes are changed in the photoreceptors. Can the authors demonstrate any of the proteins were changed in the photoreceptor cell layers by immunostaining?

3. Others

3.1. The sources of the mouse lines should be acknowledged.

3.2. Methods should be written at a detailed level that others can reproduce.

3.3. The vectors, the helpers, the cell systems, and the purification of AAV should be detailed.

3.4. Erroneous statement or overstatement.

Since only correlative data was provided, LINE 109. "We also observed amelioration of mitochondria morphology in PRs, which is mediated by regulation of the mitochondria fission protein Dra1, mainly via the activation of the Jak2/Stat3 pathway" is an overstatement.

Line 60. IRD represents the most important cause of vision impairment in the Western population." is an erroneous statement.

LINE 101-103 is an erroneous statement. There is no experimental data that supports that mitochondria dysfunction is an "early event" of photoreceptor cell death in P347S rhodopsin mutation. The transcriptome/qPCR/proteins data were obtained from multiple eye cell types. The mitochondria characterization of photoreceptors was carried out at the time point when the photoreceptors were severely atrophic (see above).

Discussion should be grossly truncated and focus on discussing the experimental results. Speculation should be stated so whenever it is complying.

Referee #2 (Comments on Novelty/Model System for Author):

All models used are well established models in the study of IRD.

Referee #2 (Remarks for Author):

The paper titled miR-181a/b downregulation: a mutation-independent therapeutic approach for Inherited Retinal Diseases (IRD) by Sabrina Carrella builds on previous publications demonstrating the toxicity of mir-181 in response to retinal stress.
This work further demonstrates the use of miRNA as possible therapeutic approaches for reducing the severity and progression of degeneration.
The paper provides a strong overview of the current gene therapy landscape and does well to introduce the importance of the topic and the relevance of the studying mir-181. In particular the role of miR-181 to regulate mitochondrial biogenesis is important to the study of IRD as mitochondrial dysfunction is known to be significantly involved with disease onset and progression.
This is a really strong set of data, and a very well written manuscript. I have no hesitation recommending this for publication, with some minor tweaks and suggestions indicated below.

Minor improvements:
Results
As a general critique suggest including the number of animals and the age of the animals in each figure to clearly identify the variabilities in each data set.
Figure 1:
1. Only A wave responses are shown in figure 1F. Strongly suggest showing representative images of the ERG traces and also include B wave data as well.
2. Optical densitometry quantification of the microscopy images to quantify the differences in intensity and locations of the various retinal stains will strengthen this important data.

Figure 2:
1. For consistency in the P90 aged animals please repeat all the same immunohistochemistry straining as per figure 1. This would strengthen the manuscript by showing and comparing the differences between P30 and P90 days.
2. Once again please show the quantification of microscopy images and also the ERG B wave data and representative EGRs as well.

Figure 3:
1. Further context to the RNAseq data is required. What analysis was performed and while it is mentioned this is a reanalysis, further detail in the figure should be provided. Perhaps a table of associated genes used for the analysis would be beneficial, or a list of genes associated with the specific pathways identified.
2. This is true for all figures but especially for figure 3 E and F. It is difficult to see which is the P values associated with each comparison. Suggest either use lines or * to indicate the significance as well as which are the comparisons being explored.
3. While not significant I would suggest including the data of the mitochondrial number in this section. It is good data to show that there is no change in number, but a change in their morphology. This data's inclusion would help make this point more clearly.

Figure 4:
1. The WB data is not particularly strong. Especially the GAPDH staining which has not provided a great reference level. Particularly for the P347S values which appear significantly stronger than other parts of the gel. Authors should comment on this or redo this analysis.
2. All WB data should be shown. At present only representative images for Drp1 are provided. Please provide this data for all other WB performed.

Figure 5:
1. Again further ERG data is required and the representative images. Also there is no control ERG shown as a baseline reading.

This should be included.
2. The GFP labelling is not shown anywhere in the main images, so it is unclear if the AAV transfection was successful (Note some are placed in the supplementary). Quantification and qualitative data is required to validate these findings and this should all be included in the main results not in the supplementary section.

Figure 6:
1. Justification of the different functional tests are required. Did the ERGs not show a positive result? Suggest consistency of tests across the experiments reported in this manuscript.

Discussion
Really strong discussion with no suggestions for improvement. This was a pleasure to read.

Referee #3 (Comments on Novelty/Model System for Author):

Focusing on mitochondria is not new to rescue inherited retinal diseases but focusing on miR-181a/b is novel approach and impact is high if true. They have already published one paper in EMBO Mol Med for the basics of this micro RNA to rescue and maintain mitochondria structure.

Referee #3 (Remarks for Author):

Submitted manuscript by Carrella et al., focus on miR-181a/b, which targets number of gene transcript of cox11, erk2, mcl1, bcl2, nrf1, coq10b, park2 and others leading to the biogenesis, clearance and detoxification ROS in mitochondria. Authors demonstrate induction of drp1 protein by miR-181a/b suppression mainly via activation of Jak2/Stat3 pathway by QT-PCR. Author have shown previously that inactivation of miR-181a/b protects RGC in LHON mice model. In this manuscript authors have used the same method on P347S rhodopsin dominant and recessive rd10 retinitis pigmentosa mouse models. AAV2/8 vector was used to deliver RHO-Sponge-miR-181a/b into the subretinal space for P347S Tg-mice and rd10 mice. miR-181a/b-1 +/+ mice was mated with P347S Tg-mice to generate genetically suppressed mice of miR-181a/b. The suppression was performed by Suppression of miR-181a/b do not cause abnormality in normal mice.

Comments to authors,

Authors has shown results of rescuing both dominant and recessive RP by modulating mitochondria through miR-181a/b suppression. The aim is promising but additional information is needed for positive conclusion.

1.Please add scale for all photos of tissue sections in this manuscript.

2.Fig.1: Please show photo of full retina to observe full structure of the photoreceptor and other area of retina.

3.Fig.3B: Please add scale and organelle name in the photo.

4.Fig.5 & Fig.6: Additional immunostaining of endogenous photoreceptor makers is needed to assess the condition of the retina. Normal mouse section should be added for this assessment and the entire retina should be shown. Mitochondria staining should be also added to see how much is remaining compare to normal retina and non-treated disease mice.

As a service to authors, EMBO provides authors with the possibility to transfer a manuscript that one journal cannot offer to publish to another EMBO publication. The full manuscript and if applicable, reviewers reports are automatically sent to the receiving journal to allow for fast handling and a prompt decision on your manuscript. For more details of this service, and to transfer your manuscript to another EMBO title please click on Link Not Available

Please do not share this URL as it will give anyone who clicks it access to your account.

Pozzuoli, 21-03-2022

Dear Dr. Roth,

thanks a lot for handling our manuscript (EMM-2022-15941). As you can understand, we are a little disappointed by your decision. Indeed, we feel that, based on the Reviewers' comments, there is room for sending you a revised version of the paper that can satisfy their major concerns. In particular, we feel that most of Reviewer 1's concerns about insufficient rigor in data interpretation derive mostly from misunderstanding and from easily addressable technical issues.

In the enclosed point-to-point response, we specifically address all the major concerns raised by Reviewers 1 and 3. As you will see, we believe that we can provide a revised version of the manuscript in a reasonable timeframe with a limited number of additional experiments required.

We hope you can give us the opportunity to proceed with the submission of a revised version of the manuscript and we look forward to hearing from you.

Sincerely,

Sandro Banfi, MD
Professor of Medical Genetics
Dept. of Precision Medicine
University of Campania "Luigi Vanvitelli"
via Luigi De Crecchio 7, 80138 Naples, Italy

Associate Investigator
TIGEM (Telethon Institute of Genetics and Medicine)
Via Campi Flegrei 34, 80078 Pozzuoli (Naples), Italy
Tel: +39.081.19230606
Fax: +39.081.19230651
Email: banfi@tigem.it

Sabrina Carrella, PhD
TIGEM (Telethon Institute of Genetics and Medicine)
Via Campi Flegrei 34, 80078 Pozzuoli (Naples), Italy
Tel: +39 08119230655
Email: carrella@tigem.it

RESPONSE TO REVIEWERS

Referee #1

Major concerns.
(1) Mouse strains
R1: 1.1. The authors did not disclose the source and the specific line of P347S transgenic mice used in this paper. The original P347S transgenic mouse paper (PMID: 8943080) describes two lines, which exhibited distinct degenerate rates. Which specific line was used in this paper?

OUR REPLY: We thank Reviewer 1 for pointing out this important issue. The line we used is the A1 (Li et al., 1996). We obtained the line from the laboratory of Dr. Jane G. Farrar (Chadderton et al., 2009; Loscher et al., 2007; Palfi et al., 2016) via request of one of us (Prof. Enrico Maria Surace). The same line was previously reported by us in a number of publications, as also mentioned in line 387 of the current version of the manuscript (Botta et al., 2017, 2016; Karali et al., 2020; Claudio Mussolino et al., 2011; C Mussolino et al., 2011).
We apologize for not providing the exact source of the P347S line but this omission can be easily amended in a revised version of the manuscript.

R1:1.2. Importantly, P347S mice were on the background C57BL/6 (Charles River) (Line 387), and miR-181 deficient mice (on the background of C57BL/6 (National Cancer Institute). However, except for C57BL/6J (from Jackson Lab), C57BL/6 mice from almost all other vendors tested (including Charles River) are contaminated with the rd8 (retinal degeneration 8) allele (PMID: 22447858). The littermates with or without rd8 could have degenerated at a different rate. The authors should reveal the rd8 genotyping results to rule out the differential phenotypes that are contributed by the presence of the rd8 allele.

OUR REPLY: We believe that the *rd8* allele does not contribute to the phenotypic amelioration that we observe in our experiments for the following reasons:

1. As mentioned above, the P347S line used in this manuscript was obtained from Dr. Jane G. Farrar. This line is in the 129 S2/SvHsd (Harlan, Huntington, UK) background (Chadderton et al., 2009; Loscher et al., 2007; Palfi et al., 2016). The 129 S2/Sv background is wt for the *rd8* allele (Luhmann et al., 2015; Mattapallil et al., 2012). In the revised version of the manuscript, we will amend the material and methods section by providing further details on this line.

2. In our experimental setting, we used F1 P347S transgenic mice obtained by crossing F0 animals with C57BL/6J mice (see lines 286-387), as also previously described (Botta et al., 2017, 2016; Karali et al., 2020; Claudio Mussolino et al., 2011; C Mussolino et al., 2011). We want to point out that, for this purpose, we only used C57BL/6J mice that Charles Rivers delivered to us thanks to an agreement with the Jackson Labs (https://www.criver.com/products-services/find-model/jax-c57bl6j-mice?region=27). In a revised version of the manuscript, we will provide further details on the mouse strains used.

3. The *rd8* allele has been reported to cause typical *rd8* lesions to a variable extent, which were well developed as early as 3-6 weeks of age (Mattapallil et al., 2012; Mehalow, 2003). In a previous paper (Indrieri et al., 2019), we already demonstrated that the miR-181a/b$^{+/+}$ and the miR-181a/b$^{-/-}$ mice did not show any retinal abnormalities, as assessed by morphological and functional analyses (ERG), at 8 weeks of age.

As additional precaution, we also screen the acquired C57BL/6J mice for the possible presence of the *rd8* allele. Just as a representative example, the figure below shows the result of a Sanger sequencing analysis of the *Crb1* locus encompassing the *rd8* mutation carried out in a C57BL/6J male and a C57BL/6J female. The analysis reveals no evidence for the presence of the *Rd8* allele.

Based on the above considerations, we are confident that there is no *rd8* contamination whatsoever in the mouse litters we used for the AAV-mediated Sponge-miR-181a/b injection in the paper.

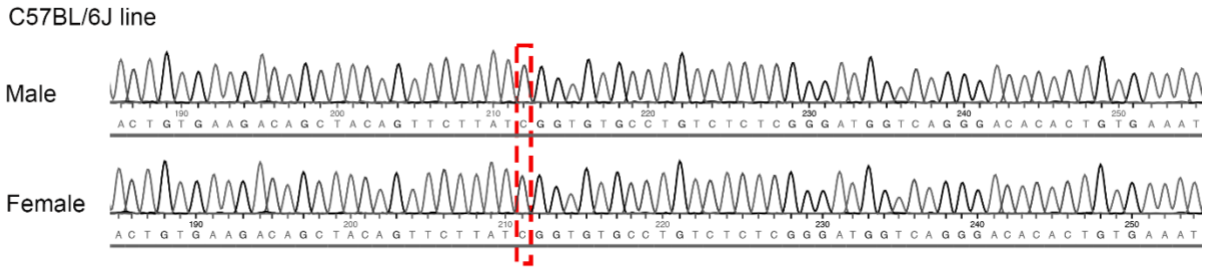

Moreover, we want to point out that, again in the case of Sponge-miR-181a/b experiments, animals are injected in one eye with AAV2/8.GFP.Sponge-miR-181a/b and with AAV2/8. GFP (control) in the contralateral eye (as reported in lines 211-212; and line 274). In this experimental setting the contribution of the genetic background is therefore minimized.

(2) Endpoints.

R1: 2.1. The original paper of P347S mice emphasized the rhodopsin immunostaining was indistinguishable from the WT controls (Figs. 2 and 3 in PMID: 8943080). So it is puzzling why the authors show the rhodopsin mislocalization in the P347S mice and used it as an endpoint (Fig. 1I-Jm Fig. 2A-D, Fig. 5)?

OUR REPLY: Indeed, this is not the first time that Rhodopsin mislocalization in the ONL has been reported in the P347S mouse line, by using anti-rhodopsin antibodies that recognise both murine WT RHO and human RHO [(Chadderton et al., 2009), Figure 6; (Patrizi et al., 2021), Figure 4; (Marrocco et al., 2021), (Figure 3)], as in the case of our paper (anti-Rhodopsin antibody used: Abcam, ab3267, see line 448).
Li and colleagues (Li et al., 1996) compared the distribution of human rhodopsin between P347S mice (C1 line) and the NHR, E line (a previously characterized transgenic mouse line overexpressing wild type human rhodopsin) at postnatal day 20 (please note that we do our analysis at later stages). They used the monoclonal antibody rho 3A6 (Hodges et al., 1988), which recognizes the human but not the murine rhodopsin, observing no differences between the wt and mutant human rhodopsin distribution (as correctly underlined by the reviewer 1).
Therefore, based on all of the above considerations, we do not believe that our data are in contrast with previous reports
In the revised version of the manuscript, we will add further details on the specificity of the anti-Rhodopsin antibody used also in the Results, for the sake of clarity.

R1: 2.2. The other readouts used, such as the thickness of the photoreceptor nuclear layer (ONL), the length of the inner segment (by F-actin and the lengths of the outer segment (by TEM) highly subject to the sectioning planes and the survey areas. Both of this and the single data point cannot convincingly demonstrate the restoration of the photoreceptor structures.

OUR REPLY: The count of nuclei in the ONL, the evaluation of the ONL thickness and the analysis of photoreceptor markers by immunofluorescence, and active-Casp3 and TUNEL (reported in figure 1,

figure 2, figure 5, figure 6, Supplementary figure 1, Supplementary figure 5) were performed only on sections that contain the optic nerve and in comparable regions with respect to the distance from the optic nerve (as reported in lines 465-472, line 441, and line 459). Such kind of selection ensures the characterization of comparable planes and areas. The OS length was analysed on at least 10 images per eye, for a total number of OS measured of around 200 for the P347S and 150 for the P347S/miR-181a/b-$1^{+/-}$. Only tissue sections in which the sagittal orientation of the connecting cilium (cc) was visible were chosen, as shown in Figure Appendix 1, ensuring the correct sectioning of the eyes (as we reported in lines 475-478).

R1: 2.3. The mitochondrial populations in photoreceptors are highly heterogeneous, their shape and sizes are dependent on the distance to the outer segment, the time of the harvest, etc. Thus, it is not reliable to depict the size based on the single TEM images, as used by this communication without rigorous experimental description (Fig. 3B-F). Recent investigators have resorted to 3D scanning EM instead due to the problems (e.g., PMID: 34552195; PMID: 33144507).

OUR REPLY: We are aware of the highly heterogeneous shape and sizes of mitochondria in photoreceptors. Indeed, to avoid daily changes that occur in photoreceptor mitochondria [reported in zebrafish cone photoreceptors, (Giarmarco et al., 2020)] we carried out TEM on retinal samples derived from animals of the same litter (as mentioned in lines 390-391) whose eyes were collected all together at the same time of the day. We analyzed mitochondrial number, perimeter and area on several TEM images derived from 2 eyes/genotypes, for a total number of images analyzed of 22/genotype. Only tissue sections in which the sagittal orientation of the connecting cilium (cc) was visible were chosen, as shown in Figure Appendix 1, ensuring the correct sectioning of the eyes (as we report in lines 528-529). The mitochondrial characterization was performed for all mitochondria located within the entire inner segment (IS), from the outer limiting membrane to the outer segment (OS). Our data clearly show that P347S mitochondria are fragmented and that the changes in shape and size that we report are independent from localization within the IS. The same is evident for P347S/miR-$181^{+/-}$ mitochondria that show an amelioration of their morphology. Representative images showing the region of interest are reported below (scale bars are 2 µm). This experimental methodology has been already reported in other publications (Anastasia et al., 2021; Costa et al., 2010; Indrieri et al., 2019; Mirra et al., 2021; Naon et al., 2016; Ozaki et al., 2020; Quintana-Cabrera et al., 2018; Tsakiri et al., 2019; Xia et al., 2019). Nevertheless, we can also add, in the revised version of the manuscript, additional analysis of mitochondria morphology such as immunofluorescence with mitochondrial markers, to further corroborate the above phenotypes.

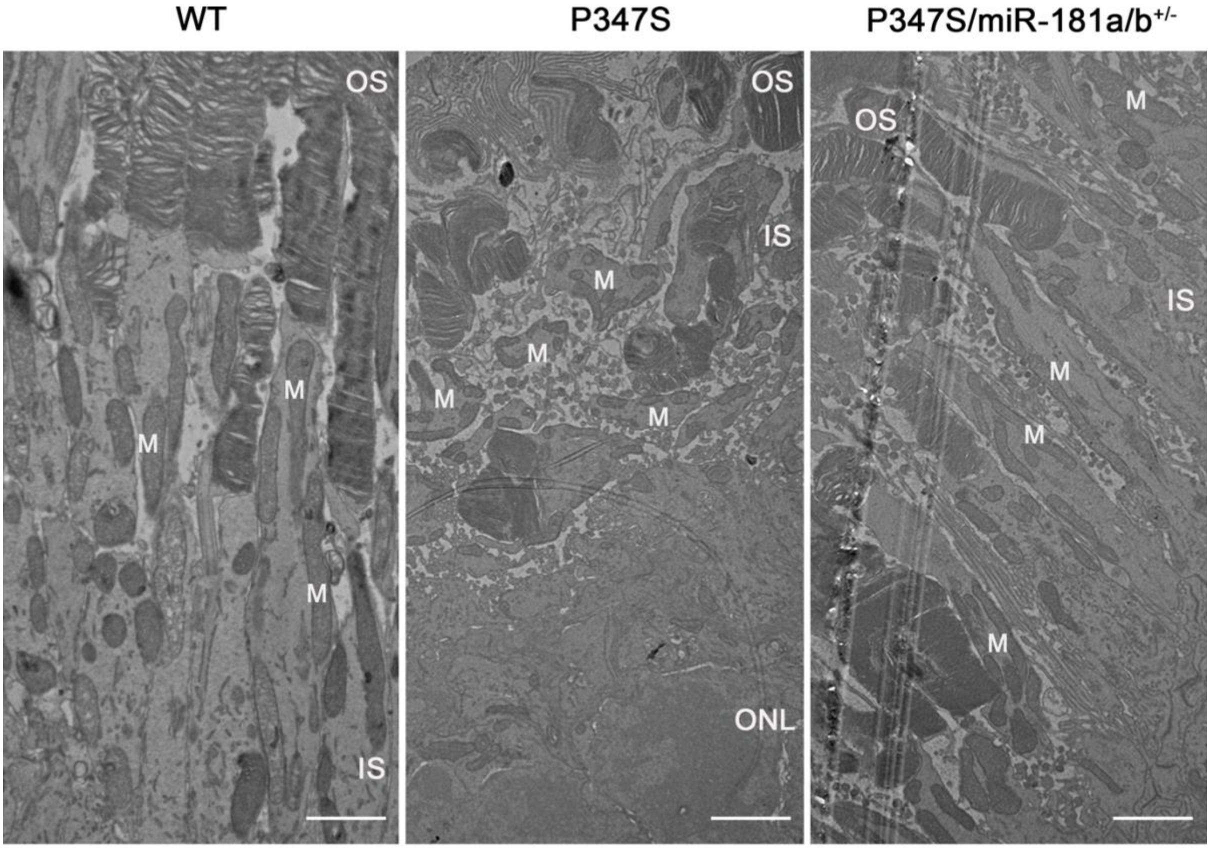

R1: The Scale Bars of all figures, including EM, are missing.

OUR REPLY: We strongly apologize for this inaccuracy. Unfortunately, this omission originates from a technical problem that occurred during the conversion in pdf of the originally uploaded files on the journal website. Indeed, all scale bars are present in the full size .tif versions uploaded. Nevertheless, we apologize again for not realizing about this problem in the generated pdf file. Obviously, this omission is easily amendable in a revised version of the manuscript.

R1: 2.4. The time point for the mitochondria characterization was when the photoreceptors already look dead (Fig. 3C). Why not be assayed at PN12 to match the transcriptome analysis and to directly address the mitochondria atrophy is an "early" event, i.e., a cause vs. consequence of the cell death.

OUR REPLY: We chose to perform most of our "morphological" phenotypic analysis (including mitochondria characterization) starting at p30 because we wanted to correlate functional rescue with changes in morphology of retinal structure following miR-181a/b downregulation. Since p30 is the first suitable and reliable time point for ERG analysis, we decided to begin all the phenotypic analysis at that stage to address the miR-181a/b downregulation effect at the same time point. In any case, the Reviewer's request is well taken and to address it, we will add additional immunofluorescence assays for mitochondrial markers at p12 in the revised version of the manuscript.

R1: 2.5. Scotopic ERG a-wave represents photoreceptor functions. Several P347S mouse lines still have detectable a-wave (PMID: 8943080). It is curious why the a-wave results were not shown, but only the b-wave data were shown. Since b-wave represents the inner retinal neuron function, the positive results cannot directly conclude the improvement of photoreceptor function. Please show the Scotopic ERG

a-wave (taken at different light intensity illumination) of both mutant and WT control mice. The latter control will help to register the extent of the functional restoration relative to the WT mice.

OUR REPLY: We have registered the a-waves from the same group of animals at the same time points for which the b-waves are shown. We will add this information in the revised version of the manuscript.

R1: 2.6. The authors showed the PNA staining is better preserved in the double transgenic mice (Fig. 1). Since PNA was typically used to highlight the cone outer segments, does the miR-181a/b deficiency improve the cone photopic ERG (a and b waves)?

OUR REPLY: As already stated in our response to the previous point, we will add the a-wave responses, both scotopic and photopic, in the revised version of the manuscript. The cone photopic ERG b-wave results are already shown in in figure 1H and figure 2F of the current version of the manuscript.

R1: 2.7. To claim for the cell-protective role of miR181a/b, one might have to show the slow-down of the photoreceptor cell death or prevent cell death for early intervention. These are not the case in the current presentation.

OUR REPLY: In figure 1B-G we showed images and quantification of TUNEL assay and Active-Casp3 immunofluorescence staining, highlighting that miR-181a/b downregulation significantly decreases the extent of cell death in Outer nuclear layer (ONL).

R1: Fig. 2E shows the ONL thickness was increased from 5 to 6 uM. The minor, albeit "statistically significant", the increase was unlikely to be physiologically relevant, considering the WT ONL is up to 50-um thick.

OUR REPLY: Figure 2 shows data obtained at p90, an advanced time point of retinal degeneration in the P347S animal model, with only a single row of photoreceptor nuclei. Although the small increase is far from that present in the wild type condition, nevertheless it underlies an amelioration of retinal function (as assessed by ERG) thus supporting its physiological relevance and ability to slow-down the degeneration of this severe and dominant form of RP, for which at present no treatments are available.

R1: The ONL thickness is region-dependent (~50um for central, and ~25um for periphery). Unfortunately, the regional variation was not considered in any of the ONL thickness quantification (Fig. 2E, Fig. EV 1C). Hence, calling miR-181a/b deficiency has a protective role (Lines 253, 262, 772, ...etc.) is an overstatement.

OUR REPLY: As also stated above, the ONL thickness and the analysis of photoreceptor markers by immunofluorescence, were performed only on sections in which the optic nerve was contained and in comparable regions with respect to the distance from the optic nerve (as reported in lines 465-472, line 441 and line 459). Such kind of selection ensures the characterization of comparable planes and areas of the retina for the analyses of the different genotypes or sponge treatments.

R1: 2.8. Although the cell-type specificity was well demonstrated for the AAV studies, only a small region was shown. Please show the low-magnification view of the entire retina globe for the overall infection rate. Were the eyes with injection-related trauma been excluded for consideration? This information is useful for the consideration of the ERG results, which should be carried out using the criteria as described above.

OUR REPLY: In the figure below, we provide an example of retinas injected with AAV2/8.CMV.GFP-Sponge-miR-181a/b and with AAV2/8.CMV.GFP (control) in the contralateral eye, analyzed by fundoscopy. Only sections with comparable GFP expression levels were compared. We indeed excluded from our analysis samples that showed sign of injection-related damages, or eyes that turned out to be not properly injected as assessed from GFP expression analysis.
We will add this information in the materials and methods section of the revised version of the manuscript.

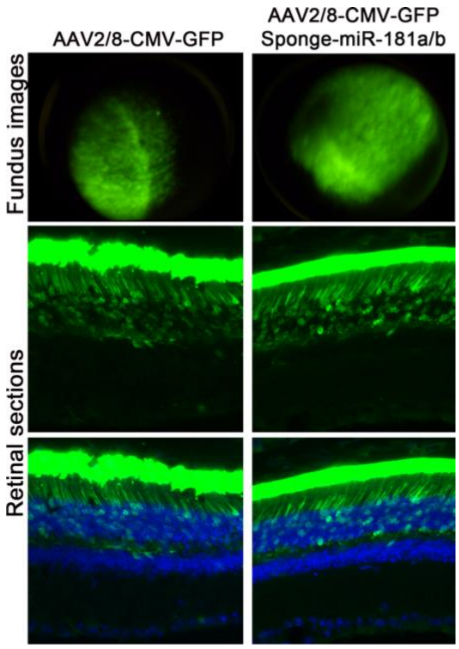

R1: 2.9. The authors used either "eyes" or "tissues" in describing the procedures for RNA isolation (qPCR, transcriptome) and protein isolation (Lines 396, 399, 417, 790). What tissues? Were the whole eyes (containing many cell types including the lens, cornea, etc.) were used? The changes in the expression of the miR181a/b targets or mitochondria functions of the mixed cell population do not necessarily suggest that these genes are changed in the photoreceptors. Can the authors demonstrate any of the proteins were changed in the photoreceptor cell layers by immunostaining?

OUR REPLY: We extracted RNA or proteins from optic cup. We will add more precise information about this issue in the revised version of the manuscript. Moreover, we will also provide immunofluorescence or immunostaining analysis of miR-181a/b modulated proteins in retinal sections.

R1: 3. Others

3.1. The sources of the mouse lines should be acknowledged.

3.2. Methods should be written at a detailed level that others can reproduce.

3.3. The vectors, the helpers, the cell systems, and the purification of AAV should be detailed.

OUR REPLY: We will fix all of the above points

3.4. Erroneous statement or overstatement.

Since only correlative data was provided, LINE 109. "We also observed amelioration of mitochondria morphology in PRs, which is mediated by regulation of the mitochondria fission protein Dra1, mainly via the activation of the Jak2/Stat3 pathway" is an overstatement.

OUR REPLY: We will tune down the above statement

Line 60. IRD represents the most important cause of vision impairment in the Western population." is an erroneous statement.

OUR REPLY: We agree with the Reviewer. This is an erroneous statement: we will certainly revise it.

LINE 101-103 is an erroneous statement. There is no experimental data that supports that mitochondria dysfunction is an "early event" of photoreceptor cell death in P347S rhodopsin mutation. The transcriptome/qPCR/proteins data were obtained from multiple eye cell types. The mitochondria characterization of photoreceptors was carried out at the time point when the photoreceptors were severely atrophic (see above).

Discussion should be grossly truncated and focus on discussing the experimental results. Speculation should be stated so whenever it is complying.

OUR REPLY: We are very thankful to the reviewer 1 for his/her accurate revision of the manuscript, and we will implement the above requests.

Referee #2

Minor improvements:
Results
R 2: As a general critique suggest including the number of animals and the age of the animals in each figure to clearly identify the variabilities in each data set.

OUR REPLY: We are very thankful to the reviewer, and we will add this information in the revised version of the manuscript

R 2: Figure 1:
1. Only A wave responses are shown in figure 1F. Strongly suggest showing representative images of the ERG traces and also include B wave data as well.
2. Optical densitometry quantification of the microscopy images to quantify the differences in intensity and locations of the various retinal stains will strengthen this important data.

OUR REPLY: We have registered both a-waves and b-waves for all the time points, and we will add both data in the revised version of the manuscript. Optical densitometry quantification can be easily performed on our images of immunostained retina samples. We will add this information in the revised version of the manuscript.

R 2: Figure 2:
1. For consistency in the P90 aged animals please repeat all the same immunohistochemistry straining as per figure 1. This would strengthen the manuscript by showing and comparing the differences between P30 and P90 days.
2. Once again please show the quantification of microscopy images and also the ERG B wave data and representative EGRs as well.

OUR REPLY: We will add this information in the revised version of the manuscript.

R 2: Figure 3:
1. Further context to the RNAseq data is required. What analysis was performed and while it is mentioned this is a reanalysis, further detail in the figure should be provided. Perhaps a table of associated genes used for the analysis would be beneficial, or a list of genes associated with the specific pathways identified.
2. This is true for all figures but especially for figure 3 E and F. It is difficult to see which is the P values associated with each comparison. Suggest either use lines or * to indicate the significance as well as which are the comparisons being explored.
3. While not significant I would suggest including the data of the mitochondrial number in this section. It is good data to show that there is no change in number, but a change in their morphology. This data's inclusion would help make this point more clearly.

OUR REPLY: We will add this information and implement the suggested changes in the revised version of the manuscript.

R 2: Figure 4:
1. The WB data is not particularly strong. Especially the GAPDH staining which has not provided a great reference level. Particularly for the P347S values which appear significantly stronger than other parts of the gel. Authors should comment on this or redo this analysis.
2. All WB data should be shown. At present only representative images for Drp1 are provided. Please provide this data for all other WB performed.

OUR REPLY: In our WB experiments we used several control references, not only GAPDH but also p115 (see figure 4, Supplementary figure 2 and Supplementary figure 3). We will add additional reference markers as required. All the WB data are shown, not only the representative image for Drp1, but all the WB of the other protein mentioned (OXPHOS, CS, Opa1, Mfn1 and 2, Fis1, STAT3 and phosppho-STAT3) are reported in supplementary figure 2 and supplementary figure 3. In addition, in the appendix file, we already reported all the uncut WB data (original blots), for all the WB shown in the manuscript.

R 2: Figure 5:
1. Again further ERG data is required and the representative images. Also there is no control ERG shown as a baseline reading. This should be included.
2. The GFP labelling is not shown anywhere in the main images, so it is unclear if the AAV transfection was successful (Note some are placed

in the supplementary). Quantification and qualitative data is required to validate these findings and this should all be included in the main results not in the supplementary section.

OUR REPLY: We will add this information in the revised version of the manuscript.

R 2: Figure 6:
1. Justification of the different functional tests are required. Did the ERGs not show a positive result? Suggest consistency of tests across the experiments reported in this manuscript.

OUR REPLY: We analyzed the ERG of *rd10* injected animals at p30. Although, there is a trend towards improvement after AAV2/8-CMV-GFP.miR-181a/b sponge treatment (see figure below), we did not observe any statistical significance of these data, probably due to the severely impaired ERG response and a dramatically reduced ONL thickness at p30 in this mouse model (Chang et al., 2002). Therefore, we decided to use visual functional assays that allow to highlight even slight differences in retinal functionality, such as pupillary light responses (PLR) and Water Maze tests (as mentioned in lines 279-280). However, in the revised version of the manuscript we will better clarify this point and add these data.

p4 injected *rd10*

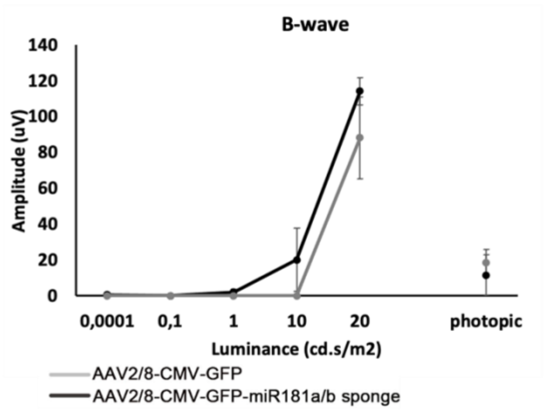

p10 injected *rd10*

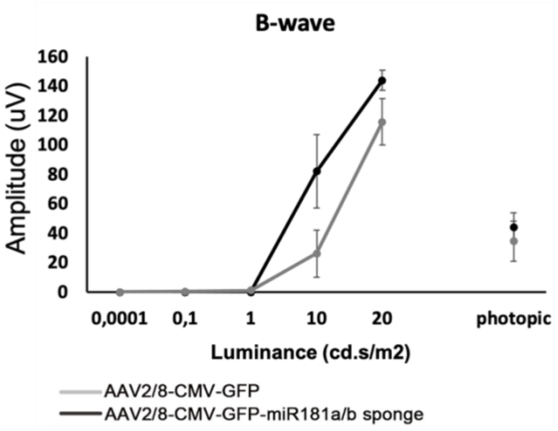

Referee #3

Comments to authors,

R 3: 1. Please add scale for all photos of tissue sections in this manuscript.

OUR REPLY: We are very thankful to the reviewers for highlighting the lack of Scale Bars. As already mentioned in the response to reviewer 1, this omission originates from a technical problem that occurred during the conversion in pdf of the originally uploaded files on the journal website. Indeed, all scale bars are present in the full size .tif versions uploaded. Nevertheless, we apologize again for not realizing

about this problem in the generated pdf file. Obviously, this omission is easily amendable in a revised version of the manuscript.

R 3: 2. Fig.1: Please show photo of full retina to observe full structure of the photoreceptor and other area of retina.

OUR REPLY: We will add this information in the revised version of the manuscript.

R 3: 3. Fig.3B: Please add scale and organelle name in the photo.

OUR REPLY: See previous answer, we will add organelle name in photo

R 3: 4. Fig.5 & Fig.6: Additional immunostaining of endogenous photoreceptor makers is needed to assess the condition of the retina. Normal mouse section should be added for this assessment and the entire retina should be shown. Mitochondria staining should be also added to see how much is remaining compare to normal retina and non-treated disease mice.

OUR REPLY: We will add this information in the revised version of the manuscript.

References:

- Anastasia, I., Ilacqua, N., Raimondi, A., Lemieux, P., Ghandehari-Alavijeh, R., Faure, G., Mekhedov, S.L., Williams, K.J., Caicci, F., Valle, G., Giacomello, M., Quiroga, A.D., Lehner, R., Miksis, M.J., Toth, K., de Aguiar Vallim, T.Q., Koonin, E.V., Scorrano, L., Pellegrini, L., 2021. Mitochondria-rough-ER contacts in the liver regulate systemic lipid homeostasis. Cell Reports 34, 108873. https://doi.org/10.1016/j.celrep.2021.108873
- Botta, S., de Prisco, N., Marrocco, E., Renda, M., Sofia, M., Curion, F., Bacci, M.L., Ventrella, D., Wilson, C., Gesualdo, C., Rossi, S., Simonelli, F., Surace, E.M., 2017. Targeting and silencing of rhodopsin by ectopic expression of the transcription factor KLF15. JCI Insight 2, e96560. https://doi.org/10.1172/jci.insight.96560
- Botta, S., Marrocco, E., de Prisco, N., Curion, F., Renda, M., Sofia, M., Lupo, M., Carissimo, A., Bacci, M.L., Gesualdo, C., Rossi, S., Simonelli, F., Surace, E.M., 2016. Rhodopsin targeted transcriptional silencing by DNA-binding. eLife 5, e12242. https://doi.org/10.7554/eLife.12242
- Chadderton, N., Millington-Ward, S., Palfi, A., O'Reilly, M., Tuohy, G., Humphries, M.M., Li, T., Humphries, P., Kenna, P.F., Farrar, G.J., 2009. Improved Retinal Function in a Mouse Model of Dominant Retinitis Pigmentosa Following AAV-delivered Gene Therapy. Molecular Therapy 17, 593–599. https://doi.org/10.1038/mt.2008.301
- Chang, B., Hawes, N.L., Hurd, R.E., Davisson, M.T., Nusinowitz, S., Heckenlively, J.R., 2002. Retinal degeneration mutants in the mouse. Vision Research 42, 517–525. https://doi.org/10.1016/S0042-6989(01)00146-8
- Costa, V., Giacomello, M., Hudec, R., Lopreiato, R., Ermak, G., Lim, D., Malorni, W., Davies, K.J.A., Carafoli, E., Scorrano, L., 2010. Mitochondrial fission and cristae disruption increase the response of cell models of Huntington's disease to apoptotic stimuli. EMBO Mol Med 2, 490–503. https://doi.org/10.1002/emmm.201000102

- Giarmarco, M.M., Brock, D.C., Robbings, B.M., Cleghorn, W.M., Tsantilas, K.A., Kuch, K.C., Ge, W., Rutter, K.M., Parker, E.D., Hurley, J.B., Brockerhoff, S.E., 2020. Daily mitochondrial dynamics in cone photoreceptors. Proc Natl Acad Sci U S A 117, 28816–28827. https://doi.org/10.1073/pnas.2007827117
- Hodges, R.S., Heaton, R.J., Parker, J.M., Molday, L., Molday, R.S., 1988. Antigen-antibody interaction. Synthetic peptides define linear antigenic determinants recognized by monoclonal antibodies directed to the cytoplasmic carboxyl terminus of rhodopsin. J Biol Chem 263, 11768–11775.
- Indrieri, A., Carrella, S., Romano, A., Spaziano, A., Marrocco, E., Fernandez-Vizarra, E., Barbato, S., Pizzo, M., Ezhova, Y., Golia, F.M., Ciampi, L., Tammaro, R., Henao-Mejia, J., Williams, A., Flavell, R.A., De Leonibus, E., Zeviani, M., Surace, E.M., Banfi, S., Franco, B., 2019. miR-181a/b downregulation exerts a protective action on mitochondrial disease models. EMBO Mol Med 11, e8734. https://doi.org/10.15252/emmm.201708734
- Karali, M., Guadagnino, I., Marrocco, E., De Cegli, R., Carissimo, A., Pizzo, M., Casarosa, S., Conte, I., Surace, E.M., Banfi, S., 2020. AAV-miR-204 Protects from Retinal Degeneration by Attenuation of Microglia Activation and Photoreceptor Cell Death. Molecular Therapy - Nucleic Acids 19, 144–156. https://doi.org/10.1016/j.omtn.2019.11.005
- Li, T., Snyder, W.K., Olsson, J.E., Dryja, T.P., 1996. Transgenic mice carrying the dominant rhodopsin mutation P347S: evidence for defective vectorial transport of rhodopsin to the outer segments. Proc Natl Acad Sci U S A 93, 14176–14181. https://doi.org/10.1073/pnas.93.24.14176
- Loscher, C.J., Hokamp, K., Kenna, P.F., Ivens, A.C., Humphries, P., Palfi, A., Farrar, G.J., 2007. Altered retinal microRNA expression profile in a mouse model of retinitis pigmentosa. Genome Biol 8, R248. https://doi.org/10.1186/gb-2007-8-11-r248
- Luhmann, U.F.O., Carvalho, L.S., Holthaus, S.-M. kleine, Cowing, J.A., Greenaway, S., Chu, C.J., Herrmann, P., Smith, A.J., Munro, P.M.G., Potter, P., Bainbridge, J.W.B., Ali, R.R., 2015. The severity of retinal pathology in homozygous Crb1rd8/rd8 mice is dependent on additional genetic factors. Human Molecular Genetics 24, 128–141. https://doi.org/10.1093/hmg/ddu424
- Marrocco, E., Maritato, R., Botta, S., Esposito, M., Surace, E.M., 2021. Challenging Safety and Efficacy of Retinal Gene Therapies by Retinogenesis. IJMS 22, 5767. https://doi.org/10.3390/ijms22115767
- Mattapallil, M.J., Wawrousek, E.F., Chan, C.-C., Zhao, H., Roychoudhury, J., Ferguson, T.A., Caspi, R.R., 2012. The Rd8 mutation of the Crb1 gene is present in vendor lines of C57BL/6N mice and embryonic stem cells, and confounds ocular induced mutant phenotypes. Invest Ophthalmol Vis Sci 53, 2921–2927. https://doi.org/10.1167/iovs.12-9662
- Mehalow, A.K., 2003. CRB1 is essential for external limiting membrane integrity and photoreceptor morphogenesis in the mammalian retina. Human Molecular Genetics 12, 2179–2189. https://doi.org/10.1093/hmg/ddg232
- Mirra, S., García-Arroyo, R., B. Domènech, E., Gavaldà-Navarro, A., Herrera-Úbeda, C., Oliva, C., Garcia-Fernàndez, J., Artuch, R., Villarroya, F., Marfany, G., 2021. CERKL, a retinal dystrophy gene, regulates mitochondrial function and dynamics in the mammalian retina. Neurobiology of Disease 156, 105405. https://doi.org/10.1016/j.nbd.2021.105405
- Mussolino, C, della Corte, M., Rossi, S., Viola, F., Di Vicino, U., Marrocco, E., Neglia, S., Doria, M., Testa, F., Giovannoni, R., Crasta, M., Giunti, M., Villani, E., Lavitrano, M., Bacci, M.L., Ratiglia, R., Simonelli, F., Auricchio, A., Surace, E.M., 2011. AAV-mediated photoreceptor transduction of the pig cone-enriched retina. Gene Ther 18, 637–645. https://doi.org/10.1038/gt.2011.3

- Mussolino, Claudio, Sanges, D., Marrocco, E., Bonetti, C., Di Vicino, U., Marigo, V., Auricchio, A., Meroni, G., Surace, E.M., 2011. Zinc-finger-based transcriptional repression of rhodopsin in a model of dominant retinitis pigmentosa. EMBO Mol Med 3, 118–128. https://doi.org/10.1002/emmm.201000119
- Naon, D., Zaninello, M., Giacomello, M., Varanita, T., Grespi, F., Lakshminaranayan, S., Serafini, A., Semenzato, M., Herkenne, S., Hernández-Alvarez, M.I., Zorzano, A., De Stefani, D., Dorn, G.W., Scorrano, L., 2016. Critical reappraisal confirms that Mitofusin 2 is an endoplasmic reticulum–mitochondria tether. Proc. Natl. Acad. Sci. U.S.A. 113, 11249–11254. https://doi.org/10.1073/pnas.1606786113
- Ozaki, T., Utsumi, S., Iwamoto, T., Tanaka, M., Tomita, H., Sugano, E., Ishiyama, E., Ishida, K., 2020. Data on mitochondrial ultrastructure of photoreceptors in pig, rabbit, and mouse retinas. Data in Brief 30, 105544. https://doi.org/10.1016/j.dib.2020.105544
- Palfi, A., Hokamp, K., Hauck, S.M., Vencken, S., Millington-Ward, S., Chadderton, N., Carrigan, M., Kortvely, E., Greene, C.M., Kenna, P.F., Farrar, G.J., 2016. microRNA regulatory circuits in a mouse model of inherited retinal degeneration. Sci Rep 6, 31431. https://doi.org/10.1038/srep31431
- Patrizi, C., Llado, M., Benati, D., Iodice, C., Marrocco, E., Guarascio, R., Surace, E.M., Cheetham, M.E., Auricchio, A., Recchia, A., 2021. Allele-specific editing ameliorates dominant retinitis pigmentosa in a transgenic mouse model. The American Journal of Human Genetics 108, 295–308. https://doi.org/10.1016/j.ajhg.2021.01.006
- Quintana-Cabrera, R., Quirin, C., Glytsou, C., Corrado, M., Urbani, A., Pellattiero, A., Calvo, E., Vázquez, J., Enríquez, J.A., Gerle, C., Soriano, M.E., Bernardi, P., Scorrano, L., 2018. The cristae modulator Optic atrophy 1 requires mitochondrial ATP synthase oligomers to safeguard mitochondrial function. Nat Commun 9, 3399. https://doi.org/10.1038/s41467-018-05655-x
- Tsakiri, E.N., Gumeni, S., Vougas, K., Pendin, D., Papassideri, I., Daga, A., Gorgoulis, V., Juhász, G., Scorrano, L., Trougakos, I.P., 2019. Proteasome dysfunction induces excessive proteome instability and loss of mitostasis that can be mitigated by enhancing mitochondrial fusion or autophagy. Autophagy 15, 1757–1773. https://doi.org/10.1080/15548627.2019.1596477
- Xia, H., Hu, Q., Li, L., Tang, X., Zou, J., Huang, L., Li, X., 2019. Protective effects of autophagy against blue light-induced retinal degeneration in aged mice. Sci. China Life Sci. 62, 244–256. https://doi.org/10.1007/s11427-018-9357-y

25th Mar 2022

Dear Dr. Carrella,

Thank you for your letter and request to reconsider our decision on your manuscript, and please accept my apologies for the delay in getting back to you.
I have now read your appeal and discussed it with my colleagues.
We would be willing to consider a revised version of your manuscript, provided that you address the points raised by the reviewers in full (in particular the concerns from referee #1 regarding the models used and endpoints analyzed).

Please attach a covering letter giving details of the way in which you have handled each of the points raised by the referees. A revised manuscript will once again be subject to review and we cannot guarantee at this stage that the eventual outcome will be favorable.

I look forward to seeing a revised form of your manuscript as soon as possible.

Please use this link to login to the manuscript system and submit your revision: https://embomolmed.msubmit.net/cgi-bin/main.plex

Yours sincerely,

Lise Roth

Lise Roth, Ph.D
Scientific Editor
EMBO Molecular Medicine

*****

When submitting your revised manuscript, please carefully review the instructions that follow below. We perform an initial quality control of all revised manuscripts before re-review; failure to include requested items will delay the evaluation of your revision.

We require:

1) A .docx formatted version of the manuscript text (including legends for main figures, EV figures and tables). Please make sure that the changes are highlighted to be clearly visible.

2) Individual production quality figure files as .eps, .tif, .jpg (one file per figure). For guidance, download the 'Figure Guide PDF': (https://www.embopress.org/page/journal/17574684/authorguide#figureformat).

3) A .docx formatted letter INCLUDING the reviewers' reports and your detailed point-by-point responses to their comments. As part of the EMBO Press transparent editorial process, the point-by-point response is part of the Review Process File (RPF), which will be published alongside your paper.

4) A complete author checklist, which you can download from our author guidelines (https://www.embopress.org/page/journal/17574684/authorguide#submissionofrevisions). Please insert information in the checklist that is also reflected in the manuscript. The completed author checklist will also be part of the RPF.

5) Please note that all corresponding authors are required to supply an ORCID ID for their name upon submission of a revised manuscript.

6) It is mandatory to include a 'Data Availability' section after the Materials and Methods. Before submitting your revision, primary datasets produced in this study need to be deposited in an appropriate public database, and the accession numbers and database listed under 'Data Availability'. Please remember to provide a reviewer password if the datasets are not yet public (see https://www.embopress.org/page/journal/17574684/authorguide#dataavailability).

In case you have no data that requires deposition in a public database, please state so in this section. Note that the Data Availability Section is restricted to new primary data that are part of this study.

7) For data quantification: please specify the name of the statistical test used to generate error bars and P values, the number

(n) of independent experiments (specify technical or biological replicates) underlying each data point and the test used to calculate p-values in each figure legend. The figure legends should contain a basic description of n, P and the test applied. Graphs must include a description of the bars and the error bars (s.d., s.e.m.). See also 'Figure Legend' guidelines: https://www.embopress.org/page/journal/17574684/authorguide#figureformat

8) We would also encourage you to include the source data for figure panels that show essential data. Numerical data should be provided as individual .xls or .csv files (including a tab describing the data). For blots or microscopy, uncropped images should be submitted (using a zip archive if multiple images need to be supplied for one panel). Additional information on source data and instruction on how to label the files are available at

.

9) Our journal encourages inclusion of *data citations in the reference list* to directly cite datasets that were re-used and obtained from public databases. Data citations in the article text are distinct from normal bibliographical citations and should directly link to the database records from which the data can be accessed. In the main text, data citations are formatted as follows: "Data ref: Smith et al, 2001" or "Data ref: NCBI Sequence Read Archive PRJNA342805, 2017". In the Reference list, data citations must be labeled with "[DATASET]". A data reference must provide the database name, accession number/identifiers and a resolvable link to the landing page from which the data can be accessed at the end of the reference. Further instructions are available at .

10) We replaced Supplementary Information with Expanded View (EV) Figures and Tables that are collapsible/expandable online. A maximum of 5 EV Figures can be typeset. EV Figures should be cited as 'Figure EV1, Figure EV2" etc... in the text and their respective legends should be included in the main text after the legends of regular figures.

- For the figures that you do NOT wish to display as Expanded View figures, they should be bundled together with their legends in a single PDF file called *Appendix*, which should start with a short Table of Content. Appendix figures should be referred to in the main text as: "Appendix Figure S1, Appendix Figure S2" etc.

- Additional Tables/Datasets should be labeled and referred to as Table EV1, Dataset EV1, etc. Legends have to be provided in a separate tab in case of .xls files. Alternatively, the legend can be supplied as a separate text file (README) and zipped together with the Table/Dataset file.

See detailed instructions here:

.

11) The paper explained: EMBO Molecular Medicine articles are accompanied by a summary of the articles to emphasize the major findings in the paper and their medical implications for the non-specialist reader. Please provide a draft summary of your article highlighting

- the medical issue you are addressing,

- the results obtained and

- their clinical impact.

This may be edited to ensure that readers understand the significance and context of the research. Please refer to any of our published articles for an example.

12) For more information: There is space at the end of each article to list relevant web links for further consultation by our readers. Could you identify some relevant ones and provide such information as well? Some examples are patient associations, relevant databases, OMIM/proteins/genes links, author's websites, etc...

13) Author contributions: the contribution of every author must be detailed in a separate section (before the acknowledgments).

14) A Conflict of Interest statement should be provided in the main text.

15) Every published paper now includes a 'Synopsis' to further enhance discoverability. Synopses are displayed on the journal webpage and are freely accessible to all readers. They include a short stand first (maximum of 300 characters, including space) as well as 2-5 one-sentences bullet points that summarizes the paper. Please write the bullet points to summarize the key NEW findings. They should be designed to be complementary to the abstract - i.e. not repeat the same text. We encourage inclusion of key acronyms and quantitative information (maximum of 30 words / bullet point). Please use the passive voice. Please attach these in a separate file or send them by email, we will incorporate them accordingly.

Please also suggest a striking image or visual abstract to illustrate your article as a PNG file 550 px wide x 300-600 px high.

EMBO Molecular Medicine has a "scooping protection" policy, whereby similar findings that are published by others during review or revision are not a criterion for rejection. Should you decide to submit a revised version, I do ask that you get in touch after three months if you have not completed it, to update us on the status.

Referee #1 (Comments on Novelty/Model System for Author):

(1) Mouse strains

1.1. The authors did not disclose the source and the specific line of P347S transgenic mice used in this paper. The original P347S transgenic mouse paper (PMID: 8943080) describes two lines, which exhibited distinct degenerate rates. Which specific line was used in this paper?

OUR REPLY: see our reply below (Major concerns, point 1.1)

1.2. Importantly, P347S mice were on the background C57BL/6 (Charles River) (Line 387), and miR-181 deficient mice (on the background of C57BL/6 (National Cancer Institute). However, except for C57BL/6J (from Jackson Lab), C57BL/6 mice from almost all other vendors tested (including Charles River) are contaminated with the rd8 (retinal degeneration 8) allele (PMID: 22447858). The littermates with or without rd8 could have degenerated at a different rate. The authors should reveal the rd8 genotyping results to rule out the differential phenotypes that are contributed by the presence of the rd8 allele.

OUR REPLY: see our reply below (Major concerns, point 1.2)

Referee #1 (Remarks for Author):

Previously, these authors suggested the inactivation of miR-181a/b can ameliorate the disease phenotype in Leber's hereditary optic neuropathy mouse models. In the current communication, this author sought to test the hypothesis that miR-181a/b deficiency can protect photoreceptors in retinal degeneration mouse models. It remains challenging to treat inherited retinal degeneration (IRD) diseases due to a wide range of genetic mutations. Both the transgenic and AAV injection approaches were undertaken. P347S rhodopsin mutant mouse line was tested by both methods. Rd10 mice were tested by the latter method. Several "endpoints" were used singly or together, including immunostaining photoreceptor proteins and dark ERG b-wave. Additional corrective evidence linking the compromised mitochondria functional pathway in the disease and rescue was provided. These results prompted the authors to conclude that miR-181a/b deficiency can provide a beneficial effect in treating multiple types of retinal degeneration diseases. As detailed below, the current presentation lacks rigor in the background, experimental designs, data quality, and data interpretation. Despite the importance of the subject investigated, several technical concerns fall short in concluding the miR-181a/b deficiency has a cell-protective role of photoreceptor cells in retinal degeneration mouse models.

Major concerns.

(1) Mouse strains

1.1. The authors did not disclose the source and the specific line of P347S transgenic mice used in this paper. The original P347S transgenic mouse paper (PMID: 8943080) describes two lines, which exhibited distinct degenerate rates. Which specific line was used in this paper?

OUR REPLY: We thank Reviewer 1 for pointing out this important issue. The line we used is the A1 (Li et al., 1996). We obtained the line from the laboratory of Dr. Jane G. Farrar (Chadderton et al., 2009; Loscher et al., 2007; Palfi et al., 2016) via request of one of the co-authors (Prof. Enrico Maria Surace). We have added this information in the materials and methods section (lines: 423 and 425-427) in the revised version of the manuscript.

1.2. Importantly, P347S mice were on the background C57BL/6 (Charles River) (Line 387), and miR-181 deficient mice (on the background of C57BL/6 (National Cancer Institute). However, except for C57BL/6J (from Jackson Lab), C57BL/6 mice from almost all other vendors tested (including Charles River) are contaminated with the rd8 (retinal degeneration 8) allele (PMID: 22447858). The littermates with or without rd8 could have degenerated at a different rate. The authors should reveal the rd8 genotyping results to rule out the differential phenotypes that are contributed by the presence of the rd8 allele.

OUR REPLY: We thank again the reviewer for raising this important issue and apologize for not addressing it in detail in the previous version of the manuscript. The P347S A1 line used in this manuscript is in the 129 S2/SvHsd (Harlan, Huntington, UK) background (Chadderton et al., 2009; Loscher et al., 2007; Palfi et al., 2016) and was maintained by crossing these mice among themselves. We added this information in the Materials and Methods section (lines: 425-427). The 129 S2/Sv background is WT for the *rd8* allele, as previously reported (Luhmann et al., 2015; Mattapallil et al., 2012) and as also experimentally confirmed by us (the figure below shows a representative example of the results of a Sanger sequencing analysis on the P347S A1 mouse line of a genomic fragment spanning the *rd8* mutation at the *Crb1* locus, see pink area).

P347S line

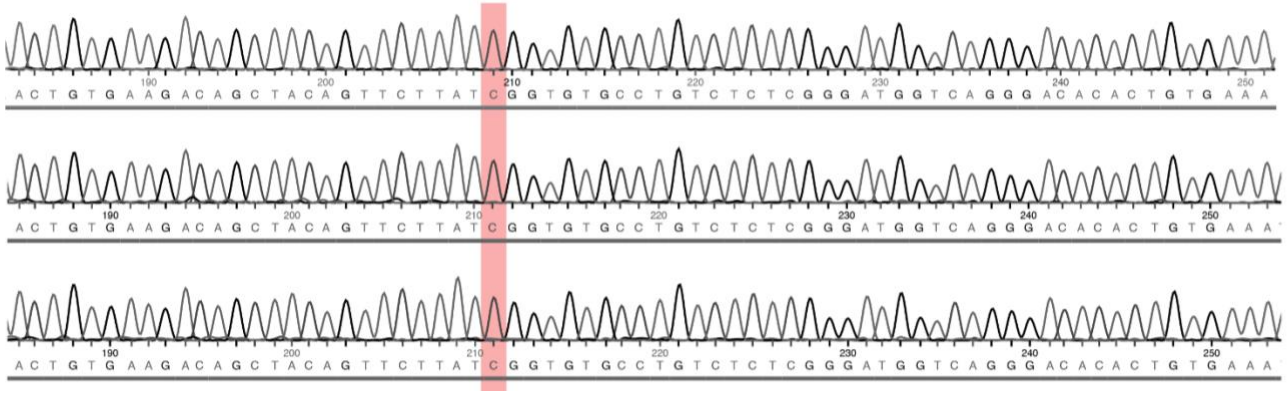

For the experiments reported in the paper, the P347S A1 line was bred with animals in the C57BL/6 background. The cross of the above line with C57BL/6 background mice, possibly harboring a homozygous *rd8* allele, could yield an offspring heterozygous for the *rd8* genotype. It has to be considered that the *rd8* allele is a recessive mutation, and only homozygous mice display a retinal phenotype whereas heterozygous animals are completely normal (Low et al., 2014; Luhmann et al., 2015; Mattapallil et al., 2012).

Nevertheless, we underline that in this work we only used C57BL/6J mice that the Charles Rivers vendor delivered to us thanks to an agreement with the Jackson Labs (JAX® Mice Strain, Charles Rivers Laboratories, Strain Code 632). The latter animals, as correctly pointed out by the Reviewer, are not contaminated with the *rd8* allele. We have now added this information in the Materials and Methods section (lines: 423, 424). As additional precaution, we also screened the acquired C57BL/6J mice for the possible presence of the *rd8* allele. The figure below shows the result of a representative Sanger sequencing analysis of the *Crb1* locus encompassing the *rd8* mutation carried out in the purchased C57BL/6J mice. The analysis reveals no evidence for the presence of the *rd8* allele. These animals have been used to maintain our lines in our animal facility.

C57BL/6J line

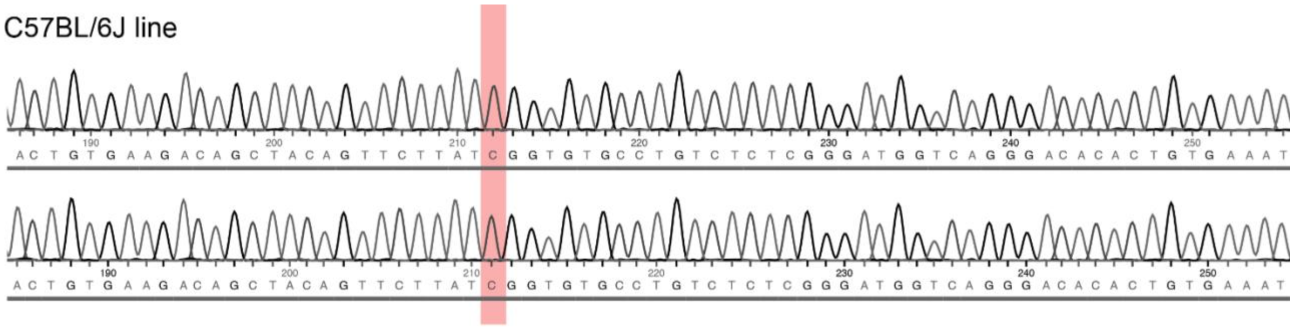

Finally, we point out that, in the case of Sponge-miR-181a/b experiments, animals are injected in one eye with AAV2/8.GFP.Sponge-miR-181a/b and with AAV2/8. GFP (control) in the contralateral eye (as reported in lines 229-230; 245-246 and line 302). In this experimental setting the contribution of the genetic background is therefore minimized.
Based on all of the above considerations, we exclude that the *rd8* allele can contribute to the phenotypic differences that we observe in our experiments.

(2) Endpoints.

2.1. The original paper of P347S mice emphasized the rhodopsin immunostaining was indistinguishable from the WT controls (Figs. 2 and 3 in PMID: 8943080). So it is puzzling why the authors show the rhodopsin mislocalization in the P347S mice and used it as an endpoint (Fig. 1I-Jm Fig. 2A-D, Fig. 5)?

OUR REPLY: Indeed, other reports showed Rhodopsin mislocalization in the ONL in the P347S mouse line [see (Chadderton et al., 2009), Figure 6; (Patrizi et al., 2021), Figure 4; (Marrocco et al., 2021), (Figure 3)]. We believe that the lack of reported Rhodopsin mislocalization in the original paper on the P347S line (Li et al., 1996), correctly reported by the Reviewer, can be due to the following reasons: a) the different time point analyzed: in Li et al, the analysis was performed at P20 while we do the analysis at later timepoints (P30 onward); and b) differences in the antibodies used for the analysis. In the latter respect, Li et al used the monoclonal antibody rho 3A6 (Hodges et al., 1988), which recognizes the human but not the murine rhodopsin whereas we used the anti-Rhodopsin antibody Abcam, ab3267 (see line 496) that recognises both murine WT and human Rhodopsin proteins, as also in the case of Chadderton et al, Patrizi et al, and Marrocco et al.
Therefore, based on all the above considerations, we believe that our data are not in contrast with previous reports and that the impact on rhodopsin mislocalization in P347S mice can be used as a reliable endpoint.
For the sake of clarity, in the revised version of the manuscript, we now mention the previous studies that report on rhodopsin mislocalization in the P347S mouse line (lines: 129-131),

2.2. The other readouts used, such as the thickness of the photoreceptor nuclear layer (ONL), the length of the inner segment (by F-actin and the lengths of the outer segment (by TEM) highly subject to the sectioning planes and the survey areas. Both of this and the single data point cannot convincingly demonstrate the restoration of the photoreceptor structures.

OUR REPLY: We are convinced of the reliability of our analysis showing an improvement of photoreceptor structures following miR-181a/b downregulation but we apologize for not having provided sufficient details in the previous version of the manuscript to fully support our conclusions. We have now modified the manuscript to include additional technical details on the analyses carried out. In particular, the count of nuclei in the ONL, the evaluation of the ONL thickness, the analysis of photoreceptor markers by immunofluorescence, Phalloidin and PNA, Citrate Synthase, Stat3 and active-Casp3 and TUNEL (reported in figure 1, figure 2, figure 3, figure 6, figure 7, figure EV1, figure EV2, figure EV3, Figure EV4, figure EV5,

appendix figure S4 and appendix figure S5) were performed on at least 3 eyes/genotype and only on sections in which the optic nerve was contained (at least 2 sections per eye). Two images from each section were photographed in comparable regions equidistant from the optic nerve head (as reported in lines 481; 483-487; 506-507; 513-515; 524-525). Such kind of selection ensures the characterization of comparable planes and areas (Barbato et al., 2017; Ciampi et al., 2022; Intartaglia et al., 2022; Karali et al., 2020a). Moreover, the OS length was analysed on at least 10 TEM images per eye, for a total number of OS measured of around 200 for the P347S and 150 for the P347S/miR-181a/b-1$^{+/-}$ animals. Only tissue sections in which the sagittal orientation of the connecting cilium (cc) was visible were chosen, as shown in Figure Appendix S1, ensuring the correct sectioning of the eyes (as we report in lines 538-539).

2.3. The mitochondrial populations in photoreceptors are highly heterogeneous, their shape and sizes are dependent on the distance to the outer segment, the time of the harvest, etc. Thus, it is not reliable to depict the size based on the single TEM images, as used by this communication without rigorous experimental description (Fig. 3B-F). Recent investigators have resorted to 3D scanning EM instead due to the problems (e.g., PMID: 34552195; PMID: 33144507).

OUR REPLY: We are aware of the highly heterogeneous shape and size of mitochondria in photoreceptors. Indeed, to avoid daily changes that occur in photoreceptor mitochondria [reported in zebrafish cone photoreceptors, (Giarmarco et al., 2020)] we carried out TEM on retinal samples derived from animals of the same litter and whose eyes were collected at the same time of the day (as mentioned in lines 431-432). We analyzed mitochondrial number, perimeter and area on several TEM images derived from 2 eyes/genotypes, for a total number of 22 images analyzed/genotype for each time point under evaluation. Only tissue sections in which the sagittal orientation of the connecting cilium (cc) was visible were chosen, as shown in Figure Appendix S1, ensuring the correct sectioning of the eyes (as we report in lines 538-539). The mitochondrial characterization was performed for all mitochondria located within the entire inner segment (IS), from the outer limiting membrane to the outer segment (OS) nascent disks. Our images clearly show the presence of a mitochondrial phenotype in P347S mice at both time points analyzed (the newly added p12 and p30), and that the changes in shape and size that we report are independent from localization within the IS (see Figure Appendix S2, also reported below, for Reviewer's convenience). The same is true also for P347S/miR-181$^{+/-}$ mitochondria that, however, show an amelioration of their morphology. In the revised version of the manuscript, we provide in the appendix file (Figure Appendix S2), representative images showing the entire IS region on which we performed the mitochondrial analysis. This experimental methodology has been already reported in other publications (Anastasia et al., 2021; Costa et al., 2010; Indrieri et al., 2019; Mirra et al., 2021; Naon et al., 2016; Ozaki et al., 2020; Quintana-Cabrera et al., 2018; Tsakiri et al., 2019; Xia et al., 2019). We provide more technical details on the procedure in the Materials and Methods section of the revised manuscript (lines 531-552). Moreover, we have also added, in the revised version of the manuscript, additional evidence for the presence of a mitochondrial phenotype by immunofluorescence analysis of a mitochondrial marker (Citrate Synthase, figure EV2 A-E'), at both p12 and p30 and in the Sponge-miR-181a/b treated retinas (figure EV4 J-M and figure EV5 F-I'').

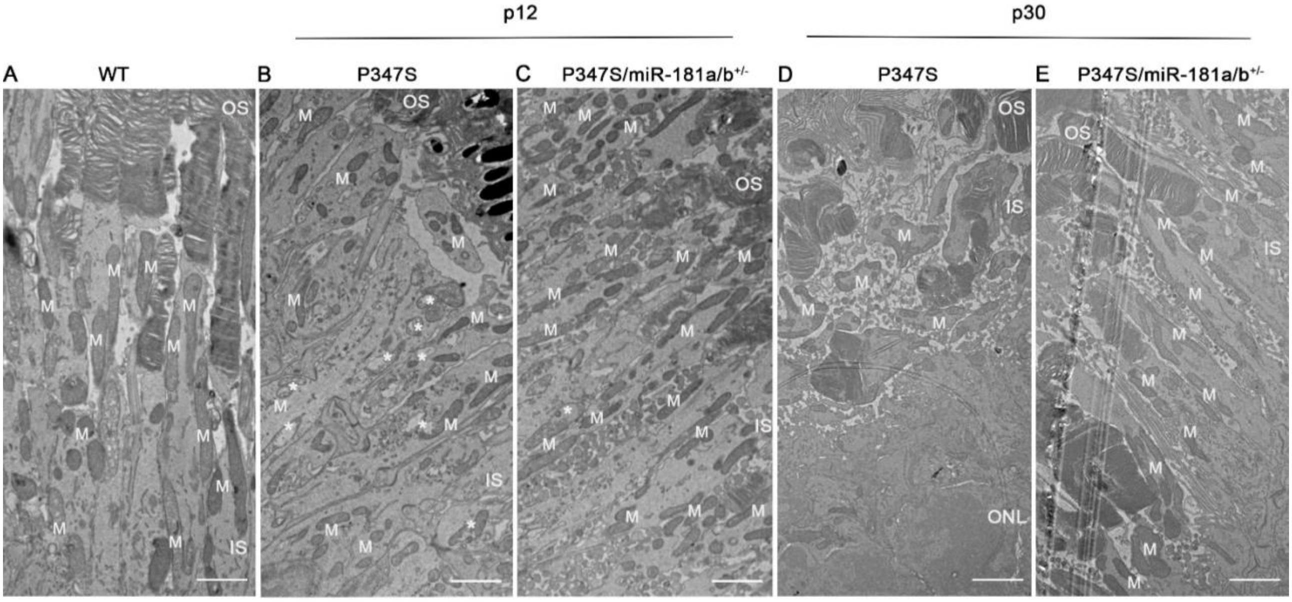

M: mitochondria; OS: Outer segment; IS: Inner Segment; *: swollen mitochondria (scale bars are 2 μm)

The Scale Bars of all figures, including EM, are missing.

OUR REPLY: We strongly apologize for the missing of the error bars. Unfortunately, this omission originates from a technical problem that occurred during the conversion in pdf of the originally uploaded files on the journal website. Indeed, all scale bars are present in the full size .tiff versions uploaded. Nevertheless, we apologize again for not realizing about this problem in the generated pdf file.

2.4. The time point for the mitochondria characterization was when the photoreceptors already look dead (Fig. 3C). Why not be assayed at PN12 to match the transcriptome analysis and to directly address the mitochondria atrophy is an "early" event, i.e., a cause vs. consequence of the cell death.

OUR REPLY: We thank Reviewer 1 for pointing out this important issue. We chose to perform most of our histological phenotypic analysis (including mitochondria characterization) starting at p30 because we wanted to correlate functional amelioration (detected at p30, i.e., the first suitable and reliable time point for ERG analysis) with changes in morphology of retinal structure following miR-181a/b downregulation. However, we believe that the Reviewer's suggestion is extremely well taken and therefore we added TEM analysis and immunofluorescence for a mitochondrial marker at p12, to match the transcriptome analysis, in the revised version of the manuscript (see Lines 169; 176-177; figure 4C, D and figure EV2 A-E'). We believe that the addition of these data strongly improves the characterization of the mitochondrial phenotype in P347S and the quality of our paper.

2.5. Scotopic ERG a-wave represents photoreceptor functions. Several P347S mouse lines still have detectable a-wave (PMID: 8943080). It is curious why the a-wave results were not shown, but only the b-wave data were shown. Since b-wave represents the inner retinal neuron function, the positive results cannot directly conclude the improvement of photoreceptor function. Please show the Scotopic ERG a-wave (taken at different light intensity illumination) of both mutant and WT control mice. The latter control will help to register the extent of the functional restoration relative to the WT mice.

OUR REPLY: The issue raised by the reviewer is very important. Indeed, in the A1 P347S mouse line, the a-wave response is detectable at p30, although dramatically reduced (Li et al., 1996). We would like to point out that, although mostly representing inner retinal function, improvement of b-waves responses represent important readout of photoreceptor amelioration, especially in models with dramatically reduced a-wave responses at early stages of degeneration, as also previously reported (Jiang et al., 2014; Mussolino et al., 2011; Patrizi et al., 2021; Yu et al., 2017). Nevertheless, in the revised version of the manuscript, we now provide the a-wave results (at different light intensity illumination) for all the ERG analyses (figure 2A, figure 3A, figure EV4A and B, figure EV5J and M; appendix figure S3G and appendix figure S5A and M) including also results from WT mice (figure 2A-C). We observed a significant amelioration of a-wave responses in the genetic model and a trend of amelioration in the p4-AAV2/8.CMV.GFP-Sponge-miR-181a/b injected P347S animals at p30. Therefore, also the a-wave results support the functional amelioration conferred by miR-181a/b silencing.

2.6. The authors showed the PNA staining is better preserved in the double transgenic mice (Fig. 1). Since PNA was typically used to highlight the cone outer segments, does the miR-181a/b deficiency improve the cone photopic ERG (a and b waves)?

OUR REPLY: The cone photopic ERG (a- and b-wave results are shown in figures 2A,B and 3A,B of the revised version of the manuscript (b-waves were already reported in the previous manuscript version). We observe a statistically significant amelioration of photopic a-wave and photopic b-wave only at p90, suggesting that the PRs with functional preservation are mostly cones at this late time point.

2.7. To claim for the cell-protective role of miR181a/b, one might have to show the slow-down of the photoreceptor cell death or prevent cell death for early intervention. These are not the case in the current presentation.

OUR REPLY: In the previous version of the manuscript (figure 1B-G) we showed representative images and quantification of TUNEL assays and Active-Casp3 immunofluorescence staining, which supported a significant decrease of cell death extent in the Outer nuclear layer (ONL) following miR-181a/b downregulation. In the revised version of the manuscript, we have added TUNEL assay analysis of AAV2/8.CMV.GFP-Sponge-miR-181a/b-injected eyes vs those injected with AAV2/8.CMV.GFP (Figure EV 4D-H and lines 260-263), confirming the decrease in photoreceptor cell death after miR-181a/b downregulation.

Fig. 2E shows the ONL thickness was increased from 5 to 6 uM. The minor, albeit "statistically significant", the increase was unlikely to be physiologically relevant, considering the WT ONL is up to 50-um thick.

OUR REPLY: Figure 2 (Figure 3 in the revised manuscript) shows data obtained at p90, an advanced time point of retinal degeneration in the P347S animal model, characterized by the presence of only a single row of photoreceptor nuclei. Our data indicate that the miR-181a/b downregulation slows-down disease progression. Although the increased thickness is far from that present in WT conditions, nevertheless it underlies an amelioration of retinal function (as assessed by both ERG and OKR at p90) thus supporting its relevance in slowing down degeneration in this model of autosomal dominant RP, for which at present no treatments are available in patients. Moreover, the OKR results at p90 in P347S mice and the visual tests in Sponge-miR-181a/b treated *rd10* mice highlight that even a small amelioration of ONL thickness and of ERG responses could have a significant impact on visual acuity and visually guided behavioral response (see figure 3N for P347S, and figure 7J, K for *rd10* mice). This apparently small effect could translate into a possible amelioration of quality of life in patients. Indeed, visual acuity and behavioral tests are considered standard assays for evaluation efficacy in clinical trials for ocular disease (Cideciyan et al., 2021; Ferris et al., 1982; Jacobson et al., 2021, 2017; Ma et al., 2022).

The ONL thickness is region-dependent (~50um for central, and ~25um for periphery). Unfortunately, the regional variation was not considered in any of the ONL thickness quantification (Fig. 2E, Fig. EV 1C).

OUR REPLY: To the best of our knowledge**,** there is no literature-based evidence for the presence in the P347S mouse model of a regional pattern in retinal degeneration, which seems to be homogeneous from center to periphery. For this reason, we decided to analyze the ONL thickness on at least 3 eyes/genotype and only on sections in which the optic nerve was contained (at least 2 sections per eye). Two images from each section were photographed in comparable regions equidistant from the optic nerve head (as reported in lines 521-525)

Hence, calling miR-181a/b deficiency has a protective role (Lines 253, 262, 772, ...etc.) is an overstatement.

OUR REPLY: To comply with the Reviewer's request we tuned down our conclusions as reported in lines 41; 150; 220; 238; 267; 283; 288; 291; 319; 324; 329)

2.8. Although the cell-type specificity was well demonstrated for the AAV studies, only a small region was shown. Please show the low-magnification view of the entire retina globe for the overall infection rate.

OUR REPLY: In the revised version of the manuscript, we provide an example of retinas injected with AAV2/8.CMV.GFP-Sponge-miR-181a/b and with AAV2/8.CMV.GFP (control) in the contralateral eye, analyzed by fundoscopy (Appendix FigureS3A, B) and TILE-SCAN images of entire retinal sections (Appendix FigureS3 C-D'). Only sections with comparable GFP expression levels were compared.

Were the eyes with injection-related trauma been excluded for consideration? This information is useful for the consideration of the ERG results, which should be carried out using the criteria as described above.

OUR REPLY We indeed excluded from our analysis eyes that turned out not to be properly injected as assessed from GFP expression analysis, or samples that showed sign of injection-related damages. We added this information in the materials and methods section of the revised version of the manuscript (Lines 644-645).

2.9. The authors used either "eyes" or "tissues" in describing the procedures for RNA isolation (qPCR, transcriptome) and protein isolation (Lines 396, 399, 417, 790). What tissues? Were the whole eyes (containing many cell types including the lens, cornea, etc.) were used?

OUR REPLY: We are very grateful to the Reviewer 1 for highlighting this inaccuracy for which we apologize. We extracted RNA and/or proteins from optic cups, i.e., the eyes after the removal of cornea and lens. In the revised version of the manuscript, we have added this information in the materials and methods section (lines 437; 458; 986).

The changes in the expression of the miR181a/b targets or mitochondria functions of the mixed cell population do not necessarily suggest that these genes are changed in the photoreceptors. Can the authors demonstrate any of the proteins were changed in the photoreceptor cell layers by immunostaining?

OUR REPLY: in the revised version of the manuscript, we added immunofluorescence analysis of Stat3, a direct target of miR-181a/b, observing that the increased levels can be observed in all the retinal cell layers, including the photoreceptor layer (see lines 203-204 and Figure EV3C-G). We have also added immunofluorescence analysis for a mitochondrial marker (Citrate Synthase), at p12 and p30 (Figure EV2 A-E'), that shows the decrease of staining in the IS of photoreceptors (where mitochondria are located) in P347S animals, and an increase in miR-181a/b-downregulated animals, in both the P37S (Figure EV2 A-E' and Figure EV4 J-M) and the *rd10* (Figure EV5 F-I'') models. See lines 176-178; 263-267; 306-308.

3. Others

3.1. The sources of the mouse lines should be acknowledged.

OUR REPLY: We now provide this information in the materials and methods section of the revised version of the manuscript (lines 423; 425-427; 429; 432-434)

3.2. Methods should be written at a detailed level that others can reproduce.

OUR REPLY: We modified the material and methods section of the revised version of the manuscript, by considerably adding previously missing information and by considerably expanding all procedure descriptions (lines: 423-432; 437; 458-459; 483-487; 492-495; 497-498; 504-510; 524-525; 531-538; 541-552; 570-578; 630-638; 644-648)

3.3. The vectors, the helpers, the cell systems, and the purification of AAV should be detailed.

OUR REPLY: We added all the required information in the material and methods section of the revised version of the manuscript (Lines 630-638)

3.4. Erroneous statement or overstatement.

Since only correlative data was provided, LINE 109. "We also observed amelioration of mitochondria morphology in PRs, which is mediated by regulation of the mitochondria fission protein Dra1, mainly via the activation of the Jak2/Stat3 pathway" is an overstatement.

OUR REPLY: We have modified this statement as follows (lines 114-116): "We also observed amelioration of mitochondria morphology in PRs, along with a concomitant downregulation of the mitochondrial fission protein Drp1 mediated by the activation of the JAK2/STAT3 pathway."

Line 60. IRD represents the most important cause of vision impairment in the Western population." is an erroneous statement.

OUR REPLY: We have modified this statement as follows (lines 62-64): "Inherited retinal diseases (IRDs) represent the most important cause of vision impairment <u>of genetic origin</u> in the Western <u>working-age</u> population and include a variety of different clinical subtypes such as retinitis pigmentosa (RP), Leber congenital amaurosis (LCA), and macular diseases."

LINE 101-103 is an erroneous statement. There is no experimental data that supports that mitochondria dysfunction is an "early event" of photoreceptor cell death in P347S rhodopsin mutation. The transcriptome/qPCR/proteins data were obtained from multiple eye cell types. The mitochondria characterization of photoreceptors was carried out at the time point when the photoreceptors were severely atrophic (see above).

OUR REPLY: In this statement of the Introduction, we were not referring to the P347S mouse model but to IRDs as a whole. We have now modified the statement and provided some additional supporting references. The text now reads: (lines 107-111): "The latter cells are under constant environmental challenges and are

highly prone to oxidative stress, a major contributor to retinal degenerations and hence IRDs (Lefevere *et al*, 2017; Mirra & Marfany, 2019; Jiang *et al*, 2022). Importantly, since mitochondrial dysfunction seems to be an early event in IRDs, approaches targeting basic mitochondrial functions hold great therapeutic promise (Lefevere *et al*, 2017; Carrella *et al*, 2021)."

Discussion should be grossly truncated and focus on discussing the experimental results. Speculation should be stated so whenever it is complying.

OUR REPLY: We reduced some parts of the Discussion (see lines 355-357; 375-377; 395-397).

Referee #2 (Comments on Novelty/Model System for Author):

All models used are well established models in the study of IRD.

Referee #2 (Remarks for Author):

The paper titled miR-181a/b downregulation: a mutation-independent therapeutic approach for Inherited Retinal Diseases (IRD) by Sabrina Carrella builds on previous publications demonstrating the toxicity of mir-181 in response to retinal stress.

This work further demonstrates the use of miRNA as possible therapeutic approaches for reducing the severity and progression of degeneration.

The paper provides a strong overview of the current gene therapy landscape and does well to introduce the importance of the topic and the relevance of the studying mir-181. In particular the role of miR-181 to regulate mitochondrial biogenesis is important to the study of IRD as mitochondrial dysfunction is known to be significantly involved with disease onset and progression.

This is a really strong set of data, and a very well written manuscript. I have no hesitation recommending this for publication, with some minor tweaks and suggestions indicated below.

Minor improvements:

Results
As a general critique suggest including the number of animals and the age of the animals in each figure to clearly identify the variabilities in each data set.

OUR REPLY: We are very thankful to the Reviewer 2 for his/her helpful suggestion. We have now added in all figures number age of analyzed animals:

Figure 1:

1. Only A wave responses are shown in figure 1F. Strongly suggest showing representative images of the ERG traces and also include B wave data as well.

OUR REPLY: We added the requested data in the revised version of the manuscript. In particular, both a-waves and b-waves ERG responses and representative curves are now reported in figure 2A-C, figure 3A-C, figure 6 A-C; figure EV4A-C, figure EV5J-O; appendix figure S3G,H and appendix figure S5A-C and M-O.

2. Optical densitometry quantification of the microscopy images to quantify the differences in intensity and locations of the various retinal stains will strengthen this important data.

OUR REPLY: We added the densitometry quantification of immunofluorescence staining for all the analyzed markers (Figure 2P, Figure 3L, Figure 6T and U, Figure 7I, Figure EV1H, Figure EV3G, Figure EV5E, Appendix Figure S4I, Appendix Figure S5L and X).

Figure 2:

1. For consistency in the P90 aged animals please repeat all the same immunohistochemistry straining as per figure 1. This would strengthen the manuscript by showing and comparing the differences between P30 and P90 days.

2. Once again please show the quantification of microscopy images and also the ERG B wave data and representative EGRs as well.

OUR REPLY: We added the above requested data in the revised version of the manuscript. In particular, immunofluorescence panel in figure 3 D-K''; both a-waves and b-waves ERG responses and representative curves in figure 3 A-C. We also added the densitometry quantification of immunofluorescence staining for all the analyzed markers in Figure 3L.

Figure 3:

1. Further context to the RNAseq data is required. What analysis was performed and while it is mentioned this is a reanalysis, further detail in the figure should be provided. Perhaps a table of associated genes used for the analysis would be beneficial, or a list of genes associated with the specific pathways identified.

OUR REPLY: We thank Reviewer 2 for allowing to better clarify this issue. We used our previously generated RNAseq data to compare WT vs P347S animal results (while in our previous paper we prevalently focused on the comparison between transcriptomic data from control-injected vs miR-204-injected P347S mice). To better clarify this aspect, we modified the text, as follows (lines 164-167: "Our previously generated RNA-seq transcriptome data (Karali et al., 2020b) revealed a significant downregulation of mitochondria-related gene pathways at p12 in the P347S retina with respect to wild-type (WT), indicating the presence of early mitochondrial dysfunction even before the appearance of PR degeneration (Fig. 4A, Table EV1)"). We also added an excel file as Table EV1, with the list of the differentially expressed genes associated with the mentioned mitochondrial terms.

2. This is true for all figures but especially for figure 3 E and F. It is difficult to see which is the P values associated with each comparison. Suggest either use lines or * to indicate the significance as well as which are the comparisons being explored.

OUR REPLY: We modified all figures according to the reviewer's suggestion by using lines and different color codes to better highlight the comparisons reported in the panels.

3. While not significant I would suggest including the data of the mitochondrial number in this section. It is good data to show that there is no change in number, but a change in their morphology. This data's inclusion would help make this point more clearly.
OUR REPLY: In the revised version of the manuscript, we moved the graph showing the data on mitochondrial number analysis from a supplementary item to the main figure 4 (panel 4H).

Figure 4:

1. The WB data is not particularly strong. Especially the GAPDH staining which has not provided a great reference level. Particularly for the P347S values which appear significantly stronger than other parts of the gel. Authors should comment on this or redo this analysis.

OUR REPLY: We believe that the GAPDH protein is a good reference in our WB experiments (see for example figure EV2H and figure EV3I). In the specific case of the blot shown in figure 5 (revised version of the manuscript), the difference is due to the amount of proteins loaded on the gel, as also revealed by the PONCEAU RED staining (see bottom-left panel in the figure below, for reviewer only). The upregulation of Drp1 protein levels in P347S with respect to WT samples, is confirmed both when normalizing with GAPDH levels and when normalizing with whole protein content (PONCEAU RED, see right panel in the figure below).

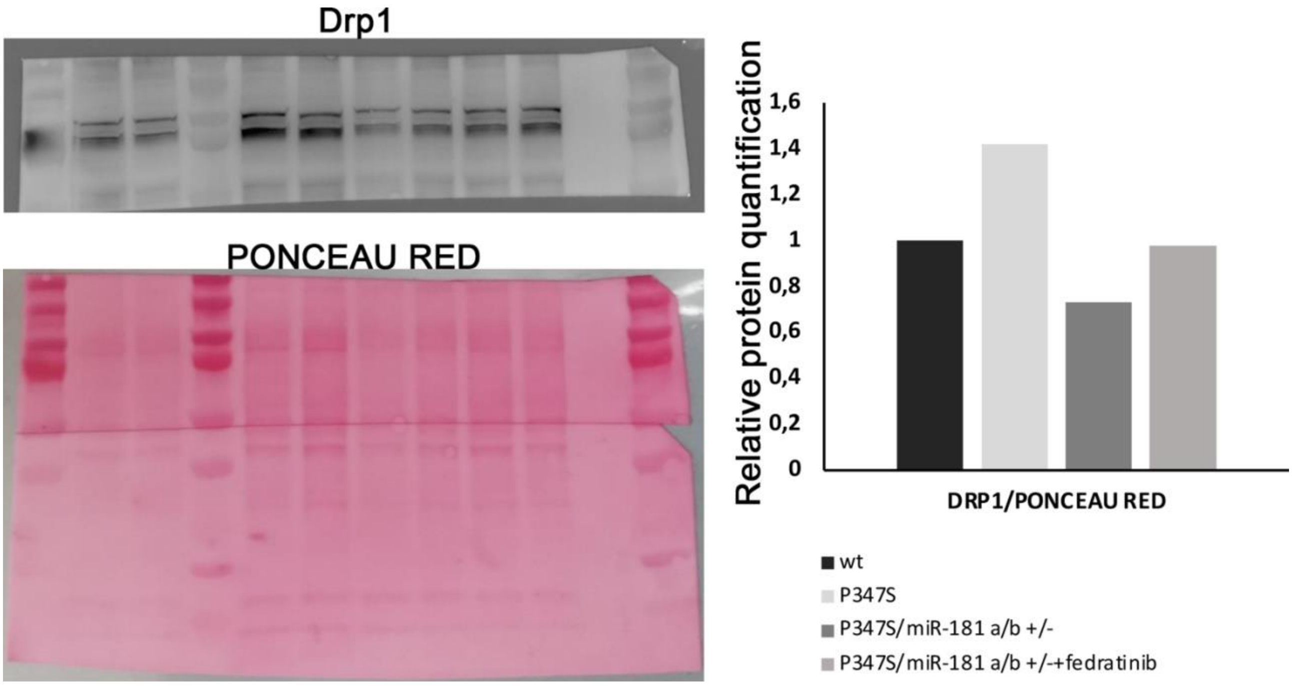

2. All WB data should be shown. At present only representative images for Drp1 are provided. Please provide this data for all other WB performed.

OUR REPLY: All the original data are reported in the appendix file for all the WB data shown in the manuscript (see in the Appendix file the Source Data figure 5A; Source Data figure EV2F; Source Data figure EV2H; Source Data figure EV3A; Source Data figure EV3I).

Figure 5:

1. Again further ERG data is required and the representative images. Also there is no control ERG shown as a baseline reading. This should be included.

OUR REPLY: We added representative ERG curves in Figure 6C (p30) and Figure EV4C (p70) as well as in all other figures displaying ERG data. The dotted red line in the graphs represent the baseline of the respective controls. We better clarify this aspect also in the revised figure legend.

2. The GFP labelling is not shown anywhere in the main images, so it is unclear if the AAV transfection was successful (Note some are placed in the supplementary). Quantification and qualitative data is required to validate these findings and this should all be included in the main results not in the supplementary section.

OUR REPLY: We now provide for all the immunofluorescence images in the paper the GFP channel to demonstrate that the staining reported indeed derived from injected areas. We also provide densitometry quantification for all panels (Figure 6T and U, Figure 7I, Figure EV1H, Figure EV3G, Figure EV5E, Appendix Figure S4I, Appendix Figure S5L and X).

Figure 6:

1. Justification of the different functional tests are required. Did the ERGs not show a positive result? Suggest consistency of tests across the experiments reported in this manuscript.

OUR REPLY: We indeed carried out ERG analysis on *rd10* injected animals at p30 and we now include these data in the revised manuscript (figure EV5 J-O). Although there is a trend towards improvement after AAV2/8-CMV-GFP.miR-181a/b sponge treatment, we could observe only a significant amelioration of b-wave responses in p4-injected AAV2/8.CMV.GFP-Sponge-miR-181a/b eyes, probably due to the advanced retinal degeneration progression (e.g. massively impaired ERG response and dramatically reduced ONL thickness) observed at p30 in this model (Chang et al., 2002). Therefore, we decided to use additional visual functional assays that allow to highlight even slight improvements in visual performance, such as pupillary light responses (PLR) and Water Maze tests (as mentioned in lines 308-313).

Discussion
Really strong discussion with no suggestions for improvement. This was a pleasure to read.

OUR REPLY: We thank the Reviewer for this comment.

Referee #3 (Comments on Novelty/Model System for Author):

Focusing on mitochondria is not new to rescue inherited retinal diseases but focusing on miR-181a/b is novel approach and impact is high if true. They have already published one paper in EMBO Mol Med for the basics of this micro RNA to rescue and maintain mitochondria structure.

Referee #3 (Remarks for Author):

Submitted manuscript by Carrella et al., focus on miR-181a/b, which targets number of gene transcript of cox11, erk2, mcl1, bcl2, nrf1, coq10b, park2 and others leading to the biogenesis, clearance and detoxification ROS in mitochondria. Authors demonstrate induction of drp1 protein by miR-181a/b suppression mainly via activation of Jak2/Stat3 pathway by QT-PCR. Author have shown previously that inactivation of miR-181a/b protects RGC in LHON mice model. In this manuscript authors have used the same method on P347S rhodopsin dominant and recessive rd10 retinitis pigmentosa mouse models. AAV2/8 vector was used to deliver RHO-Sponge-miR-181a/b into the subretinal space for P347S Tg-mice and rd10 mice. miR-181a/b-1 +/+ mice was mated with P347S Tg-mice to generate genetically suppressed mice of miR-181a/b. The suppression was performed by Suppression of miR-181a/b do not cause abnormality in normal mice.

Comments to authors,

Authors has shown results of rescuing both dominant and recessive RP by modulating mitochondria through miR-181a/b suppression. The aim is promising but additional information is needed for positive conclusion.

1.Please add scale for all photos of tissue sections in this manuscript.

OUR REPLY: We are very thankful to the reviewers for highlighting the lack of Scale Bars. As already mentioned in the response to the other reviewers, this omission originates from a technical problem that occurred during the conversion in pdf of the originally uploaded files on the journal website. Indeed, all scale bars are present in the full size .tiff versions uploaded. Nevertheless, we apologize again for not realizing about this problem in the generated pdf file.

2.Fig.1: Please show photo of full retina to observe full structure of the photoreceptor and other area of retina.

OUR REPLY: In the previous version of the manuscript, a full retina section was shown in Figure EV1 A and B. We could not observe any apparent alteration in the other retinal layers in the P347S mouse model. However, in the revised version of the manuscript we lowered the magnification of the immunofluorescence staining reported in Figure 2 and 3, to allow a better and broader visualization of the photoreceptor layer and of other retinal areas.

3.Fig.3B: Please add scale and organelle name in the photo.

OUR REPLY: As for point 1, we apologize for the technical problem that led to the lack of scale bars. In the revised version of the manuscript, we now provide also organelle name in this panel.

4.Fig.5 & Fig.6: Additional immunostaining of endogenous photoreceptor makers is needed to assess the condition of the retina. Normal mouse section should be added for this assessment and the entire retina should be shown.

OUR REPLY: In the revised version of the manuscript, we added additional immunostaining of for the Recoverin photoreceptor marker, both in the case of the genetic miR-181a/b silencing (Figure EV1 E-G') and of AAV-injections, both in the P347S (Appendix Figure S4A-I) and rd10 mouse models (Figure EV5A-D'). Immunostainings on WT retinal sections for all the photoreceptor marker used are shown in figure 2 (D,D'; G,G'; J, J'; M,M',M'') and in Figure EV1 E,E'.

Mitochondria staining should be also added to see how much is remaining compare to normal retina and non-treated disease mice.

OUR REPLY: In the revised version of the manuscript, we provide staining data for a mitochondrial marker, i.e., Citrate Synthase (CS), both in the case of the genetic model (Figure EV2A-E') and of AAV -based injections in both the P347S (Figure EV4J-M) and *rd10* mouse models (Figure EV5F-I''). CS immunostaining on WT retina and non-treated IRD mice is shown in Figure EV2 panel A, B and D.

Bibliography:

Anastasia, I., Ilacqua, N., Raimondi, A., Lemieux, P., Ghandehari-Alavijeh, R., Faure, G., Mekhedov, S.L., Williams, K.J., Caicci, F., Valle, G., Giacomello, M., Quiroga, A.D., Lehner, R., Miksis, M.J., Toth, K., de Aguiar Vallim, T.Q., Koonin, E.V., Scorrano, L., Pellegrini, L., 2021. Mitochondria-rough-ER contacts in the liver regulate systemic lipid homeostasis. Cell Reports 34, 108873. https://doi.org/10.1016/j.celrep.2021.108873

Barbato, S., Marrocco, E., Intartaglia, D., Pizzo, M., Asteriti, S., Naso, F., Falanga, D., Bhat, R.S., Meola, N., Carissimo, A., Karali, M., Prosser, H.M., Cangiano, L., Surace, E.M., Banfi, S., Conte, I., 2017. MiR-211 is essential for adult cone photoreceptor maintenance and visual function. Sci Rep 7, 17004. https://doi.org/10.1038/s41598-017-17331-z

Carrella, S., Massa, F., Indrieri, A., 2021. The Role of MicroRNAs in Mitochondria-Mediated Eye Diseases. Front. Cell Dev. Biol. 9, 653522. https://doi.org/10.3389/fcell.2021.653522

Chadderton, N., Millington-Ward, S., Palfi, A., O'Reilly, M., Tuohy, G., Humphries, M.M., Li, T., Humphries, P., Kenna, P.F., Farrar, G.J., 2009. Improved Retinal Function in a Mouse Model of Dominant Retinitis Pigmentosa Following AAV-delivered Gene Therapy. Molecular Therapy 17, 593–599. https://doi.org/10.1038/mt.2008.301

Chang, B., Hawes, N.L., Hurd, R.E., Davisson, M.T., Nusinowitz, S., Heckenlively, J.R., 2002. Retinal degeneration mutants in the mouse. Vision Research 42, 517–525. https://doi.org/10.1016/S0042-6989(01)00146-8

Ciampi, L., Mantica, F., López-Blanch, L., Permanyer, J., Rodriguez-Marín, C., Zang, J., Cianferoni, D., Jiménez-Delgado, S., Bonnal, S., Miravet-Verde, S., Ruprecht, V., Neuhauss, S.C.F., Banfi, S., Carrella, S., Serrano, L., Head, S.A., Irimia, M., 2022. Specialization of the photoreceptor transcriptome by *Srrm3* -dependent microexons is required for outer segment maintenance and vision. Proc. Natl. Acad. Sci. U.S.A. 119, e2117090119. https://doi.org/10.1073/pnas.2117090119

Cideciyan, A.V., Krishnan, A.K., Roman, A.J., Sumaroka, A., Swider, M., Jacobson, S.G., 2021. Measures of Function and Structure to Determine Phenotypic Features, Natural History, and Treatment Outcomes in Inherited Retinal Diseases. Annu. Rev. Vis. Sci. 7, 747–772. https://doi.org/10.1146/annurev-vision-032321-091738

Costa, V., Giacomello, M., Hudec, R., Lopreiato, R., Ermak, G., Lim, D., Malorni, W., Davies, K.J.A., Carafoli, E., Scorrano, L., 2010. Mitochondrial fission and cristae disruption increase the response of cell models of Huntington's disease to apoptotic stimuli. EMBO Mol Med 2, 490–503. https://doi.org/10.1002/emmm.201000102

Ferris, F.L., Kassoff, A., Bresnick, G.H., Bailey, I., 1982. New visual acuity charts for clinical research. Am J Ophthalmol 94, 91–96.

Giarmarco, M.M., Brock, D.C., Robbings, B.M., Cleghorn, W.M., Tsantilas, K.A., Kuch, K.C., Ge, W., Rutter, K.M., Parker, E.D., Hurley, J.B., Brockerhoff, S.E., 2020. Daily mitochondrial dynamics in cone photoreceptors. Proc Natl Acad Sci U S A 117, 28816–28827. https://doi.org/10.1073/pnas.2007827117

Hodges, R.S., Heaton, R.J., Parker, J.M., Molday, L., Molday, R.S., 1988. Antigen-antibody interaction. Synthetic peptides define linear antigenic determinants recognized by monoclonal antibodies directed to the cytoplasmic carboxyl terminus of rhodopsin. J Biol Chem 263, 11768–11775.

Indrieri, A., Carrella, S., Romano, A., Spaziano, A., Marrocco, E., Fernandez-Vizarra, E., Barbato, S., Pizzo, M., Ezhova, Y., Golia, F.M., Ciampi, L., Tammaro, R., Henao-Mejia, J., Williams, A., Flavell, R.A., De Leonibus, E., Zeviani, M., Surace, E.M., Banfi, S., Franco, B., 2019. miR-181a/b downregulation exerts a protective action on mitochondrial disease models. EMBO Mol Med 11, e8734. https://doi.org/10.15252/emmm.201708734

Intartaglia, D., Giamundo, G., Naso, F., Nusco, E., Di Giulio, S., Salierno, F.G., Polishchuk, E., Conte, I., 2022. Induction of Autophagy Promotes Clearance of RHOP23H Aggregates and Protects From Retinal Degeneration. Front. Aging Neurosci. 14, 878958. https://doi.org/10.3389/fnagi.2022.878958

Jacobson, S.G., Cideciyan, A.V., Ho, A.C., Peshenko, I.V., Garafalo, A.V., Roman, A.J., Sumaroka, A., Wu, V., Krishnan, A.K., Sheplock, R., Boye, S.L., Dizhoor, A.M., Boye, S.E., 2021. Safety and improved efficacy signals following gene therapy in childhood blindness caused by GUCY2D mutations. iScience 24, 102409. https://doi.org/10.1016/j.isci.2021.102409

Jacobson, S.G., Cideciyan, A.V., Sumaroka, A., Roman, A.J., Charng, J., Lu, M., Choudhury, S., Schwartz, S.B., Heon, E., Fishman, G.A., Boye, S.E., 2017. Defining Outcomes for Clinical Trials of Leber Congenital Amaurosis Caused by GUCY2D Mutations. American Journal of Ophthalmology 177, 44–57. https://doi.org/10.1016/j.ajo.2017.02.003

Jiang, K., Mondal, A.K., Adlakha, Y.K., Gumerson, J., Aponte, A., Gieser, L., Kim, J.-W., Boleda, A., Brooks, M.J., Nellissery, J., Fox, D.A., Balaban, R., Covian, R., Swaroop, A., 2022. Multiomics analyses reveal early metabolic imbalance and mitochondrial stress in neonatal photoreceptors leading to cell death in Pde6brd1/rd1 mouse model of retinal degeneration. Hum Mol Genet 31, 2137–2154. https://doi.org/10.1093/hmg/ddac013

Jiang, K., Wright, K.L., Zhu, P., Szego, M.J., Bramall, A.N., Hauswirth, W.W., Li, Q., Egan, S.E., McInnes, R.R., 2014. STAT3 promotes survival of mutant photoreceptors in inherited photoreceptor degeneration models. Proc. Natl. Acad. Sci. U.S.A. 111. https://doi.org/10.1073/pnas.1411248112

Karali, M., Guadagnino, I., Marrocco, E., De Cegli, R., Carissimo, A., Pizzo, M., Casarosa, S., Conte, I., Surace, E.M., Banfi, S., 2020a. AAV-miR-204 Protects from Retinal Degeneration by Attenuation of Microglia Activation and Photoreceptor Cell Death. Molecular Therapy - Nucleic Acids 19, 144–156. https://doi.org/10.1016/j.omtn.2019.11.005

Lefevere, E., Toft-Kehler, A.K., Vohra, R., Kolko, M., Moons, L., Van Hove, I., 2017. Mitochondrial dysfunction underlying outer retinal diseases. Mitochondrion 36, 66–76. https://doi.org/10.1016/j.mito.2017.03.006

Li, T., Snyder, W.K., Olsson, J.E., Dryja, T.P., 1996. Transgenic mice carrying the dominant rhodopsin mutation P347S: evidence for defective vectorial transport of rhodopsin to the outer segments. Proc Natl Acad Sci U S A 93, 14176–14181. https://doi.org/10.1073/pnas.93.24.14176

Loscher, C.J., Hokamp, K., Kenna, P.F., Ivens, A.C., Humphries, P., Palfi, A., Farrar, G.J., 2007. Altered retinal microRNA expression profile in a mouse model of retinitis pigmentosa. Genome Biol 8, R248. https://doi.org/10.1186/gb-2007-8-11-r248

Low, B.E., Krebs, M.P., Joung, J.K., Tsai, S.Q., Nishina, P.M., Wiles, M.V., 2014. Correction of the *Crb1*^{rd8} Allele and Retinal Phenotype in C57BL/6N Mice Via TALEN-Mediated Homology-Directed Repair. Invest. Ophthalmol. Vis. Sci. 55, 387. https://doi.org/10.1167/iovs.13-13278

Luhmann, U.F.O., Carvalho, L.S., Holthaus, S.-M. kleine, Cowing, J.A., Greenaway, S., Chu, C.J., Herrmann, P., Smith, A.J., Munro, P.M.G., Potter, P., Bainbridge, J.W.B., Ali, R.R., 2015. The severity of retinal pathology in homozygous Crb1rd8/rd8 mice is dependent on additional genetic factors. Human Molecular Genetics 24, 128–141. https://doi.org/10.1093/hmg/ddu424

Ma, X., Sechrest, E.R., Fajardo, D., Zhu, P., Dyka, F., Wang, Y., Lobanova, E., Boye, S.E., Baehr, W., Deng, W.-T., 2022. Gene Therapy in *Opn1mw*$^{-/-}$/*Opn1sw*$^{-/-}$ Mice and Implications for Blue Cone Monochromacy Patients with Deletion Mutations. Human Gene Therapy 33, 708–718. https://doi.org/10.1089/hum.2021.298

Marrocco, E., Maritato, R., Botta, S., Esposito, M., Surace, E.M., 2021. Challenging Safety and Efficacy of Retinal Gene Therapies by Retinogenesis. IJMS 22, 5767. https://doi.org/10.3390/ijms22115767

Mattapallil, M.J., Wawrousek, E.F., Chan, C.-C., Zhao, H., Roychoudhury, J., Ferguson, T.A., Caspi, R.R., 2012. The Rd8 mutation of the Crb1 gene is present in vendor lines of C57BL/6N mice and embryonic stem cells, and confounds ocular induced mutant phenotypes. Invest Ophthalmol Vis Sci 53, 2921–2927. https://doi.org/10.1167/iovs.12-9662

Mirra, S., García-Arroyo, R., B. Domènech, E., Gavaldà-Navarro, A., Herrera-Úbeda, C., Oliva, C., Garcia-Fernàndez, J., Artuch, R., Villarroya, F., Marfany, G., 2021. CERKL, a retinal dystrophy gene, regulates mitochondrial function and dynamics in the mammalian retina. Neurobiology of Disease 156, 105405. https://doi.org/10.1016/j.nbd.2021.105405

Mirra, S., Marfany, G., 2019. Mitochondrial Gymnastics in Retinal Cells: A Resilience Mechanism Against Oxidative Stress and Neurodegeneration, in: Bowes Rickman, C., Grimm, C., Anderson, R.E., Ash, J.D., LaVail, M.M., Hollyfield, J.G. (Eds.), Retinal Degenerative Diseases, Advances in Experimental Medicine and Biology. Springer International Publishing, Cham, pp. 513–517. https://doi.org/10.1007/978-3-030-27378-1_84

Mussolino, C., Sanges, D., Marrocco, E., Bonetti, C., Di Vicino, U., Marigo, V., Auricchio, A., Meroni, G., Surace, E.M., 2011. Zinc-finger-based transcriptional repression of rhodopsin in a model of dominant retinitis pigmentosa. EMBO Mol Med 3, 118–128. https://doi.org/10.1002/emmm.201000119

Naon, D., Zaninello, M., Giacomello, M., Varanita, T., Grespi, F., Lakshminaranayan, S., Serafini, A., Semenzato, M., Herkenne, S., Hernández-Alvarez, M.I., Zorzano, A., De Stefani, D., Dorn, G.W., Scorrano, L., 2016. Critical reappraisal confirms that Mitofusin 2 is an endoplasmic reticulum–mitochondria tether. Proc. Natl. Acad. Sci. U.S.A. 113, 11249–11254. https://doi.org/10.1073/pnas.1606786113

Ozaki, T., Utsumi, S., Iwamoto, T., Tanaka, M., Tomita, H., Sugano, E., Ishiyama, E., Ishida, K., 2020. Data on mitochondrial ultrastructure of photoreceptors in pig, rabbit, and mouse retinas. Data in Brief 30, 105544. https://doi.org/10.1016/j.dib.2020.105544

Palfi, A., Hokamp, K., Hauck, S.M., Vencken, S., Millington-Ward, S., Chadderton, N., Carrigan, M., Kortvely, E., Greene, C.M., Kenna, P.F., Farrar, G.J., 2016. microRNA regulatory circuits in a mouse model of inherited retinal degeneration. Sci Rep 6, 31431. https://doi.org/10.1038/srep31431

Patrizi, C., Llado, M., Benati, D., Iodice, C., Marrocco, E., Guarascio, R., Surace, E.M., Cheetham, M.E., Auricchio, A., Recchia, A., 2021. Allele-specific editing ameliorates dominant retinitis pigmentosa in a transgenic mouse model. The American Journal of Human Genetics 108, 295–308. https://doi.org/10.1016/j.ajhg.2021.01.006

Quintana-Cabrera, R., Quirin, C., Glytsou, C., Corrado, M., Urbani, A., Pellattiero, A., Calvo, E., Vázquez, J., Enríquez, J.A., Gerle, C., Soriano, M.E., Bernardi, P., Scorrano, L., 2018. The cristae modulator Optic atrophy 1 requires mitochondrial ATP synthase oligomers to safeguard mitochondrial function. Nat Commun 9, 3399. https://doi.org/10.1038/s41467-018-05655-x

Tsakiri, E.N., Gumeni, S., Vougas, K., Pendin, D., Papassideri, I., Daga, A., Gorgoulis, V., Juhász, G., Scorrano, L., Trougakos, I.P., 2019. Proteasome dysfunction induces excessive proteome instability and loss of mitostasis that can be mitigated by enhancing mitochondrial fusion or autophagy. Autophagy 15, 1757–1773. https://doi.org/10.1080/15548627.2019.1596477

Xia, H., Hu, Q., Li, L., Tang, X., Zou, J., Huang, L., Li, X., 2019. Protective effects of autophagy against blue light-induced retinal degeneration in aged mice. Sci. China Life Sci. 62, 244–256. https://doi.org/10.1007/s11427-018-9357-y

Yu, W., Mookherjee, S., Chaitankar, V., Hiriyanna, S., Kim, J.-W., Brooks, M., Ataeijannati, Y., Sun, X., Dong, L., Li, T., Swaroop, A., Wu, Z., 2017. Nrl knockdown by AAV-delivered CRISPR/Cas9 prevents retinal degeneration in mice. Nat Commun 8, 14716. https://doi.org/10.1038/ncomms14716

8th Sep 2022

Dear Dr. Carrella,

Thank you for the submission of your revised manuscript to EMBO Molecular Medicine and please accept my apologies for the delay in getting back to you as the reports came in when I was on annual leave. We have received the enclosed reports from the three referees who re-reviewed your manuscript. As you will see, they are now supportive of publication, and I am therefore pleased to inform you that we will be able to accept your manuscript once the following editorial points will be addressed:

1/ Referees:
- Please address the remaining minor concerns from referee #1.

2/ Main manuscript text:
- Your text was cross-checked for similarities with other manuscripts, and resemblances were found with other texts. Please modify your text accordingly (see the parts of your text highlighted in the attached documents).
- Please address the queries from our data editors in track changes mode in the main manuscript file labelled 'Data edited MS file'. Kindly use this file for any further modification, remove the red text and only keep in track changes mode any new modification.
- Material and methods: please indicate the gender of the mice used in the experiments as well as the housing and husbandry conditions.
- Author contribution: CRediT has replaced the traditional author contributions section because it offers a systematic machine-readable author contributions format that allows for more effective research assessment. Please remove the Authors Contributions from the manuscript and use the free text boxes beneath each contributing author's name in our system to add specific details on the author's contribution. More information is available in our guide to authors.
- Please add a specific section "Disclosure statement and competing interests":
We updated our journal's competing interests policy in January 2022 and request authors to consider both actual and perceived competing interests. Please review the policy https://www.embopress.org/competing-interests and update your competing interests if necessary.

3/ Figures and Appendix:
- Carefully check that all figures and figure panels are referenced in the manuscript text (callouts are currently missing for Fig. 2P, Fig. 6F-K, N, O, R, and S).
- Figure EV2F: Please add a black line to make the intentional splice clearer.
- Figures 4 and EV1E-H: you indicated N{greater than or equal to}2 in the figure legends, please clarify. Bar graphs should be replaced by scatter plots showing individual datapoints.
- Please add a legend to the file Table EV1.
- Please remove the source data from the Appendix file, which should contain the Appendix Figures S1-5 with their legends and Appendix Table S1. Kindly add a table of content.

4/ Thank you for providing Source Data. Please upload them separately, one file per figure, and then zipped in one zip file.

5/ Synopsis: I slightly edited your text to fit our style and format, please let me know if you agree with the following or amend as you see fit:

The application of gene-specific approaches for the treatment of Inherited Retinal Diseases (IRDs) is hampered by their broad genetic heterogeneity. Modulation of the expression of miR-181a/b in the retina represents a promising gene-independent therapeutic strategy for these conditions.
- miR-181a/b downregulation slows down retinal degeneration in an in vivo model for a dominant form of IRD, i.e., the transgenic RHO-P347S mouse.
- miR-181a/b downregulation ameliorates the retinal phenotype of an animal model for a recessive form of IRD, i.e., the rd10 mouse.
- Photoreceptor cells of RHO-P347S mice show an early mitochondrial dysfunction that is counteracted by miR-181a/b downregulation

Thank you for providing a visual abstract. Please resize it as a PNG/JPEG/TIF file 550 px wide x 300-600 px high.

6/ As part of the EMBO Publications transparent editorial process initiative (see our Editorial at http://embomolmed.embopress.org/content/2/9/329), EMBO Molecular Medicine will publish online a Review Process File (RPF) to accompany accepted manuscripts.
This file will be published in conjunction with your paper and will include the anonymous referee reports, your point-by-point

response and all pertinent correspondence relating to the manuscript. Let us know whether you agree with the publication of the RPF and as here, if you want to remove or not any figures from it prior to publication.
Please note that the Authors checklist will be published at the end of the RPF.

I look forward to receiving your revised manuscript.

Yours sincerely,

Lise Roth

Lise Roth, PhD
Senior Editor
EMBO Molecular Medicine

To submit your manuscript, please follow this link:

Link Not Available

The system will prompt you to fill in your funding and payment information. This will allow Wiley to send you a quote for the article processing charge (APC) in case of acceptance. This quote takes into account any reduction or fee waivers that you may be eligible for. Authors do not need to pay any fees before their manuscript is accepted and transferred to our publisher.

***** Reviewer's comments *****

Referee #1 (Remarks for Author):

1. Now more detail on OKT methodology has been provided; it stated " increasing spatial frequency for each direction is assayed" (Line 577). So, please clarify what value was shown in Fig. 3N. The standard presentation for OKT is to show the visual acuity plot fitting across a range of spatial frequencies. This animal behavior assays need many trials to obtain reliable data. Please state how many trials per mouse for each condition were tested in Methods.
2. Pleas state the mice tested do not carry rd8 allele in the Methods.
3. Please state the genes tested in qPCR (Fig. 1A) in the text (Line137).
4. Please spell out the proteins tested in WB on Line 177 (for Fig. EV2F, G).

Referee #2 (Remarks for Author):

The authors have addressed all of my concerns and I recommend no further changes.

Referee #3 (Remarks for Author):

Authors has ansered to all my questions.

The authors performed the requested editorial changes.

19th Sep 2022

Dear Dr. Carrella,

Thank you for submitting your revised files. I am pleased to inform you that your manuscript is accepted for publication and is now being sent to our publisher to be included in the next available issue of EMBO Molecular Medicine.

Please read below for additional IMPORTANT information regarding your article, its publication and the production process.

Congratulations on your interesting work,

With kind regards,

Lise Roth

Lise Roth, Ph.D
Senior Editor
EMBO Molecular Medicine

Follow us on Twitter @EmboMolMed
Sign

EMBO Press Author Checklist

Corresponding Author Name: Sabrina Carrella and Sandro Banfi	
Journal Submitted to: EMBO Molecular Medicine	
Manuscript Number: EMM-2022-15941	

USEFUL LINKS FOR COMPLETING THIS FORM
The EMBO Journal - Author Guidelines
EMBO Reports - Author Guidelines
Molecular Systems Biology - Author Guidelines
EMBO Molecular Medicine - Author Guidelines

Reporting Checklist for Life Science Articles (updated January 2022)
This checklist is adapted from Materials Design Analysis Reporting (MDAR) Checklist for Authors. MDAR establishes a minimum set of requirements in transparent reporting in the life sciences (see Statement of Task: 10.31222/osf.io/9sm4x). Please follow the journal's guidelines in preparing your manuscript.
Please note that a copy of this checklist will be published alongside your article.

Abridged guidelines for figures
1. Data
The data shown in figures should satisfy the following conditions:
- the data were obtained and processed according to the field's best practice and are presented to reflect the results of the experiments in an accurate and unbiased manner.
- ideally, figure panels should include only measurements that are directly comparable to each other and obtained with the same assay.
- plots include clearly labeled error bars for independent experiments and sample sizes. Unless justified, error bars should not be shown for technical replicates.
- if n<5, the individual data points from each experiment should be plotted. Any statistical test employed should be justified.
- Source Data should be included to report the data underlying figures according to the guidelines set out in the authorship guidelines on Data Presentation.

2. Captions
Each figure caption should contain the following information, for each panel where they are relevant:
- a specification of the experimental system investigated (eg cell line, species name).
- the assay(s) and method(s) used to carry out the reported observations and measurements.
- an explicit mention of the biological and chemical entity(ies) that are being measured.
- an explicit mention of the biological and chemical entity(ies) that are altered/varied/perturbed in a controlled manner.
- the exact sample size (n) for each experimental group/condition, given as a number, not a range;
- a description of the sample collection allowing the reader to understand whether the samples represent technical or biological replicates (including how many animals, litters, cultures, etc.).
- a statement of how many times the experiment shown was independently replicated in the laboratory.
- definitions of statistical methods and measures:
  - common tests, such as t-test (please specify whether paired vs. unpaired), simple χ2 tests, Wilcoxon and Mann-Whitney tests, can be unambiguously identified by name only, but more complex techniques should be described in the methods section;
  - are tests one-sided or two-sided?
  - are there adjustments for multiple comparisons?
  - exact statistical test results, e.g., P values = x but not P values < x;
  - definition of 'center values' as median or average;
  - definition of error bars as s.d. or s.e.m.

Please complete ALL of the questions below.
Select "Not Applicable" only when the requested information is not relevant for your study.

Materials

Newly Created Materials	Information included in the manuscript?	In which section is the information available? (Reagents and Tools Table, Materials and Methods, Figures, Data Availability Section)
New materials and reagents need to be available; do any restrictions apply?	Not Applicable	

Antibodies	Information included in the manuscript?	In which section is the information available? (Reagents and Tools Table, Materials and Methods, Figures, Data Availability Section)
For **antibodies** provide the following information:<br>- Commercial antibodies: RRID (if possible) or supplier name, catalogue number and or/clone number<br>- Non-commercial: RRID or citation	Yes	Materials and Methods

DNA and RNA sequences	Information included in the manuscript?	In which section is the information available? (Reagents and Tools Table, Materials and Methods, Figures, Data Availability Section)
**Short novel DNA or RNA including primers, probes**: provide the sequences.	Yes	Appendix Table S1

Cell materials	Information included in the manuscript?	In which section is the information available? (Reagents and Tools Table, Materials and Methods, Figures, Data Availability Section)
**Cell lines:** Provide species information, strain. Provide accession number in repository **OR** supplier name, catalog number, clone number, and/**OR** RRID.	Not Applicable	
**Primary cultures:** Provide species, strain, sex of origin, genetic modification status.	Not Applicable	
Report if the cell lines were recently **authenticated** (e.g., by STR profiling) and tested for mycoplasma contamination.	Not Applicable	

Experimental animals	Information included in the manuscript?	In which section is the information available? (Reagents and Tools Table, Materials and Methods, Figures, Data Availability Section)
**Laboratory animals or Model organisms:** Provide species, strain, sex, age, genetic modification status. Provide accession number in repository **OR** supplier name, catalog number, clone number, **OR** RRID.	Yes	Materials and Methods
**Animal observed in or captured from the field:** Provide species, sex, and age where possible.	Not Applicable	
Please detail **housing and husbandry conditions**.	Yes	Materials and Methods

Plants and microbes	Information included in the manuscript?	In which section is the information available? (Reagents and Tools Table, Materials and Methods, Figures, Data Availability Section)
**Plants:** provide species and strain, ecotype and cultivar where relevant, unique accession number if available, and source (including location for collected wild specimens).	Not Applicable	
**Microbes:** provide species and strain, unique accession number if available, and source.	Not Applicable	

Human research participants	Information included in the manuscript?	In which section is the information available? (Reagents and Tools Table, Materials and Methods, Figures, Data Availability Section)
If collected and within the bounds of privacy constraints report on age, sex and gender or ethnicity for all study participants.	Not Applicable	

Core facilities	Information included in the manuscript?	In which section is the information available? (Reagents and Tools Table, Materials and Methods, Figures, Data Availability Section)
If your work benefited from core facilities, was their service mentioned in the acknowledgments section?	Yes	Materials and Methods and Acknowledgments sections

Design

Study protocol	Information included in the manuscript?	In which section is the information available? (Reagents and Tools Table, Materials and Methods, Figures, Data Availability Section)
If study protocol has been **pre-registered, provide DOI in the manuscript**. For clinical trials, provide the trial registration number **OR** cite DOI.	Not Applicable	
Report the **clinical trial registration number** (at ClinicalTrials.gov or equivalent), where applicable.	Not Applicable	

Laboratory protocol	Information included in the manuscript?	In which section is the information available? (Reagents and Tools Table, Materials and Methods, Figures, Data Availability Section)
Provide DOI OR other citation details if **external detailed step-by-step protocols** are available.	Not Applicable	

Experimental study design and statistics	Information included in the manuscript?	In which section is the information available? (Reagents and Tools Table, Materials and Methods, Figures, Data Availability Section)
Include a statement about **sample size** estimate even if no statistical methods were used.	Yes	Materials and Methods
Were any steps taken to minimize the effects of subjective bias when allocating animals/samples to treatment (e.g. **randomization procedure**)? If yes, have they been described?	Yes	Materials and Methods
Include a statement about **blinding** even if no blinding was done.	Yes	Materials and Methods
Describe **inclusion/exclusion criteria** if samples or animals were excluded from the analysis. Were the criteria pre-established?<br><br>If sample or data points were omitted from analysis, report if this was due to attrition or intentional exclusion and provide justification.	Yes	Materials and Methods: We excluded from our analysis samples that showed sign of injection-related damages, or eyes that turned out to be not properly injected as assessed from GFP expression analysis.
For every figure, are **statistical tests** justified as appropriate? Do the data meet the assumptions of the tests (e.g., normal distribution)? Describe any methods used to assess it. Is there an estimate of variation within each group of data? Is the variance similar between the groups that are being statistically compared?	Yes	Materials and Methods

Sample definition and in-laboratory replication	Information included in the manuscript?	In which section is the information available? (Reagents and Tools Table, Materials and Methods, Figures, Data Availability Section)
In the figure legends: state number of times the experiment was **replicated** in laboratory.	Yes	Materials and Methods and figure legends
In the figure legends: define whether data describe **technical or biological replicates**.	Yes	Materials and Methods and figure legends

Ethics

Ethics	Information included in the manuscript?	In which section is the information available? (Reagents and Tools Table, Materials and Methods, Figures, Data Availability Section)
Studies involving **human participants**: State details of **authority granting ethics approval** (IRB or equivalent committee(s), provide reference number for approval.	Not Applicable	
Studies involving **human participants**: Include a statement confirming that **informed consent** was obtained from all subjects and that the experiments conformed to the principles set out in the WMA Declaration of Helsinki and the Department of Health and Human Services Belmont Report.	Not Applicable	
Studies involving **human participants:** For publication of **patient photos**, include a statement confirming that consent to publish was obtained.	Not Applicable	
Studies involving experimental **animals**: State details of **authority granting ethics approval** (IRB or equivalent committee(s), provide reference number for approval. Include a statement of compliance with ethical regulations.	Yes	Materials and Methods
Studies involving **specimen and field samples:** State if relevant **permits** obtained, provide details of authority approving study; if none were required, explain why.	Not Applicable	

Dual Use Research of Concern (DURC)	Information included in the manuscript?	In which section is the information available? (Reagents and Tools Table, Materials and Methods, Figures, Data Availability Section)
Could your study fall under dual use research restrictions? Please check biosecurity documents and list of **select agents and toxins** (CDC): https://www.selectagents.gov/sat/list.htm	Not Applicable	
If you used a select agent, is the security level of the lab appropriate and reported in the manuscript?	Not Applicable	
If a study is subject to dual use research of concern regulations, is the name of the **authority granting approval and reference number** for the regulatory approval provided in the manuscript?	Not Applicable	

Reporting

The MDAR framework recommends adoption of discipline-specific guidelines, established and endorsed through community initiatives. Journals have their own policy about requiring specific guidelines and recommendations to complement MDAR.

Adherence to community standards	Information included in the manuscript?	In which section is the information available? (Reagents and Tools Table, Materials and Methods, Figures, Data Availability Section)
State if relevant guidelines or checklists (e.g., **ICMJE, MIBBI, ARRIVE, PRISMA**) have been followed or provided.	Yes	Materials and Methods
For **tumor marker prognostic studies,** we recommend that you follow the **REMARK** reporting guidelines (see link list at top right). See author guidelines, under 'Reporting Guidelines'. Please confirm you have followed these guidelines.	Not Applicable	
For **phase II and III randomized controlled trials**, please refer to the **CONSORT** flow diagram (see link list at top right) and submit the CONSORT checklist (see link list at top right) with your submission. See author guidelines, under 'Reporting Guidelines'. Please confirm you have submitted this list.	Not Applicable	

Data Availability

Data availability	Information included in the manuscript?	In which section is the information available? (Reagents and Tools Table, Materials and Methods, Figures, Data Availability Section)
Have **primary datasets** been deposited according to the journal's guidelines (see 'Data Deposition' section) and the respective accession numbers provided in the Data Availability Section?	Not Applicable	
Were **human clinical and genomic datasets** deposited in a public access-controlled repository in accordance to ethical obligations to the patients and to the applicable consent agreement?	Not Applicable	
Are **computational models** that are central and integral to a study available without restrictions in a machine-readable form? Were the relevant accession numbers or links provided?	Not Applicable	
If publicly available data were reused, provide the respective **data citations in the reference list**.	Yes	Results and references
